# Etiology-specific incidence and mortality of diarrheal diseases in the African region: a systematic review and meta-analysis

**DOI:** 10.1186/s12889-024-19334-8

**Published:** 2024-07-12

**Authors:** Cecilie Thystrup, Shannon E. Majowicz, Dinaol B. Kitila, Binyam N. Desta, Olanrewaju E. Fayemi, Christianah I. Ayolabi, Ephrasia Hugho, Elna M. Buys, Gabriel B. Akanni, Norgia E. Machava, Celso Monjane, Tine Hald, Sara M. Pires

**Affiliations:** 1 Kgs. Lyngby, National Food Institute, Technical University of Denmark, Lyngby, Denmark; 2 School of Public Health Sciences, University of Waterloo, Waterloo, Canada; 3 School of Biological Sciences and Biotechnology, Haramaya University, Dire Dawa, Ethiopia; 4 Centre for Research, Innovation, and Collaboration, Department of Biological Sciences, Mountain Top University, Prayer City, Nigeria; 5 Department of Microbiology, University of Lagos, Lagos, Nigeria; 6 Kilimanjaro Clinical Research Institute, Moshi, Tanzania; 7 Department of Consumer and Food Sciences, University of Pretoria, Pretoria, South Africa; 8 Instituto Superior de Ciencias de Saúde (ISCISA), Maputo, Mozambique; 9 Medical Faculty, Eduardo Mondlane University, Maputo, Mozambique; 10 College of Veterinary Medicine, Haramaya University, Dire Dawa, Ethiopia; 11 Kilimanjaro Christian Medical University College, Moshi, 2021 CRD42021251511 Tanzania

**Keywords:** Diarrheal disease, low-and-middle income countries, Aetiology, Mortality, Diarrheal incidence, Africa

## Abstract

**Background:**

Diarrheal diseases substantially affect public health impact in low- and middle-income countries (LMIC), particularly in Africa, where previous studies have indicated a lack of comprehensive data. With a growing number of primary studies on enteric infections in Africa, this study aimed to estimate the incidence and mortality of diarrheal pathogens across all ages in Africa in the year 2020. We also explored different methodological assumptions to allow comparison with other approaches.

**Methods:**

Through a systematic review and meta-analysis of data from African LMICs, we estimated the etiology proportions for diarrheal diseases and deaths. We combined the etiology proportions with incidence data collected from a population survey in Africa from 2020 and mortality data from the Global Health Observatory of WHO.

**Results:**

We estimated 1,008 billion diarrhea cases (95% UI 447 million-1,4 billion) and 515,031 diarrhea deaths (95% UI 248,983-1,007,641) in the African region in 2020. In children under five, enteroaggregative *E. coli* (EAEC) (44,073 cases per 100,000 people, 95% UI 18,818 − 60,922) and *G. lamblia* (36,116 cases per 100,000 people, 95% UI 15,245 − 49,961) were the leading causes of illness. Enteroinvasive *E. coli* (EIEC) (155 deaths per 100,000 people, 95% UI 106.5-252.9) and rotavirus (61.5 deaths per 100,000 people, 95% UI 42.3-100.3) were the primary causes of deaths. For children over five and adults, *Salmonella* spp. caused the largest number of diarrheal cases in the population of children ≥ 5 and adults (122,090 cases per 100,000 people, 95% UI 51,833 − 168,822), while rotavirus (16.4 deaths per 100,000 people, 95% UI 4.2–36.7) and enteroaggregative *E. coli* (EAEC) (14.6 deaths per 100,000 people, 95% UI 3.9–32.9) causing the most deaths. Geographically, the highest incidence of diarrhea was in Eastern Africa for children under five (114,389 cases per 100,000 people, 95% UI 34,771 − 172,884) and Central Africa for children over five and adults (117,820 cases per 100,000 people, 95% UI 75,111–157,584). Diarrheal mortality was highest in Western Africa for both children below five and above (children < 5: 194.5 deaths per 100,000 people, 95% UI 120-325.4; children ≥ 5 and above: 33.5 deaths per 100,000 people, 95% UI 12.9–75.1).

**Conclusion:**

These findings provide new information on the incidence and mortality of sixteen pathogens and highlight the need for surveillance and control of diarrheal infectious diseases in Africa. The cause-specific estimates are crucial for prioritizing diarrheal disease prevention in the region.

**Supplementary Information:**

The online version contains supplementary material available at 10.1186/s12889-024-19334-8.

## Introduction

Diarrheal diseases have a substantial public health impact in low- and middle-income countries (LMICs), particularly in Africa. The World Health Organization (WHO) estimates that every year, more than 1.7 billion cases of diarrhoea occur worldwide, and that around 443,832 children under five lose their lives to diarrhoea each year [1]. The combination of poor nutrition and sanitation, insufficient access to medical care and clean water, and extensive exposure to contaminated sources and environments makes people living in LMICs particularly vulnerable to enteric diseases [2].

Enteric pathogens can be transmitted through contaminated food, water, and direct or indirect contact with infected persons or animals [3–5]. Efficient control of diarrheal diseases requires knowledge of the most important etiologies of diarrhoea in specific populations and geographical areas, but data on the incidence of diarrhoeal etiologies in the general population in LMICs are scarce due to lack of surveillance [6]. In Africa specifically, prior attempts to synthesize existing evidence on diarrhea etiology noted a scarcity of data [7]. While there has been a surge in primary studies on enteric infections in recent years, these investigations have predominantly centered on individual countries and have not been integrated into a comprehensive synthesis for estimating causative agents across regions. Furthermore, the focus of these studies has primarily been on children under five years old. For example, the multisite birth cohort study (MAL-ED) from 2015 [8] estimated pathogen-specific incidence of diarrhoea in eight different sites in LMICs across the world, including South Africa. The Global Enteric Multicenter Study (GEMS) [9] from 2012 used stool samples from a case-control study to identify potential causes of diarrhea and determine the burden of these diseases. Both studies focused largely on children under five, which is often the most represented group in primary studies conducted in the African region. Although children below five still account for most of the disease burden, there has been a shift in disease from younger children to older children and adolescents, which has largely been driven by the considerable reduction in the former group and slower progress in the latter [10]. Thus, there is a need for new studies that synthesize literature across all age groups in an African setting.

Other studies have synthesized the existing literature on diarrheal etiologies in Africa. The Global Burden of Diseases, Injuries, and Risk Factors Study (GBD) from the Institute of Health Metrics and Evaluation (IHME) applies hospital inpatient, outpatient, and community studies’ data records of diarrheal cases to estimate the diarrheal incidence and mortality. It applies a population-attributable fraction (PAF) approach to estimate etiology-specific diarrheal incidence and mortality [11]. No studies have, to the best of our knowledge, presented etiology-proportions of diarrhea in Africa using alternative methods. The Maternal Child Epidemiology Estimation (MCEE) group applies an alternative approach to estimate etiology-specific mortality, which has a more stringent inclusion and exclusion criteria for data. The two approaches have provided well-accepted results that have guided policy recommendations [12–14]. In recent years, new studies have presented the prevalence of different diarrheal agents in Africa, thus adding more data to the data gap. These studies have been conducted both before, during and after the global pandemic of COVID-19, and it would be interesting to see if this have impacted the prevalence of foodborne and diarrhea causing pathogens in Africa. Because of all of these reasons, we believe that there is a need for an updated analysis of the available literature.

In this study, our objective was to estimate the etiology-specific incidence and mortality of diarrhea in the general population in Africa (all ages) in the reference year 2020 and explore different methodological assumptions to allow comparison with other approaches. Specifically, we were interested in diarrhea etiologies across the whole population, meaning all people of all ages, comorbidities, and risk factors.

## Methodology

### Overall approach

We conducted a systematic review and meta-analysis to determine diarrheal disease etiology, and applied etiology fractions to the incidence of diarrhea in the general population. The incidence of diarrhea was estimated from a population survey of conducted in all ages in four African countries in 2020–2021, and the diarrhea mortality data extracted from the WHO’s Global Health Observatory (GHO) [15]. We followed the general methodology of Pires et al. (2015) [7] to create estimates for both under five and above 5-year age groups. Given that some pathogens considered can be present in young children without being the cause of diarrhea [16], we also created estimates for children 0–4 years that adjusted for potential carriage of pathogens unrelated to diarrhea, following the methodology of the Maternal Child Epidemiology Estimation (MCEE) group [17]. We also explored the impact of adjusting our estimates for potential carriage of pathogens unrelated to diarrhea in those above years. Finally, we explored the impact of choice of diarrheal incidence estimate on our results.

### Systematic review search strategy and selection criteria

A systematic review (SR) was conducted in accordance with the Guidelines for Accurate and Transparent Health Estimates Reporting (GATHER) [18]. using a review protocol developed specifically for the project. Before developing the SR protocol, we searched previously conducted SRs in African countries of children with diarrhoea in the PROSPERO Registry. One potentially relevant SR and meta-analysis was found, but since it focused on collecting global data on diarrhoeal diseases in children aged zero to ten years, it was deemed relevant to conduct our review (all ages) (PROSPERO CRD42020204005). Our protocol was registered with PROSPERO in accordance with PRISMA guidelines (PROSPERO CRD42021251511). After reviewing enteric infectious disease causes, the following 17 pathogens were included in the SR: rotavirus, norovirus, astrovirus, *Giardia lamblia*,* Entamoeba histolytica*,* Cryptosporidium* spp., *Campylobacter* spp., *Salmonella* spp., *Shigella* spp., *Vibrio cholerae*, *Staphylococcus aureus*, enterotoxigenic *Escherichia coli* (ETEC), enteropathogenic *Escherichia coli* (EPEC), enteroinvasive *Escherichia coli* (EIEC), enterohemorrhagic *Escherichia coli* (EHEC), enteroaggregative *Escherichia coli* (EAEC) and Shiga-toxin producing *Escherichia coli* (STEC) [7, 19–21]. Any studies that reported pathogens causing diarrhea not included in our list were grouped into an “other” category. For studies that investigated > 8 pathogens and collected data on the type of diagnostic tool used to detect diarrhea, we also extracted data on the proportion of diarrhea due to unknown causes.

A targeted literature search was performed in June 2021. Databases like PubMed (all-fields), Scopus (only title-abstract-keywords; excluding conference papers, notes, editorials, and letters), Web of Science (only core collection; excluding proceeding papers, meeting abstracts, news items, editorial material and letters) were searched automatically using different combinations of “diarrhoea”, “diarrhea”, “aetiology”, ”pathogen”, “mortality”, “cause of death”, “gastroenteritis” and “incidence”. Each search string was tailored to fit the databases individually (Supplementary Material: Search string 1, 2 and S1). To broaden the search and minimise the risk of publication bias, grey literature was hand-searched and included in the SR. This included hand-searched literature from Google.

All types of publications collecting diarrhoeal samples or testing for diarrhoeal agents in outpatient, inpatient, and community settings were included in the SR. This included prospective studies for morbidity and mortality. Surveillance reports were also included, provided that they included at least 12 months of data, to account for seasonal variation in the prevalence of the pathogens. We included studies published between January 1st 2014 and April 30th 2021. Studies conducted before January 1st 2014 were excluded, unless the study presented not-previously published data. Publications in all languages were marked as eligible and were translated using Google Translate or by researchers familiar with the language in question and involved in the screening process.

Studies conducted in animal or non-African populations were excluded. Because our goal was to identify the relative contribution of causes in the general population, we excluded studies that only reported results for one segment of the population known to have different relative etiologies than the overall general population (i.e., travellers, HIV patients, and nosocomial, chronic, antibiotic-induced or outbreak diarrhoea). While these individuals were part of the studies we did include, we excluded studies focusing solely in these sub-groups to ensure representitaviness of the general population. Any case reports (individual or outbreak), and studies with a recall period of more than four weeks were excluded. Finally, duplicate articles were removed using EndNoteX9 software and articles not fulfilling the predetermined eligibility criteria were excluded based on title and abstract screening and full-text screening.

All articles were assessed by pairs of reviewers from the FOCAL project. At first, the reviewers screened a subset of the articles together to ensure agreement on how to apply the exclusion criteria. The remaining articles were then screened by each reviewer individually. Any disagreements were resolved by a third reviewer. Articles were excluded if two or more reviewers agreed. Articles with incomplete or inaccessible information were excluded as well. Data extraction was carried out by one reviewer and extracted to Microsoft Excel, in compliance with the GATHER guidelines. The number of diarrhoeal samples positive for the pathogens of interest, and the total number of diarrhoeal samples were extracted. Other data extracted from the studies included the first author’s surname, the year of publication, study period, country, region, type of study, study design and age group(s), and diagnostic test used. Studies reporting co-infections were not treated separately and were therefore included in the number of positive samples for each pathogen of interest. Studies were stratified based on study setting (out-patient, in-patient, community), age group (above or below five years of age) and whether rotavirus vaccination was occurring in the setting during the study. Articles presenting studies conducted in more than one of the specified settings were investigated thoroughly and the data referred accordingly. Articles presenting their results stratified by age or age-group were combined or split into the subgroups matching our study.

### Statistical analysis of diarrhea etiology

A random-effects model was fitted on the number of positive samples for every given pathogen to pool effect sizes, as we anticipated considerable between-study heterogeneity. The restricted maximum-likelihood estimator was used to calculate the heterogeneity variance ${\tau }^{2}$, and Knapp-Hartung adjustments were used to calculate the confidence interval around the pooled effect [22]. To avoid overestimation of the true effect sizes, the data was logit-transformed before pooling [23]. A chi-squared test was performed with a significance level of 0.1 to determine the statistical significance of the estimated proportions. A significance level of 0.1 was chosen to increase the sensitivity of the analysis and enhance the detection of variations in effect size [24]. Outliers were inspected using forest plots and identified using the leave-one-out method, where the pooled effects were recalculated after omitting one study at a time [25]. Publication bias was also assessed using funnel plots, and for pathogens with 10 or more studies contributing to its pooled effect size, Egger’s Regression Test was used to test for asymmetry in the plot [26]. A moderator analysis was carried out due to the anticipation of between-study heterogeneity by fitting a mixed-effect model to the data. The studies were separated by pathogen and split into two or more subgroups, based on the moderator variables identified *a priori.* These variables were selected based on the assumption that the prevalence of the different pathogens would vary between regions and study-settings and could hypothetically increase or decrease the risk of bias. Expected moderators included study setting and age-group of the individuals involved in the study. Other moderators included the geographical area from which the study had been conducted in (e.g., rural, urban) and the African region (e.g., Northern, Southern, etc.).

The meta-analysis was conducted in R (version 4.3.1, 2023-06-16), using the Meta package (version 5.1.0).

### Estimation of diarrhoeal- and diarrhoeal mortality envelopes

To estimate the etiology-proportions of diarrhoeal cases and deaths in the general community, we first collected the diarrhoeal incidence and mortality “envelopes” (i.e. the total diarrhoeal incidence and mortality in each country) for children and adults. Diarrheal case-envelopes were collected from Desta et al. [27], which estimated diarrheal incidence in the general African population using empirical data from Ethiopia, Mozambique, Nigeria, and Tanzania in 2020–2021 [27]. Diarrhoeal mortality envelopes were collected from the GHO and WHO for the year 2019 [15]. We chose these estimates as the most current ones available on diarrhea incidence and mortality in Africa at the time of our study.

To estimate the diarrheal cases and deaths envelopes, we used the number of diarrheal cases in the age groups below and above five years of age to calculate the annual adjusted incidence and mortality rate. We used the age-standardized adjusted incidence rate from Desta et al. to represent the diarrhoeal incidence rate for each region by multiplying the population estimates from GHO with the diarrheal incidence rate from Desta et al. We assumed that the incidence rate in each region was applicable to all countries in that region.

We used the incidence rate for Nigeria to represent the diarrhoeal incidence envelope for Western Africa. We used the average age-adjusted incidence rate of Tanzania, Mozambique, and Ethiopia to represent the incidence rate of Eastern Africa. For the Northern, Southern and Central region, we used the overall age-standardized adjusted incidence rate. Then, population estimates from GHO were summed for each region and multiplied with those incidence proportions.

To estimate the regional mortality estimates, the country-specific diarrhoeal mortality rates obtained from the WHO GHO were multiplied with the country population estimates and summed for each region. Only WHO Member states and non-disputed territories with population estimates larger than 90,000 were included in the subregions, which excluded Réunion, Western Sahara, Mayotte and Saint Helena.

The age-adjusted annual incidence rate of diarrhoea in Ethiopia, Mozambique, Nigeria, and Tanzania from Desta et al. were given in per person-year and the population estimates obtained from GHO in people-per-year (Supplementary Table S10).

### Estimation of etiology proportions

To estimate the etiology-proportions for diarrhoeal cases, we assumed that the distribution of pathogens observed in out-patient and community settings represented the general distribution of pathogens in diarrhoeal cases. For diarrhoeal deaths, we assumed a distribution of pathogens as observed in in-patient settings. To adjust our estimates for potential carriage of pathogens unrelated to diarrhea, we used pathogen positivity according to the GEMS qPCR study to define pathogenicity, as described in Liu et al. (2016) [16]. Pathogens that were not associated with causing diarrhea in the GEMS qPCR re-analysis were excluded from our etiology estimates. Thus, we excluded norovirus G1, atypical EPEC and LT-ETEC, when estimating diarrhoeal etiology proportions, and assumed that all cases of diarrhoea were attributable to one of the 16 pathogens (including other and unknown etiology) included in this study. We could not exclude data from studies that did not distinguish between subtypes of norovirus, EPEC and ETEC.

To ensure that all pathogens of interest were included in the etiology-estimates for both diarrhoeal illnesses and deaths, and to account for data gaps, we imputed missing values for given pathogens from other study-settings, where data were available.

To account for the influence of rotavirus vaccination on the diarrheal etiologies, the pooled proportions for rotavirus were calculated separately based on vaccination status. This meant that studies presenting rotavirus estimates conducted in countries where rotavirus vaccine had already been implemented were pooled together. For countries without an implemented vaccine programme, we used the pooled proportions estimated in countries without a vaccine [28].

To ensure that the pooled effect sizes did not sum to more than 100%, univariate beta-distributions were fitted to the pooled estimates and quantiles using one-dimensional optimisation where the squared distance between the estimated and fitted quantiles were minimized and the proportions of the fitted distribution were similar to the estimated proportions. 10,000 random deviates where then sampled from the fitted beta-distributions, and the random deviates were normalised iteration-wise by dividing with the sum of etiological fractions for each row.

### Estimation of etiology-specific incidence and mortality

The etiology-specific incidence and mortality were estimated by multiplying the normalised proportions of each pathogen with the diarrhoeal incidence and mortality envelope, respectively, using a stochastic model with 10,000 iterations. The diarrheal incidence and mortality envelopes were then calculated per 100,000 people by dividing the number of illnesses or deaths attributed to each pathogen with the total population for that region and multiplying the number with 100,000. Due to lack of data, the etiology proportions were calculated per pathogen for the whole African region and not individually for each region.

The model was applied in R (version 4.3.1, 2023-06-16).

### Sensitivity analysis

To explore the impact of our choice of diarrhea incidence and mortality envelopes, we applied the model using the country-specific diarrheal incidence and diarrhoeal mortality published by GBD 2019, which were collected from the GBDx Dashboard [11]. (Available from https://vizhub.healthdata.org/gbd-compare/), and compared the two sets of results (Supplementary Table S11).

## Results

### Etiology proportions of diarrhoea cases and deaths

**Fig. 1:**
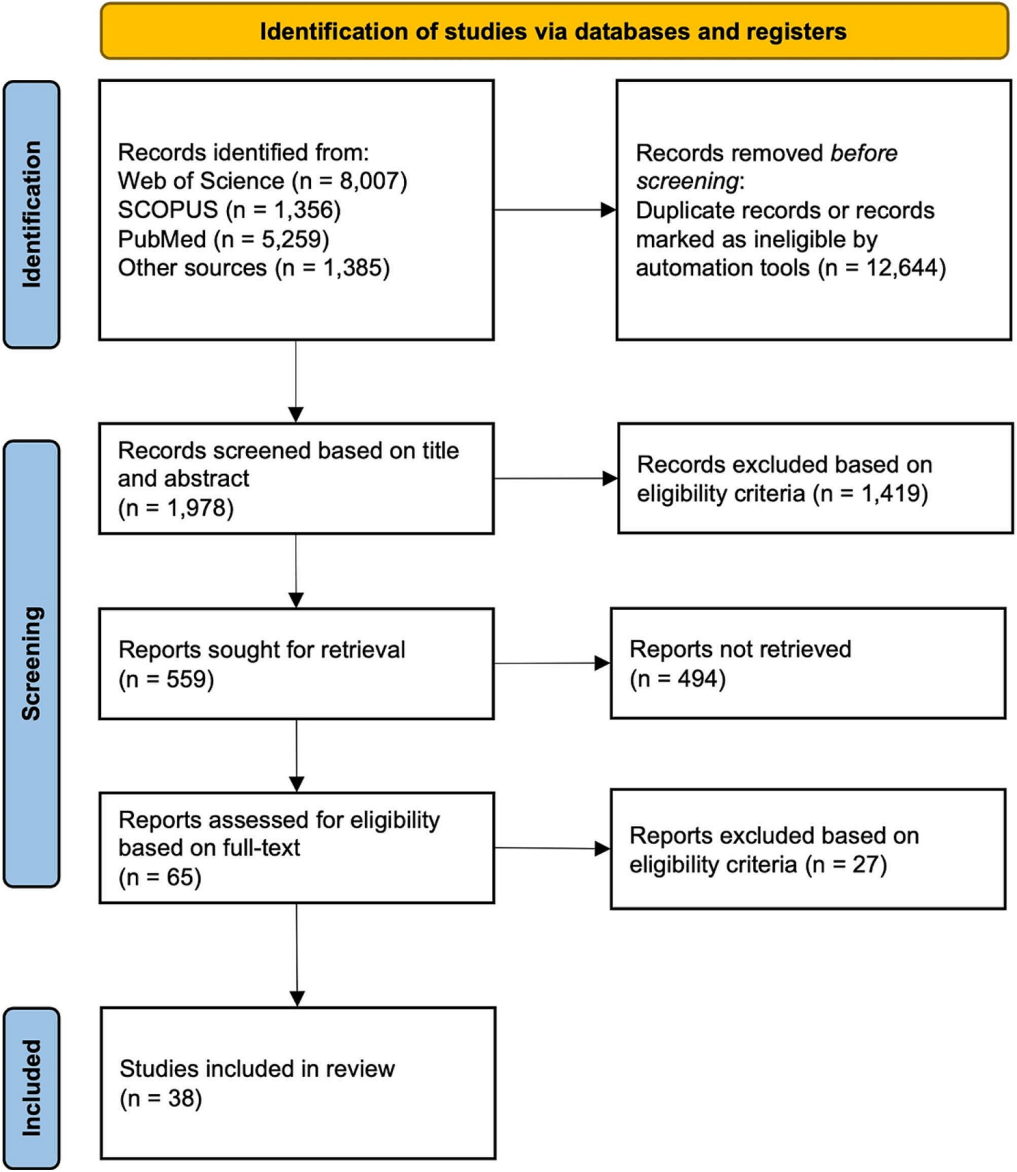
PRISMA Flowchart showing the process of identifying eligible articles included in the SR. n = number of articles

We identified 38 eligible articles from the SR, from which data from individual 414 study-settings were extracted (Fig. 1).

Nineteen articles included more than one combination of age groups, study-setting and/or country. Studies were conducted in 17 different countries, representing all five regions of Africa. Ten studies were conducted exclusively in in-patient facilities, whereas 21 studies were conducted in out-patient and/or community settings. Seven articles included more than one study-setting (e.g. both in-patient and out-patient settings). Twenty-eight studies focused exclusively on children under five years of age, whereas the remaining ten studies included individuals of all age groups. Thirty-five studies used molecular diagnostic tools (92%; 35/38) i.e. polymerase chain reaction (PCR), ELISA or immunoassays. The remaining three studies used microscopy for detection of pathogens. All studies were divided into appropriate subgroups and included in the meta-analysis (Supplementary Table S2-S3).

### Pooled etiology proportions of diarrhoea cases and deaths

We identified high between-study heterogeneity and variability in the majority of the pooled estimates (Supplementary Table S4-S7). For each pathogen, the number of influential cases removed was noted. The final number of studies providing data for individual pathogens in different regions varied substantially, which resulted in some pathogens not having a reported estimate. For some pathogens, only one study was available, so no p-value could be calculated for these estimates. In children < 5, the pooled proportions were highest for EIEC (g = 0.8, 95% UI 0.46–0.95) in children < 5 in inpatient settings and EAEC (g = 0.16, 95% UI 0.1–0.24) in outpatient and community settings. In the population above five, EIEC had the highest pooled proportion. In outpatient and community setting, ETEC had the highest pooled proportion for children above 5 and adults (g = 0.79, 95% UI 0.05–0.12).

The pooled proportions of rotavirus in in-patient settings for children under five were similar for all countries, despite vaccination status (Vaccination: g = 0.28, 95% UI 0.19–0.4. No vaccination: g = 0.29, 95% UI 0.2–0.43) (Supplementary Table S4-S5). In contrast, in outpatient and community settings for children under five where rotavirus vaccination had been introduced in the population, the pooled proportions were much lower than settings where the vaccine had not yet been implemented (Vaccination: g = 0.05, 95% UI 0.01–0.22. No vaccination: g = 0.21, 95% UI 0.14–0.31) (Supplementary Table S6-S7). Thus, we assumed that rotavirus vaccination would only impact the etiology proportion of rotavirus diarrhea incidence, not mortality.

The estimated etiology proportions of norovirus, EPEC and ETEC before adjusting for potential carriage of pathogens unrelated to diarrhea, showed very little change when compared to the non-adjusted values (Supplementary Table S8). The adjusted proportions were marginally smaller than the non-adjusted values for all age-groups except the estimates for the out-patient and community settings, for which the pooled proportions were higher for the adjusted values. Thus, we selected the adjusted pooled proportions for the estimation of etiology-specific incidence and mortality.

The study-level etiology proportions were influenced by variation in effect size, which could not be attributed to chance alone (*p* < 0.1). Assessment of publication bias by inspection of the funnel plots showed minimal asymmetry for most of the estimates, suggesting publication bias in the estimates, but the insignificant p-value from the Egger’s regression test for pathogens with ten or more studies could not confirm this (Supplementary Table S9).

### Estimated numbers of diarrheal cases and deaths by pathogen

The estimated number of diarrheal cases and deaths by region in Africa, adjusted for potential carriage of pathogens unrelated to diarrhea, are shown for both age groups (below five and above five years of age) in Tables 1 and 2. We estimated that the 16 pathogens along with the *other* and *unknown* pathogens accounted for a total of 1,008,712,693 diarrhea cases (95% UI 447 million-1,4 billion) and 515,031 diarrhea deaths (95% UI 248,983-1,007,641) in 2020 in the African region. Eastern Africa had the largest number of diarrheal cases in both children below five (76,493,449 cases, 95% UI 23,252,270 − 115,609,415) and the population above five (418,379,492 cases, 95% UI 127,177,857 − 632,323,553). Western Africa had the largest number of diarrheal deaths for both children < 5 (124,924 deaths, 95% UI 77,087–208,957) and the population above five (109,894 deaths, 95% UI 41,821 − 245,807). Southern Africa both had the lowest number of diarrheal cases and deaths for both age groups as well (diarrheal cases for children < 5 five: 5,430,774 cases, 95% UI 3,462,117-7,263,660; diarrheal cases for the population > 5: 47,913,915 cases, 95% UI 30,545,119 − 64,084,862; diarrheal deaths for children < 5: 3,878 deaths, 95% UI 2,956-5,068; diarrheal deaths for population ≥ 5: 16,010 deaths, 95% UI 6,139 − 37,152).

**Table 1:** Total estimates of diarrheal illnesses and deaths per etiology in for children < 5 years of age in African regions, with 95% uncertainty interval (UI)

Pathogen	Northern Africa		Central Africa		Eastern Africa		Southern Africa		Western Africa		Whole African Region (197 mil. Pop)	
	6 countries (29 mil. Pop)		9 countries (30 mil. Pop)		18 countries (67 mil. Pop)		5 countries (7 mil. Pop)		16 countries (64 mil. Pop)			
	Illnesses (95% UI)	Deaths (95% UI)	Illnesses (95% UI)	Deaths (95% UI)	Illnesses (95% UI)	Deaths (95% UI)	Illnesses (95% UI)	Deaths (95% UI)	Illnesses (95% UI)	Deaths (95% UI)	Illnesses (95% UI)	Deaths (95% UI)
Rotavirus	1,261,204 (804,018–1,686,861)	5,675 (4,263–10,360)	2,694,151 (1,717,521-3,603,427)	1,042 (737-1,341)	3,117,536 (947,660-4,711,731)	6,134 (4,054–9,185)	191,684 (122,198–256,377)	481 (367–629)	2,192,972 (1,461,981-2,857,509)	14,284 (8,805–23,994)	9,457,547 (4,931,180–13,115,905)	27,616 (18,226–45,459)
Norovirus	1,630,384 (1,039,369-2,180,638)	1,745 (1,305-3,168)	1,673,471 (1,066,838-2,238,267)	337 (241–427)	5,104,001 (1,551,500-7,714,001)	1,851 (1,200-2,784)	360,652 (229,916–482,373)	139 (106–182)	1,489,145 (992,763-1,940,401)	5,063 (3,128-8,444)	10,257,653 (4,650,470–14,555,680)	9,135 (5,980–15,005)
Astrovirus	388,405 (247,608–519,491)	85 (64–155)	491,833 (313,544–657,827)	17 (12–21)	1,160,176 (352,667-1,753,448)	90 (58–135)	80,033 (51,021–107,044)	7 (5–9)	424,215 (282,810–552,765)	258 (160–430)	2,544,662 (1,196,629-3,590,575)	457 (299–750)
*Campylobacter* spp.	962,476 (613,578-1,287,311)	1,447 (1,078–2,613)	847,881 (540,524-1,134,041)	293 (211–366)	3,094,622 (940,695-4,677,099)	1,505 (956-2,274)	221,382 (141,131–296,099)	108 (82–141)	775,659 (517,106-1,010,707)	4,755 (2,944-7,899)	5,902,020 (2,611,903-8,405,257)	8,108 (5,271–13,293)
*Cryptosporidium* spp.	930,927 (593,466-1,245,115)	4,337 (3,249-7,890)	997,736 (636,056–1,334,471)	823 (586-1,048)	2,887,954 (877,872-4,364,748)	4,631 (3,025–6,955)	203,081 (129,464–271,621)	354 (270–462)	882,147 (588,098–1,149,465)	11,976 (7,395–20,008)	5,901,845 (2,695,492-8,365,420)	22,121 (14,525–36,363)
*Entamoeba histolytica*	133,565 (85,148–178,643)	292 (217–524)	176,513 (112,527–236,086)	64 (46–78)	394,595 (119,948–596,376)	295 (181–449)	27,052 (17,246–36,182)	20 (15–26)	151,370 (100,913–197,240)	1,131 (702-1,871)	883,095 (418,536-1,244,527)	1,802 (1,161-2,948)
*Giardia lamblia*	2,204,350 (1,405,273-2,948,318)	1,910 (1,436-3,490)	2,419,539 (1,542,456-3,236,133)	348 (246–449)	6,804,770 (2,068,495–10,284,481)	2,070 (1,372-3,098)	477,339 (304,304–638,441)	163 (125–214)	2,131,260 (1,420,840-2,777,096)	4,707 (2,900-7,897)	14,037,258 (6,437,064–19,884,469)	9,198 (6,079–15,148)
EHEC	486,212 (309,960–650,308)	886 (658-1,594)	375,337 (239,277–502,013)	182 (132–225)	1,594,485 (484,687-2,409,846)	906 (569-1,373)	115,218 (73,451–154,103)	63 (48–83)	348,714 (232,476–454,385)	3,039 (1,883-5,040)	2,919,966 (1,266,400-4,170,655)	5,076 (3,290-8,315)
EPEC	1,209,502 (771,057–1,617,708)	2,280 (1,702-4,129)	1,158,562 (738,583-1,549,576)	450 (323–567)	3,833,690 (1,165,354-5,794,099)	2,396 (1,540-3,612)	272,409 (173,660–364,346)	176 (134–230)	1,043,754 (695,836-1,360,043)	7,015 (4,338–11,676)	7,517,917 (3,370,830–10,685,772)	12,317 (8,037–20,214)
ETEC	1,626,278 (1,036,752-2,175,147)	1,424 (1,069–2,598)	1,499,366 (955,846-2,005,402)	263 (186–338)	5,189,400 (1,577,460-7,843,071)	1,534 (1,012–2,299)	369,920 (235,824–494,768)	120 (91–156)	1,359,989 (906,659-1,772,106)	3,647 (2,249-6,109)	10,044,953 (4,476,717–14,290,494)	6,988 (4,607–11,500)
EAEC	2,666,531 (1,699,913-3,566,485)	3,978 (2,970-7,204)	3,071,346 (1,957,983-4,107,925)	785 (563–988)	8,145,850 (2,476,153–12,311,342)	4,182 (2,688-6,304)	568,433 (362,376–760,280)	308 (235–402)	2,685,426 (1,790,284-3,499,192)	12,218 (7,557–20,339)	17,137,586 (7,924,333–24,245,224)	21,471 (14,013–35,237)
EIEC	542,522 (345,858–725,623)	14,135 (10,607–25,770)	1,060,452 (676,038–1,418,354)	2,630 (1,865-3,370)	1,400,216 (425,634-2,116,236)	15,206 (10,004–22,796)	88,525 (56,434–118,402)	1,181 (900-1,543)	870,049 (580,032–11,337,00)	36,956 (22,796–61,862)	3,961,764 (2,027,562-5,512,315)	70,108 (46,172–115,341)
STEC	1,253,608 (799,175-1,676,700)	161 (122–299)	1,822,410 (1,161,786-2,437,473)	26 (18–35)	3,605,364 (1,095,949-5,449,016)	183 (126–271)	243,703 (155,361–325,953)	16 (12–21)	1,543,871 (1,029,247-2,011,711)	247 (150–424)	8,468,956 (4,086,157–11,900,853)	633 (428-1,050)
*V. cholerae*	8,526 (5,436–11,404)	1,031 (783-1,910)	29,321 (18,692–39,217)	162 (111–220)	14,498 (4,407–21,912)	1,172 (812-1,735)	606 (386–810)	102 (78–133)	23,082 (15,388–30,077)	1,501 (913-2,588)	76,033 (43,923–103,420)	3,968 (2,697-6,586)
*Salmonella* spp.	133,952 (85,394–179,161)	191 (145–354)	179,897 (114,684–240,612)	29 (20–40)	394,364 (119,878–596,027)	219 (153–323)	26,963 (17,189–36,062)	19 (15–25)	153,996 (102,664–200,662)	242 (146–421)	889,172 (422,620-1,252,524)	700 (479-1,163)
*Shigella* spp.	310,255 (197,787–414,966)	2,002 (1,496-3,631)	409,336 (260,952–547,487)	390 (279–493)	916,715 (278,661-1,385,490)	2,116 (1,368-3,185)	62,868 (40,078–84,086)	158 (120–206)	351,072 (234,048–457,457)	5,930 (3,666-9,883)	2,050,246 (971,448-2,889,486)	10,596 (6,929–17,398)
Other	1,815,677 (1,157,494-2,428,468)	1,976 (1,483-3,603)	1,504,751 (959,279-2,021,605)	366 (259–470)	5,889,884 (1,790,391-8,901,756)	2,129 (1,403-3,190)	423,402 (269,919–566,301)	166 (126–217)	1,392,308 (928,206-1,814,220)	5,101 (3,146-8,543)	11,026,022 (4,835,370–15,723,350)	9,738 (6,417–16,023)
Unknown	6,620,377 (4,220,490-8,854,754)	3,303 (2,495-6,067)	2,984,271 (1,902,473-3,991,462)	566 (395–742)	22,945,329 (6,974,859–34,678,736)	3,597 (2,425-5,360)	1,697,504 (1,082,159-2,270,412)	297 (227–389)	3,219,343 (2,146,229-4,194,902)	6,854 (4,209–11,579)	37,466,824 (15,244,051–53,990,266)	14,617 (9,751–24,137)
Total	24,184,751 (15,417,776–32,347,101)	46,858 (35,142–85,359)	23,396,173 (14,915,059–31,292,378)	8,773 (6,230–11,218)	76,493,449 (23,252,270–115,609,415)	50,216 (32,946–75,328)	5,430,774 (3,462,117-7,263,660)	3,878 (2,956-5,068)	21,038,372 (14,025,580–27,413,638)	124,924 (77,087–208,957)	150,543,519 (67,610,685–213,926,192)	234,649 (154,361–385,930)

**Table 2:** Total estimates of diarrheal illnesses and deaths per etiology in children ≥ 5 years of age and adults in African regions, with 95% uncertainty interval (UI)

Pathogen	Northern Africa		Central Africa		Eastern Africa		Southern Africa		Western Africa		Whole African Region (1,1 bil. pop)	
	6 countries (212 mil. pop)		9 countries (144 mil. pop)		18 countries (366 mil. pop)		5 countries (60 mil. pop)		16 countries (327 mil. pop)			
	Illnesses (95% UI)	Deaths (95% UI)	Illnesses (95% UI)	Deaths (95% UI)	Illnesses (95% UI)	Deaths (95% UI)	Illnesses (95% UI)	Deaths (95% UI)	Illnesses (95% UI)	Deaths (95% UI)	Illnesses (95% UI)	Deaths (95% UI)
Rotavirus	2,807,136 (1,789,549-3,754,544)	6,261 (2,074–14,221)	10,519,182 (6,705,978–14,069,406)	505 (152-1,227)	6,982,238 (2,122,442–10,552,701)	16,764 (5,808–36,155)	664,452 (423,588–888,705)	2,582 (990-5,991)	5,882,294 (3,921,529-7,664,808)	13,474 (5,043–30,185)	26,855,302 (14,539,498–36,930,164)	39,586 (13,077–87,779)
Norovirus	3,280,685 (2,091,437-4,387,917)	1,033 (348-2,325)	5,137,772 (3,912,830-8,209,270)	92 (28–223)	11,208,783 (3,407,215–16,940,548)	2,540 (877-5,488)	1,263,603 (805,547-1,690,069)	376 (144–872)	3,708,243 (2,472,162-4,831,953)	2,693 (1,027–6,023)	25,599,086 (11,883,644–36,059,757)	6,734 (2,280–14,931)
Astrovirus	793,674 (505,967-1,061,539)	83 (30–178)	1,843,767 (1,175,401-2,466,038)	11 (3–26)	2,555,119 (776,698-3,861,713)	119 (40–261)	276,530 (176,288–369,859)	11 (4–26)	1,083,064 (722,043–1,411,266)	399 (158–888)	6,552,154 (3,180,109-9,170,415)	623 (231-1,379)
*Campylobacter* spp.	1,898,745 (1,210,450-2,539,571)	445 (145-1,018)	3,043,231 (1,940,060–4,070,321)	33 (10–79)	6,772,687 (2,058,743–10,235,992)	1,273 (442-2,741)	773,010 (492,794-1,033,901)	201 (77–467)	1,880,855 (1,253,903-2,450,811)	781 (285-1,753)	14,368,528 (6,463,156–20,330,596)	2,733 (882-6,058)
*Cryptosporidium* spp.	1,018,558 (649,331-1,362,321)	806 (272-1,813)	2,440,224 (1,555,643-3,263,799)	72 (22–175)	3,190,395 (969,808-4,821,847)	1,972 (681-4,262)	342,055 (218,060–457,498)	291 (112–675)	1,422,096 (948,064)	2,129 (813-4,761)	8,413,328 (4,122,846–11,758,499)	5,270 (1,788–11,686)
*Entamoeba histolytica*	2,689,045 (1,714,266-3,596,598)	1,002 (338-2,256)	7,728,796 (4,927,107–10,337,264)	89 (27–217)	7,873,722 (2,393,433–11,900,058)	2,461 (850-5,318)	821,475 (523,690-1,098,722)	364 (139–844)	4,427,421 (2,951,614-5,769,064)	2,631 (1,004–5,884)	23,540,459 (11,986,420–32,701,706)	6,547 (2,219–14,519)
*Giardia lamblia*	7,012,029 (4,470,169-9,378,589)	2,161 (728-4,863)	13,562,067 (8,645,817–18,139,264)	193 (59–468)	23,731,508 (7,213,839–35,866,938)	5,294 (1,828–11,440)	2,660,250 (1,695,909-3,558,084)	782 (300-1,814)	8,137,830 (5,425,220–10,603,839)	5,679 (2,167–12,698)	55,103,684 (25,755,045–77,546,714)	14,109 (4,782–31,283)
EHEC	1,025,576 (653,805-1,371,708)	1,368 (495-2,954)	1,563,230 (996,559-2,090,820)	175 (55–425)	3,694,056 (1,122,909-5,583,062)	2,014 (675-4,418)	421,629 (268,788–563,928)	199 (76–461)	975,536 (650,358-1,271,153)	6,479 (2,568–14,434)	7,680,027 (3,423,631–10,880,671)	10,235 (3,793–22,692)
EPEC	2,302,435 (1,467,803-3,079,507)	3,455 (1,127-7,911)	3,728,697 (2,377,044–4,987,132)	252 (75–612)	8,177,012 (2,485,626–12,358,439)	9,933 (3,451–21,393)	927,784 (591,462-1,240,911)	1,574 (604-3,653)	2,301,543 (1,534,362-2,998,980)	5,924 (2,157–13,305)	17,437,471 (7,864,835–24,664,969)	21,138 (6,810–46,874)
ETEC	5,205,410 (3,318,449-6,962,236)	2,322 (790-5,197)	8,717,855 (5,557,632–11,660,131)	219 (68–533)	18,301,882 (5,563,356–27,660,799)	5,386 (1,855–11,653)	2,086,982 (1,330,451-2,791,339)	773 (296-1,794)	5,357,681 (3,571,787-6,981,221)	6,774 (2,607–15,135)	39,669,810 (18,011,224–56,055,726)	15,474 (5,320–34,312)
EAEC	3,430,849 (2,187,166-4,588,760)	5,611 (1,870–12,705)	6,388,801 (4,072,861-8,545,021)	469 (142-1,140)	11,686,164 (3,552,328–17,662,043)	14,614 (5,058–31,537)	1,296,955 (826,809-1,734,677)	2,220 (851-5,152)	3,864,983 (2,576,655-5,036,190)	12,998 (4,898–29,078)	26,667,752 (12,389,010–37,566,691)	35,902 (11,968–79,612)
EIEC	499,447 (318,397–668,010)	3,147 (1,089–6,977)	843,175 (537,524-1,127,747)	326 (101–791)	1,758,390 (534,511-2,657,567)	6,580 (2,254–14,273)	198,965 (126,840–266,116)	888 (341-2,062)	516,277 (344,185–672,725)	10,730 (4,176–23,949)	3,816,254 (1,734,617-5,392,165)	21,671 (7,620–48,052)
STEC	7,896,293 (5,033,887–10,561,291)	162 (53–371)	8,885,791 (5,664,692–11,884,746)	12 (4–29)	29,918,066 (9,094,412–45,217,078)	460 (160–992)	3,483,187 (2,220,532-4,658,763)	73 (28–169)	5,877,389 (3,918,259-7,658,416)	292 (107–655)	56,060,726 (23,711,250–79,980,294)	999 (324-2,216)
*V. cholerae*	1,947,563 (1,241,571-2,604,865)	275 (100–594)	1,251,397 (797,765-1,673,743)	35 (11–86)	7,872,297 (2,392,999–11,897,903)	403 (135–884)	935,051 (596,095–1,250,631)	39 (15–91)	968,140 (645,427-1,261,516)	1,311 (520-2,921)	12,974,448 (5,077,762–18,688,658)	2,063 (766-4,576)
*Salmonella* spp.	34,649,374 (22,088,976–46,343,538)	184 (62–415)	54,761,300 (34,910,329–73,243,239)	16 (5–39)	123,833,314 (37,642,513–187,157,168)	463 (160-1,000)	14,092,509 (8,983,974–18,848,731)	69 (27–161)	33,961,555 (22,641,037–44,252,935)	458 (174-1,025)	261,298,052 (117,282,855–369,845,611)	1,190 (401-2,640)
*Shigella* spp.	9,270,018 (5,909,637–12,398,649)	3,662 (1,218-8,299)	8,149,719 (5,195,446)	303 (92–736)	36,375,499 (11,057,325–54,976,607)	9,602 (3,324–20,718)	4,279,020 (2,727,875-5,723,190)	1,465 (562-3,399)	5,737,751 (3,825,167-7,476,463)	8,318 (3,131–18,625)	63,812,007 (25,987,575–91,475,158)	23,350 (7,765–51,777)
Other	3,286,205 (2,094,955-4,395,299)	1,586 (533-3,574)	13,562,067 (8,645,817–18,139,264)	140 (43–339)	11,777,472 (3,580,084–17,800,044)	3,942 (1,362-8,516)	1,349,134 (860,073–1,804,467)	587 (225-1,361)	3,187,203 (2,124,802-4,153,022)	4,058 (1,545-9,077)	24,721,127 (11,064,550–35,002,320)	10,313 (3,483–22,867)
Unknown	26,417,334 (16,841,051–35,333,185)	9,588 (3,225–21,598)	25,195,705 (16,062,262–33,699,255)	848 (259-2,061)	102,670,888 (31,209,616–155,173,046)	23,714 (8,190–51,230)	12,041,324 (7,676,344–16,105,271)	3,516 (1,348-8,160)	17,273,711 (11,515,807–22,508,169)	24,776 (9,441–55,411)	183,598,962 (75,628,736–262,818,926)	62,442 (21,115–138,460)
Total	115,430,376 (73,586,866–154,399,127)	43,151 (14,497–97,269)	169,881,822 (108,299,659–227,216,933)	3,790 (1,156-9,206)	418,379,492 (127,177,857–632,323,553)	107,534 (37,150–232,279)	47,913,915 (30,545,119–64,084,862)	16,010 (6,139–37,152)	106,563,572 (71,042,381–138,855,565)	109,894 (41,821–245,807)	858,169,177 (380,106,763-1,216,869,040)	280,379 (94,624–621,713)

**Fig. 2:**
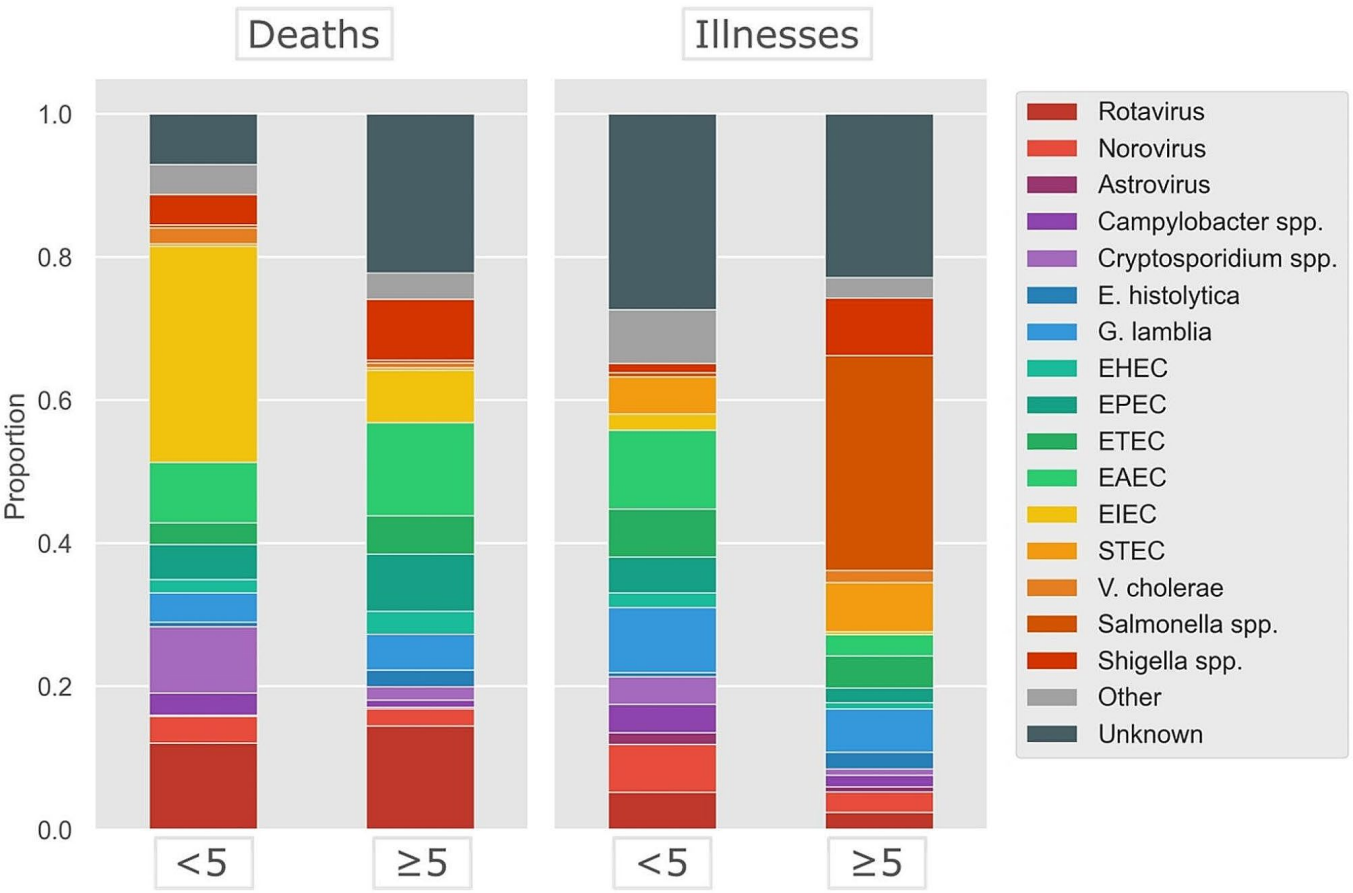
Relative contribution of 16 diarrheal agents, including other and unknown etiology, for deaths and illnesses in children below five and the population above five years of age. Proportion represents data from the whole African region, based on the results from the meta-analysis

Figure 2 shows the relative contribution of etiologies for diarrhea cases and deaths in children under five and the population above five in Africa. Of the 16 pathogens included in the SR, EAEC caused the largest number of diarrheal illnesses in children below five across the whole African region (17,137,586 cases, 95% UI 7,924,333 − 24,245,224), whereas *V. cholerae* caused the smallest (76,033 cases, 95% UI 43,923 − 103,420). Other pathogens causing a large number of diarrheal illnesses in children were *G. lamblia*, ETEC and norovirus (Table 1).

A large proportion of diarrheal cases in children under five was attributed to the *unknown* etiology (37,466,824 cases, 95% UI 15,244,051–53,990,266). Other pathogens causing a large number of diarrheal illnesses in children were *G. lamblia*, ETEC and norovirus (Table 1).

EIEC caused the largest number of diarrheal deaths in children under five (70,108 deaths, 95% UI 46,172 − 115,341), whereas astrovirus caused the smallest (457 deaths, 95% UI 299–750).

*Salmonella* spp. caused the largest number of diarrheal cases in the population > 5 across the whole African region (261,298,052 cases, 95% UI 117,282,855 − 369,845,611). EIEC caused the lowest number of diarrheal cases (3,816,254, 95% UI 1,734,617-5,392,165). The largest number of diarrheal deaths for the children above five and adults were from the *unknown* etiology (62,442 deaths, 95% UI 21,115–138,460), followed by rotavirus (39,586 deaths, 95% UI 13,077–87,779).

The estimates per 100,000 people (Tables 3 and 4) showed that the diarrheal incidence was highest for Eastern Africa in children below five (114,389 cases per 100,000 people, 95% UI 34,771 − 172,884). In the population above five, the incidence was highest in Central Africa (114,350 cases per 100,000 people, 95% UI 34,759 − 172,823). Diarrheal mortality was highest in Western Africa in all ages (children < 5: 194.5 deaths per 100,000 people, 95% UI 120-325.4; children ≥ 5 and above: 33.5 deaths per 100,000 people, 95% UI 12.9–75.1).

**Table 3:** Number of diarrheal illnesses and deaths per etiology per 100,000 people for children < 5 years of age in African regions, with 95% uncertainty interval (UI)

Pathogen	Northern Africa		Central Africa		Eastern Africa		Southern Africa		Western Africa		Whole African Region (197 mil. pop)	
	6 countries (29 mil. pop)		9 countries (30 mil. pop)		18 countries (67 mil. pop)		5 countries (7 mil. pop)		16 countries (64 mil. pop)			
	Illnesses (95% UI)	Deaths (95% UI)	Illnesses (95% UI)	Deaths (95% UI)	Illnesses (95% UI)	Deaths (95% UI)	Illnesses (95% UI)	Deaths (95% UI)	Illnesses (95% UI)	Deaths (95% UI)	Illnesses (95% UI)	Deaths (95% UI)
Rotavirus	4,334 (2,763-5,797)	19.5 (14.7–35.6)	8,924 (5,689–11,936)	3.5 (2.4–4.4)	4,662 (1,417-7,046)	9.2 (6.1–13.7)	2,824 (1,800-3,777)	7.1 (5.4–9.3)	3,415 (2,227-4,450)	22.2 (13.7–37.3)	24,159 (12,146–33,006)	61.5 (42.3-100.3)
Norovirus	5,603 (3,572-7,494)	6 (4.5–10.9)	5,543 (3,534-7.414)	1.1 (0.8–1.4)	7,633 (2,320–11,536)	2.8 (1.8–4.2)	5,313 (3,387-7,106)	2 (1.6–2.7)	2,319 (1,546-3,022)	7.9 (4.9–13.1)	26,411 (10,972–36,572)	19.8 (13.6–32.3)
Astrovirus	1,335 (851-1,785)	0.3 (0.2–0.5)	1,629 (1,039–2,179)	0.1 (0-0.1)	1,735 (527-2,622)	0.1 (0.1–0.2)	1,179 (752-1,577)	0.1 (0.1–0.1)	661 (440–861)	0.4 (0.2–0.7)	6,539 (2,857-9,024)	1 (0.6–1.6)
*Campylobacter* spp.	3,308 (2,109-4,424)	5 (3.7-9)	2,808 (1,790-3,756)	1 (0.7–1.2)	4,628 (1,407-6,994)	2.3 (1.4–3.4)	3,261 (2,079–4,362)	1.6 (1.2–2.1)	1,208 (805-1,574)	7.4 (4.6–12.3)	15,213 (6,111–21,110)	17.3 (11.6–28)
*Cryptosporidium* spp.	3,199 (2,040–4,279)	14.9 (11.2–27.1)	3,305 (2,107-4,420)	2.7 (1.9–3.5)	4,319 (1,313-6,527)	6.9 (4.5–10.4)	2,992 (1,907-4,001)	5.2 (4-6.8)	1,374 (916-1,790)	18.7 (11.5–31.2)	15,189 (6,376–21,017)	48.4 (33.1–79)
*Entamoeba histolytica*	459 (293–614)	1 (0.7–1.8)	585 (373–782)	0.2 (0.2–0.3)	590 (179–892)	0.4 (0.3–0.7)	398 (254–533)	0.3 (0.2–0.4)	236 (157–307)	1.8 (1.1–2.9)	2,268 (1,002–3,128)	3.7 (2.5–6.1)
*Giardia lamblia*	7,576 (4,830–10,133)	6.6 (4.9–12)	8,014 (5,109–10,719)	1.2 (0.8–1.5)	10,176 (3,093–15,379)	3.1 (2.1–4.6)	7,031 (4,483-9,405)	2.4 (1.8–3.1)	3,319 (2,213-4,325)	7.3 (4.5–12.3)	36,116 (15,245–49,961)	20.6 (14.1–33.5)
EHEC	1,671 (1,065–2,235)	3 (2.3–5.5)	1,243 (793-1,663)	0.6 (0.4–0.7)	2,384 (725-3,604)	1.4 (0.9–2.1)	1,697 (1,082–2,270)	0.9 (0.7–1.2)	543 (362–708)	4.7 (2.9–7.8)	7,538 (2,945–10,480)	10.6 (7.2–17.3)
EPEC	4,157 (2,650-5,560)	7.8 (5.9–14.2)	3,838 (2,446-5,133)	1.5 (1.1–1.9)	5,733 (1,743-8,665)	3.6 (2.3–5.4)	4,013 (2,558-5,367)	2.6 (2-3.4)	1,625 (1,084–2,118)	10.9 (6.8–18.2)	19,336 (7,923–26,843)	26.4 (18.1–43.1)
ETEC	5,589 (3,563-7,475)	4.9 (3.7–8.9)	4,966 (3,166-6,643)	0.9 (0.6–1.1)	7,760 (2,359–11,279)	2.3 (1.5–3.4)	5,449 (3,474-7,288)	1.8 (1.3–2.3)	2,118 (1,412-2,760)	5.7 (3.5–9.5)	25,882 (10,500–35,895)	15.6 (10.6–25.2)
EAEC	9,164 (5,842–12,257)	13.7 (10.2–24.8)	10,173 (6,485–13,607)	2.6 (1.9–3.3)	12,181 (3,703–18,410)	6.3 (4-9.4)	8,373 (5,338–11,199)	4.5 (3.5–5.9)	4,182 (2,788-5,449)	19 (11.8–31.7)	44,073 (18,818–60,922)	46.1 (31.4–75.1)
EIEC	1,865 (1,189-2,494)	48.6 (36.5–88.6)	3,513 (2,239-4,698)	8.7 (6.2–11.2)	2,094 (636-3,165)	22.7 (15-34.1)	1,304 (831-1,744)	17.4 (13.3–22.7)	1,355 (903-1,766)	57.6 (35.5–96.3)	10,131 (4,967–13,867)	155 (106.5-252.9)
STEC	4,308 (2,747-5,762)	0.6 (0.4-1)	6,036 (3,848-8,074)	0.1 (0.1–0.1)	5,391 (1,639-8,148)	0.3 (0.2–0.4)	3,590 (2,289-4,801)	0.2 (0.2–0.3)	2,404 (1,603-3,133)	0.4 (0.2–0.7)	21,729 (9,837–29,918)	1.6 (1.1–2.5)
*V. cholerae*	29 (19–39)	3.5 (2.7–6.6)	97 (62–130)	0.5 (0.4–0.7)	22 (7–33)	1.8 (1.2–2.6)	9 (6–12)	1.5 (1.1-2)	36 (24–47)	2.3 (1.4-4)	193 (112–261)	9.6 (6.8–15.9)
*Salmonella* spp.	460 (293–616)	0.7 (0.5–1.2)	596 (380–797)	0.1 (0.1–0.1)	590 (179–891)	0.3 (0.2–0.5)	397 (253–531)	0.3 (0.2–0.4)	240 (160–313)	0.4 (0.2–0.7)	2,283 (1,012–3,148)	1.8 (1.2–2.9)
*Shigella* spp.	1,066 (680-1,426)	6.9 (5.1–12.5)	1,356 (864-1,813)	1.3 (0.9–1.6)	1,371 (417-2,072)	3.2 (2-4.8)	929 (590-1,239)	2.3 (1.8-3)	547 (364–712)	9.2 (5.7–15.4)	5,266 (2,325-7,262)	22.9 (15.5–37.3)
Other	6,240 (3,978-8,346)	6.8 (5.1–12.4)	4,984 (3,177-6,666)	1.2 (0.9–1.6)	8,808 (2,677–13,312)	3.2 (2.1–4.8)	6,237 (3,976-8,342)	2.4 (1.9–3.2)	2,168 (1,446-2,825)	7.9 (4.9–13.3)	28,437 (11,278–39,491)	21.5 (14.9–35.3)
Unknown	22,753 (14,505–30,432)	11.4 (8.6–20.8)	9,885 (6,302–13,221)	1.9 (1.3–2.5)	34,312 (10,430–51,859)	5.4 (3.6-8)	25,005 (15,941–33,444)	4.4 (3.3–5.7)	5,014 (3,342-6,533)	10.7 (6.6–18)	96,969 (34,579–135,489)	33.8 (23.4–55)
Total	83,116 (52,989–111,168)	161.2 (120.9-293.4)	77,495 (49,403–103,651)	29.2 (20.7–37.2)	114,389 (34,771–172,884)	75.3 (49.3-112.7)	79,998 (51,000-106,998)	57 (43.6–74.6)	32,764 (21,842–42,693)	194.5 (120-325.4)	387,762 (159,005-537,394)	517.2 (354.5-843.3)

**Table 4:** Number of diarrheal illnesses and deaths per etiology per 100,000 people in children ≥ 5 years of age and adults in African regions, with 95% uncertainty interval (UI)

Pathogen	Northern Africa		Central Africa		Eastern Africa		Southern Africa		Western Africa		Whole African Region (1,1 bil. pop)	
	6 countries (212 mil. pop)		9 countries (144 mil. pop)		18 countries (366 mil. pop)		5 countries (60 mil. pop)		16 countries (327 mil. pop)			
	Illnesses (95% UI)	Deaths (95% UI)	Illnesses (95% UI)	Deaths (95% UI)	Illnesses (95% UI)	Deaths (95% UI)	Illnesses (95% UI)	Deaths (95% UI)	Illnesses (95% UI)	Deaths (95% UI)	Illnesses (95% UI)	Deaths (95% UI)
Rotavirus	1,323 (844-1,770)	3 (1-6.7)	7,295 (4,651-9,758)	0.4 (0.1–0.9)	1,908 (580-2,884)	4.6 (1.6–9.9)	1,110 (708-1,485)	4.3 (1.7–10)	1,798 (1,198-2,342)	4.1 (1.5–9.2)	13,434 (7,273–18,239)	16.4 (4.2–36.7)
Norovirus	1,547 (986-2,069)	0.5 (0.2–1.1)	4,257 (2,714-5,693)	0.1 (0-0.2)	3,064 (931-4,630)	0.7 (0.2–1.5)	2,112 (1,346-2,824)	0.6 (0.2–1.5)	1,133 (755-1,477)	0.8 (0.3–1.8)	12,113 (5,386–16,693)	2.7 (0.7–6.1)
Astrovirus	374 (239–500)	0 (0-0.1)	1,279 (815-1,710)	0 (0–0)	698 (212-1,055)	0 (0-0.1)	462 (295–618)	0 (0–0)	331 (221–431)	0.1 (0-0.3)	3,144 (1,487-)	0.1 (0-0.5)
*Campylobacter* spp.	895 (571-1,197)	0.2 (0.1–0.5)	2,111 (1,346-2,823)	0 (0-0.1)	1,851 (563-2,798)	0.3 (0.1–0.7)	1,292 (824-1,728)	0.3 (0.1–0.8)	575 (383–749)	0.2 (0.1–0.5)	6,724 (2,863-9,295)	1 (0.3–2.6)
*Cryptosporidium* spp.	480 (306–642)	0.4 (0.1–0.9)	1,692 (1,079–2,264)	0.1 (0-0.1)	872 (265-1,318)	0.5 (0.2–1.2)	572 (364–765)	0.5 (0.2–1.1)	435 (290–566)	0.7 (0.2–1.5)	4,051 (1,940-5,555)	2.2 (0.5–4.8)
*Entamoeba histolytica*	1,268 (808-1,696)	0.5 (0.2–1.1)	5,360 (3,417-7,169)	0.1 (0-0.2)	2,152 (654-3,252)	0.7 (0.2–1.5)	1,373 (875-1,836)	0.6 (0.2–1.4)	1,353 (902-1,763)	0.8 (0.3–1.8)	11,506 (5,781–15,716)	2.7 (0.7-6)
*Giardia lamblia*	3,306 (2,108-4,422)	1 (0.3–2.3)	9,406 (5,996–12,580)	0.1 (0-0.3)	6,486 (1,972-9,803)	1.4 (0.5–3.1)	4,446 (2,834-5,946)	1.3 (0.5-3)	2.487 (1.658–3.241)	1.7 (0.7–3.9)	26,131 (11,734–35,992)	5.5 (1.5–12.6)
EHEC	484 (308–647)	0.6 (0.2–1.4)	1,084 (691-1,450)	0.1 (0-0.3)	1,010 (307-1,526)	0.6 (0.2–1.2)	705 (449–942)	0.3 (0.1–0.8)	298 (199–388)	2 (0.8–4.4)	3,581 (1,505-4,953)	3.6 (1.2–8.1)
EPEC	1,086 (692-1,452)	1.6 (0.5–3.7)	2,586 (1,649-3,459)	0.2 (0.1–0.4)	2,235 (679-3,378)	2.7 (0.9–5.8)	1,550 (988-2,074)	2.6 (1-6.1)	703 (469–916)	1.8 (0.7–4.1)	8,160 (3,489–11,279)	8.9 (2.2–20.1)
ETEC	2,454 (1,565-3,283)	1.1 (0.4–2.5)	6,046 (3,854-8,087)	0.2 (0-0.4)	5,002 (1,521-7,560)	1.5 (0.5–3.2)	3,488 (2,223-4,665)	1.3 (0.5-3)	1,637 (1,092–2,133)	2.1 (0.8–4.6)	18,627 (8,032–25,728)	6.2 (1.7–13.7)
EAEC	1,618 (1,031–2,163)	2.6 (0.9-6)	4,431 (2,825-5,926)	0.3 (0.1–0.8)	3,194 (971-4,827)	4 (1.4–8.6)	2,167 (1,382-2,899)	3.7 (1.4–8.6)	1,181 (787-1,539)	4 (1.5–8.9)	12,591 (5,614–17,354)	14.6 (3.9–32.9)
EIEC	235 (150–315)	1.5 (0.5–3.3)	585 (373–782)	0.2 (0.1–0.5)	481 (146–726)	1.8 (0.6–3.9)	332 (212–445)	1.5 (0.6–3.4)	158 (105–206)	3.3 (1.3–7.3)	1,791 (774-2,474)	8.3 (2.5–18.4)
STEC	3,723 (2,373-4,979)	0.1 (0-0.2)	6,163 (3,929-8,243)	0 (0–0)	8,117 (2,486–12,359)	0.1 (0-0.3)	5,821 (3,711-7,785)	0.1 (0-0.3)	1,796 (1,197-2,340)	0.1 (0-0.2)	25,680 (9,985–35,706)	0.4 (0–1)
*V. cholerae*	918 (585-1,228)	0.1 (0-0.3)	868 (553-1,161)	0 (0-0.1)	2,152 (654-3,252)	0.1 (0-0.2)	1,563 (996-2,090)	0.1 (0-0.2)	296 (197–386)	0.4 (0.2–0.9)	5,797 (1,989-8,117)	0.7 (0.2–1.7)
*Salmonella* spp.	16,336 (10,414–21,850)	0.1 (0-0.2)	37,979 (24,212–50,797)	0 (0–0)	33,846 (10,288–51,153)	0.1 (0-0.3)	23,550 (15,013–31,498)	0.1 (0-0.3)	10,379 (6,919–13,524)	0.1 (0.1–0.3)	122,090 (51,833–168,822)	0.4 (0.1–1.1)
*Shigella* spp.	4,371 (2,786-5,846)	1.7 (0.6–3.9)	5,652 (3,603-7,560)	0.2 (0.1–0.5)	9,942 (3,022–15,026)	2.6 (0.9–5.7)	7,151 (4,559-9,564)	2.4 (0.9–5.7)	1,753 (1,169-2,285)	2.5 (1-5.7)	28,869 (10,580–40,281)	9.4 (2.6–21.5)
Other	1,549 (988-2,072)	0.7 (0.3–1.7)	3,552 (2,264-4,750)	0.1 (0-0.2)	3,219 (978-4,865)	1.1 (0.4–2.3)	2,225 (1,437)	1 (0.4–2.3)	947 (649-1,269)	1.2 (0.5–2.8)	11,549 (4,879–15,971)	4.1 (1.2–9.3)
Unknown	12,455 (7,940–16,659)	4.5 (1.5–10.2)	17,474 (11,140–23,372)	0.6 (0.2–1.4)	28,061 (8,530–42,411)	6.5 (2.2–14)	20,122 (12,828–26,913)	5.9 (2.3–13.6)	5,279 (3,519-6,879)	7.6 (2.9–16.9)	83,391 (31,129–116,234)	25.1 (6.8–56.1)
Total	54,422 (34,694–72,790)	20.2 (6.8–46.1)	117,820 (75,111–157,584)	2.7 (0.7–6.4)	114,350 (34,759–172,823)	29.3 (9.9–63.5)	80,071 (51,044–107,092)	26.6 (10.1–62.1)	32,566 (21,709–42,434)	33.5 (12.9–75.1)	399,229 (166,273–552,723)	112,3 (30,3-253,2)

### Sensitivity analysis

Replacing the diarrhea incidence estimates with those from GBD 2019 (Supplementary Material Table S11) increased the median incidence of diarrheal cases (Tables 5, 6, 7 and 8), especially for pathogens with large uncertainty intervals due to limited data. However, the 95% UIs showed considerable overlap, suggesting that using more recent incidence estimates from four African countries was more conservative, but in the range of GBD2019. Replacing the diarrheal mortality estimates with those from GBD 2019 (Supplementary Material Table S11) had less impact; the median estimates for diarrheal deaths showed less variation between the two data sets, with the death estimates from the GBD data slightly higher than our data. The GBD estimated that Western Africa had the highest diarrheal incidence in children < 5 (129,889,538 illnesses, 95% UI 105,361,416 − 154,560,873). This is in contrast to our estimates, which showed that Eastern Africa had the highest incidence under five (76,493,449 illnesses, 95% UI 23,252,270 − 115,609,415). In the population above five, the GBD 2019 estimated that Eastern Africa had the highest diarrheal incidence and mortality (253,335,421 illnesses, 95% UI 221,175,475 − 287,872,646 and 83,652 deaths, 95% UI 41,206 − 145,029).

**Table 5:** Total estimates of diarrheal cases and deaths by etiology in children < 5 years of age in African regions, with 95% uncertainty interval (UI), using the Global Burden of Disease incidence and mortality envelopes, 2019. Country-specific data were extracted from https://vizhub.healthdata.org/gbd-compare/

Pathogen	Northern Africa		Central Africa		Eastern Africa		Southern Africa		Western Africa		Whole African Region (197 mil. pop)	
	6 countries (29 mil. pop)		9 countries (30 mil. pop)		18 countries (67 mil. pop)		5 countries (7 mil. pop)		16 countries (64 mil. pop)			
	Illnesses (95% UI)	Deaths (95% UI)	Illnesses (95% UI)	Deaths (95% UI)	Illnesses (95% UI)	Deaths (95% UI)	Illnesses (95% UI)	Deaths (95% UI)	Illnesses (95% UI)	Deaths (95% UI)	Illnesses (95% UI)	Deaths (95% UI)
Rotavirus	5,817,227 (4,759,468-6,938,793)	1,670 (767-2,990)	3,892,143 (3,261,623-4,459,577)	8,413 (3,811–14,759)	5,423,424 (4,376,222-6,524,706)	10,073 (5,757–16,117)	205,346 (162,996–254,434)	570 (390–809)	13,755,827 (11,078,579–16,474,203)	21,821 (14,323–31,829)	29,093,967 (23,475,892–34,651,713)	42,547 (25,048–66,504)
Norovirus	3,858,062 (3,179,826-4,550,898)	567 (266–999)	4,560,375 (3,736,057–5,309,153)	2,752 (1,289-4,754)	8,263,368 (6,690,371-9,929,653)	3,011 (1,718-4,819)	385,974 (306,374–478,237)	165 (113–234)	9,208,111 (7,464,199–10,963,836)	7,727 (5,176–11,125)	26,275,890 (21,070,453–31,231,777)	14,222 (8,562–21,931)
Astrovirus	1,107,975 (910,854-1,312,079)	28 (13–50)	1,111,217 (915,221-1,288,899)	137 (65–236)	1,903,830 (1,540,439-2,288,232)	146 (83–233)	85,735 (68,035–106,230)	8 (5–11)	2,635,530 (2,131,578-3,144,431)	394 (266–566)	6,844,287 (5,498,092–8,139,871)	713 (432-1,096)
*Campylobacter* spp.	1,995,368 (1,648,072–2,346,062)	514 (246–894)	2,657,902 (2,170,188-3,101,453)	2,419 (1,166-4,121)	4,974,216 (4,028,831-5,976,449)	2,425 (1,381-3,881)	237,156 (188,245–293,848)	128 (87–181)	4,772,423 (3,875,740-5,672,895)	7,252 (4,934–10,335)	14,637,065 (11,722,831–17,390,707)	12,738 (7,814–19,412)
*Cryptosporidium* spp.	2,289,264 (1,885,742-2,702,732)	1,360 (634-2,410)	2,616,489 (2,145,754-3,043,927)	6,691 (3,097–11,620)	4,688,288 (3,795,400-5,633,868)	7,562 (4,317–12,099)	217,555 (172,686–269,561)	419 (287–594)	5,458,762 (4,422,713-6,502,534)	18,285 (12,167–26,439)	15,270,358 (12,249,609–18,152,622)	34,317 (20,502–53,162)
*Entamoeba histolytica*	396,004 (325,398–469,288)	117 (57–201)	384,036 (316,676–445,074)	531 (265–888)	649,478 (525,430–780,655)	468 (266–749)	28,998 (23,017–35,930)	23 (16–33)	941,264 (760,964-1,123,436)	1,724 (1,193-2,428)	2,399,780 (1,928,468-2,854,383)	2,863 (1,797-4,299)
*Giardia lamblia*	5,536,115 (4,558,901-6,539,015)	554 (254–995)	6,209,708 (5,095,427-7,221,290)	2,807 (1,265-4,936)	11,061,913 (8,954,563–13,293,302)	3,404 (1,946-5,447)	511,353 (405,892–633,592)	193 (132–275)	13,191,958 (10,685,394–15,718,104)	7,192 (4,705–10,513)	36,511,047 (29,294,285–43,405,303)	14,150 (8,302–22,166)
EHEC	899,302 (744,396-1,053,891)	324 (156–560)	1,326,085 (1,079,720-1,550,366)	1,518 (742-2,568)	2,547,293 (2,063,773-3,060,218)	1,459 (830-2,335)	123,429 (97,973–152,935)	75 (51–106)	2,145,459 (1,745,610-2,545,949)	4,743 (3,251-6,725)	7,041,568 (5,633,499-8,363,359)	8,119 (5,030–12,294)
EPEC	2,695,635 (2,223,802-3,175,226)	772 (365-1,351)	3,363,686 (2,751,373-3,920,203)	3,694 (1,754-6,339)	6,186,604 (5,009,826-7,433,616)	3,882 (2,213-6,212)	291,832 (231,645–361,595)	209 (143–296)	6,439,830 (5,224,400-7,662,150)	10,695 (7,218–15,322)	18,977,587 (15,209,401–22,552,790)	19,252 (11,693–29,520)
ETEC	3,506,464 (2,894,265-4,126,901)	424 (195–758)	4,507,818 (3,684,161-5,256,642)	2,125 (967-3,721)	8,358,712 (6,769,386–10,043,231)	2,519 (1,440-4,031)	396,271 (314,544–490,999)	142 (97–201)	8,382,117 (6,803,289-9,968,875)	5,571 (3,666-8,112)	25,151,382 (20,151,101–29,886,649)	10,781 (6,365–16,823)
EAEC	6,989,259 (5,752,135-8,262,872)	1,345 (637-2,356)	7,550,092 (6,202,835-8,772,623)	6,441 (3,057–11,054)	13,281,647 (10,749,873–15,961,604)	6,777 (3,863–10,845)	608,950 (483,360–754,519)	365 (250–518)	16,647,429 (13,477,258–19,844,599)	18,642 (12,580–26,710)	45,077,377 (36,182,101–53,596,217)	33,570 (20,387–51,483)
EIEC	2,304,669 (1,886,977-2,746,020)	4,269 (1,973-7,610)	1,652,640 (1,380,127-1,898,262)	21,296 (9,732–37,206)	2,406,034 (1,942,661-2,894,034)	24,914 (14,234–39,864)	95,502 (75,804–118,336)	1,398 (957-1,984)	5,448,735 (4,390,860-6,522,043)	56,443 (37,262–82,029)	11,907,580 (9,600,625–14,178,695)	108,320 (64,158–168,693)
STEC	4,051,548 (3,325,846-4,808,599)	35 (15–66)	3,647,319 (3,015,998-4,218,757)	200 (80–368)	5,982,253 (4,837,825-7,191,464)	307 (176–491)	261,071 (207,228–323,479)	19 (13–27)	9,619,865 (7,770,351–11,490,718)	379 (224–587)	23,562,056 (18,950,020–28,033,017)	940 (508-1,539)
*V. cholerae*	61,796 (50,417–74,023)	217 (90–414)	29,187 (24,984–32,927)	1,261 (502-2,332)	28,985 (23,250–34,943)	1,970 (1,131-3,151)	649 (515–804)	121 (83–171)	145,623 (116,985–174,792)	2,300 (1,339-3,594)	266,240 (215,636–317,489)	5,869 (3,145-9,662)
*Salmonella* spp.	403,086 (331,163–477,798)	38 (15–74)	386,397 (318,762–447,677)	225 (87–421)	650,297 (526,061–781,660)	371 (213–594)	28,872 (22,918–35,774)	23 (15–32)	957,976 (774,353-1,143,546)	373 (207–597)	2,426,628 (1,950,339-2,886,455)	1,030 (537-1,718)
*Shigella* spp.	918,181 (754,483-1,088,076)	660 (311-1,160)	891,454 (735,068–1,033,165)	3,187 (1500-5,492)	1,509,117 (1,220,887-1,813,930)	3,438 (1,961-5,502)	67,403 (53,502–83,516)	187 (128–265)	2,183,378 (1,765,180-2,605,908)	9,050 (6,079–13,005)	5,569,533 (4,475,618-6,624,595)	16,522 (9,979–25,424)
Other	3,572,303 (2,953,234-4,194,252)	592 (273-1,056)	4,986,473 (4,066,458-5,823,565)	2,961 (1,349-5,179)	9,445,123 (7,651,000–11,347,675)	3,490 (1,994-5,584)	453,435 (359,918–561,829)	196 (134–279)	8,554,308 (6,952,520–10,161,094)	7,789 (5,132–11,333)	27,011,642 (21,623,212–32,088,415)	15,028 (8,882–23,431)
Unknown	7,964,199 (6,659,646-9,185,480)	857 (382-1,568)	17,519,591 (14,154,762–20,590,392)	4,502 (1,953-8,046)	36,128,418 (29,291,357–43,392,607)	6,004 (3,438-9,607)	1,819,206 (1,444,006–2,254,100)	349 (239–496)	19,400,943 (15,921,443–22,841,760)	10,495 (6,672–15,611)	82,832,357 (66,027,208–98,264,339)	22,207 (12,684–35,328)
Total	54,366,457 (44,844,625–64,052,006)	14,343 (6,649–25,512)	67,292,612 (55,055,194–78,413,952)	71,160 (32,681–124,040)	123,489,000 (99,997,155–148,381,847)	82,220 (46,961–131,561)	5,818,737 (4,618,676-7,209,718)	4,590 (3,140-6,512)	129,889,538 (105,361,416–154,560,873)	190,875 (126,394–276,860)	380,856,344 (305,258,390–452,618,396)	363,188 (215,825–564,485)

**Table 6:** Total estimates of diarrheal cases and deaths by etiology in children ≥ 5 years of age and adults in African regions, with 95% uncertainty interval (UI), using the Global Burden of Disease incidence and mortality envelopes, 2019. Country-specific data were extracted from https://vizhub.healthdata.org/gbd-compare/

Pathogen	Northern Africa		Central Africa		Eastern Africa		Southern Africa		Western Africa		Whole African Region (1,1 bil. pop)	
	6 countries (212 mil. pop)		9 countries (144 mil. pop)		18 countries (366 mil. pop)		5 countries (60 mil. pop)		16 countries (327 mil. pop)			
	Illnesses (95% UI)	Deaths (95% UI)	Illnesses (95% UI)	Deaths (95% UI)	Illnesses (95% UI)	Deaths (95% UI)	Illnesses (95% UI)	Deaths (95% UI)	Illnesses (95% UI)	Deaths (95% UI)	Illnesses (95% UI)	Deaths (95% UI)
Rotavirus	8,058,376 (6,867,097–9,357,334)	358 (152–692)	2,809,264 (2,447,773-3,194,581)	4,489 (2,259-8,206)	4,558,823 (3,961,251-5,196,875)	13,058 (6,449–22,588)	647,904 (572,565–730,139)	1,866 (1,019–3,517)	13,637,126 (11,978,723–15,394,029)	9,305 (4,801–16,977)	29,711,493 (25,254,844–33,872,958)	29,076 (13,661–51,980)
Norovirus	4,748,832 (4,046,404-5,517,238)	64 (28–124)	3,102,604 (2,711,604-3,520,001)	743 (378-1,351)	6,852,288 (5,978,371-7,790,261)	1,975 (972-3,425)	1,205,006 (1,064,856-1,358,003)	272 (149–513)	8,655,019 (7,589,325-9,788,965)	1,888 (981-3,454)	24,563,749 (20,325,704–27,974,468)	4,942 (2,359-8,867)
Astrovirus	1,423,435 (1,212,909-1,653,459)	7 (3–14)	760,570 (664,215–863,386)	61 (32–107)	1,579,526 (1,377,048–1,796,559)	91 (44–162)	270,762 (239,279–305,129)	8 (4–16)	2,525,984 (2,216,319-2,854,967)	289 (152–531)	6,560,277 (5,470,491-7,473,500)	456 (231–830)
*Campylobacter* spp.	2,364,737 (2,014,880-2,747,863)	24 (10–45)	1,780,968 (1,557,281-2,019,798)	318 (159–584)	4,106,789 (3,584,745-4,667,263)	992 (491-1,713)	744,938 (658,309–839,504)	146 (80–275)	4,406,253 (3,861,668-4,986,476)	529 (271–961)	13,403,685 (11,018,574–15,260,904)	2,009 (931-3,578)
*Cryptosporidium* spp.	1,875,944 (1,598,516-2,178,997)	50 (22–97)	978,028 (854,010–1,110,358)	580 (295-1,053)	1,974,124 (1,720,648-2,245,839)	1,534 (755-2,660)	333,152 (294,404–375,455)	211 (115–397)	3,313,486 (2,907,661-3,744,518)	1,494 (776-2,732)	8,474,734 (7,080,835-9,655,167)	3,869 (1,848-6,939)
*Entamoeba histolytica*	5,938,681 (5,060,613-6,897,052)	62 (27–120)	2,622,078 (2,287,823-2,978,596)	722 (367-1,311)	4,958,463 (4,317,922-5,644,160)	1,914 (942-3,320)	796,949 (704,278–898,108)	263 (144–496)	10,290,160 (9,033,768–11,623,084)	1,846 (959-3,377)	24,606,331 (20,700,126–28,041,000)	4,807 (2,295-8,624)
*Giardia lamblia*	10,476,354 (8,926,814–12,171,034)	134 (58–259)	6,641,618 (5,804,007–7,535,717)	1,556 (791-2,826)	14,526,416 (12,672,778–16,515,256)	4,117 (2,027–7,141)	2,577,952 (2,278,196-2,905,152)	566 (309-1,067)	19,013,808 (16,674,341–21,502,492)	3,984 (2,069–7,288)	53,236,148 (44,077,940–60,629,651)	10,357 (4,945–18,581)
EHEC	1,215,175 (1,035,379-1,412,139)	115 (53–224)	959,434 (839,036–1,087,995)	1,003 (533-1,771)	2,239,464 (1,955,023–2,544,896)	1,549 (742-2,747)	409,049 (361,484–460,970)	144 (79–271)	2,287,629 (2,004,538-2,589,378)	4,703 (2,472-8,643)	7,110,751 (5,833,976-8,095,378)	7,514 (3,800–13,656)
EPEC	2,891,787 (2,463,950-3,360,267)	182 (75–351)	2,160,096 (1,888,701-2,449,875)	2,468 (1,230-4,538)	4,964,741 (4,333,506-5,642,407)	7,749 (3,836–13,376)	897,026 (792,698-1,010,917)	1,140 (622-2,148)	5,395,766 (4,729,033–6,106,056)	4,017 (2,054–7,303)	16,309,416 (13,415,190–18,569,522)	15,556 (7,195–27,716)
ETEC	6,772,457 (5,770,560-7,869,260)	151 (66–293)	4,894,399 (4,279,086–5,551,351)	1,676 (857-3,032)	11,135,510 (9,718,833–12,656,195)	4,184 (2,055–7,271)	2,027,098 (1,791,409-2,284,346)	560 (306-1,055)	12,563,362 (11,012,281–14,215,332)	4,786 (2,492-8,763)	37,392,826 (30,780,760–42,576,484)	11,357 (5,470–20,414)
EAEC	4,955,936 (4,222,742-5,758,032)	330 (141–639)	3,240,116 (2,831,786-3,675,992)	4,029 (2,035–7,348)	7,143,300 (6,232,425-8,120,490)	11,375 (5,613–19,695)	1,266,516 (1,119,237-1,427,280)	1,608 (879-3,032)	9,029,701 (7,917,956–10,212,656)	9,025 (4,668–16,482)	25,635,569 (21,204,909–29,194,450)	26,367 (12,457–47,196)
EIEC	653,824 (557,099–759,706)	221 (99–429)	469,605 (410,559–532,646)	2,281 (1,179-4,100)	1,070,420 (934,217-1,216,626)	5,100 (2,493-8,897)	193,029 (170,583–217,531)	643 (351-1,212)	1,210,090 (1,060,724-1,369,161)	7,650 (3,998–14,027)	3,596,968 (2,962,599-4,095,670)	15,895 (7,769–28,665)
STEC	6,961,661 (5,931,026–8,093,808)	9 (4–17)	7,292,863 (6,382,210-8,265,606)	116 (58–213)	17,981,934 (15,707,003–20,426,958)	359 (178–620)	3,384,184 (2,990,666-3,813,743)	53 (29–99)	13,861,378 (12,131,341–15,710,901)	197 (101–359)	49,482,020 (40,151,580–56,311,016)	734 (341-1,308)
*V. cholerae*	1,005,540 (856,471-1,170,516)	23 (11–45)	1,771,593 (1,551,745-2,006,535)	202 (107–356)	4,684,846 (4,094,897-5,319,335)	310 (149–550)	907,117 (801,635-1,022,259)	29 (16–55)	2,313,976 (2,019,583-2,630,742)	948 (498-1,743)	10,683,072 (8,522,696–12,149,387)	1,512 (765-2,749)
*Salmonella* spp.	42,531,054 (36,238,528–49,422,512)	11 (5–22)	32,471,802 (28,394,173–36,825,686)	132 (67–241)	75,170,995 (65,617,544–85,428,360)	360 (177–624)	13,671,551 (12,081,782–15,406,909)	50 (27–95)	79,589,023 (69,749,115–90,074,127)	320 (166–584)	243,434,425 (199,999,360–277,157,594)	873 (415-1,566)
*Shigella* spp.	6,449,203 (5,493,974-7,501,467)	214 (91–414)	8,496,768 (7,439,113-9,626,790)	2,628 (1,326-4,797)	21,746,974 (19,002,541–24,697,433)	7,476 (3,690–12,941)	4,151,226 (3,668,509-4,678,149)	1,060 (579-1,998)	13,607,012 (11,895,160–15,442,039)	5,771 (2,983–10,538)	54,451,183 (43,830,788–61,945,878)	17,149 (8,090–30,688)
Other	3,987,844 (3,397,846-4,634,058)	97 (42–188)	3,077,555 (2,691,201-3,490,078)	1,141 (579-2,074)	7,149,319 (6,240,926-8,124,681)	3,067 (1,511-5,318)	1,307,211 (1,155,213-1,473,124)	425 (232–800)	7,471,215 (6,547,201-8,455,945)	2,842 (1,475-5,196)	22,993,144 (18,877,174–26,177,886)	7,572 (3,607–13,576)
Unknown	19,862,476 (16,921,158–23,099,532)	591 (255-1,143)	24,272,864 (21,248,551–27,503,920)	6,900 (3,505–12,540)	61,491,489 (53,725,797–69,839,052)	18,442 (9,082–31,981)	11,681,956 (10,323,544–13,164,769)	2,546 (1,390-4,799)	40,939,856 (35,802,475–46,442,100)	17,364 (9,013–31,754)	158,248,641 (127,697,981–180,049,373)	45,843 (21,855–82,217)
Total	132,173,316 (112,615,966–153,604,274)	2,643 (1,142-5,116)	107,802,225 (94,282,874–122,238,911)	31,045 (15,757–56,448)	253,335,421 (221,175,475–287,872,646)	83,652 (41,206–145,029)	46,472,626 (41,068,647–52,371,487)	11,590 (6,330–21,845)	250,110,844 (219,131,212–283,142,968)	76,958 (39,929–140,712)	789,894,432 (647,205,527–899,230,286)	205,888 (98,034–369,150)

**Table 7:** Number of diarrheal illnesses and cases per 100,000 people for children < 5 years of age in African regions, with 95% uncertainty interval (UI), using the Global Burden of Disease incidence and mortality envelopes, 2019. Country-specific data were extracted from https://vizhub.healthdata.org/gbd-compare/

Pathogen	Northern Africa		Central Africa		Eastern Africa		Southern Africa		Western Africa		Whole African Region (197 mil. pop)	
	6 countries (29 mil. pop)		9 countries (30 mil. pop)		18 countries (67 mil. pop)		5 countries (7 mil. pop)		16 countries (64 mil. pop)			
	Illnesses (95% UI)	Deaths (95% UI)	Illnesses (95% UI)	Deaths (95% UI)	Illnesses (95% UI)	Deaths (95% UI)	Illnesses (95% UI)	Deaths (95% UI)	Illnesses (95% UI)	Deaths (95% UI)	Illnesses (95% UI)	Deaths (95% UI)
Rotavirus	19,992 (16,357–23,847)	5.7 (2.6–10.3)	12,892 (10,804–14,772)	27.9 (12.6–48.9)	8,110 (6,544-9,757)	15.1 (8.6–24.1)	3,025 (2,401-3,748)	8.4 (5.7–11.9)	21,423 (17,253–25,656)	34 (22.3–49.6)	65,442 (50,958–77,780)	91.1 (51.8-144.8)
Norovirus	13,259 (10,928–15,640)	1.9 (0.9–3.4)	15,105 (12,375–17,586)	9.1 (4.3–15.7)	12,357 (10,005–14,849)	4.5 (2.6–7.2)	5,686 (4,513-7,045)	2.4 (1.7–3.4)	14,340 (11,624–17,075)	12 (8.1–17.3)	60,747 (44,932–72,195)	29.9 (17.6–47)
Astrovirus	3,808 (3,130-4,509)	0.1 (0-0.2)	3,681 (3,032–4,269)	0.5 (0.2–0.8)	2,847 (2,304-3,422)	0.2 (0.1–0.3)	1,263 (1,002–1,565)	0.1 (0.1–0.2)	4,104 (3,320-4,897)	0.6 (0.4–0.9)	15,703 (11,786–18,662)	1.5 (0.8–2.4)
*Campylobacter* spp.	6,858 (5,664-8,063)	1.8 (0.8–3.1)	8,804 (7,188–10,273)	8 (3.9–13.6)	7,438 (6,025–8,937)	3.6 (2.1–5.8)	3,493 (2,773-4,329)	1.9 (1.3–2.7)	7,432 (6,036–8,835)	11.3 (7.7–16.1)	34,025 (24,913–40,437)	26.6 (15.8–41.3)
*Cryptosporidium* spp.	7,868 (6,481-9,289)	4.7 (2.2–8.3)	8,667 (7,107–10,082)	22.2 (10.3–38.5)	7,011 (5,676-8,425)	11.3 (6.5–18.1)	3,205 (2,544-3,971)	6.2 (4.2–8.8)	8,501 (6,888–10,127)	28.5 (18.9–41.2)	35,252 (26,152–41,894)	72.9 (42.1-114.9)
*Entamoeba histolytica*	1,361 (1,118-1,613)	0.4 (0.2–0.7)	1,272 (1,049–1,474)	1.8 (0.9–2.9)	971 (786-1,167)	0.7 (0.4–1.1)	427 (339–529)	0.3 (0.2–0.5)	1,466 (1,185-1,750)	2.7 (1.9–3.8)	5,497 (4,138-6,533)	5.9 (3.6-9)
*Giardia lamblia*	19,026 (15,668–22,473)	1.9 (0.9–3.4)	20,569 (16,878–23,919)	9.3 (4.2–16.3)	16,542 (13,391–19,879)	5.1 (2.9–8.2)	7,533 (5,979-9,333)	2.8 (2–4)	20,545 (16,641–24,479)	11.2 (7.3–16.4)	84,215 (62,578–100,083)	30.3 (17.3–48.2)
EHEC	3,091 (2,558-3,622)	1.1 (0.5–1.9)	4,392 (3,576-5,135)	5 (2.5–8.5)	3,809 (3,086–4,576)	2.2 (1.2–3.5)	1,818 (1,443-2,253)	1.1 (0.8–1.6)	3,341 (2,719-3,965)	7.4 (5.1–10.5)	16,451 (11,939–19,551)	16.8 (10.1–26)
EPEC	9,264 (7,643–10,913)	2.7 (1.3–4.6)	11,142 (9,113–12,985)	12.2 (5.8–21)	9,251 (7,492–11,116)	5.8 (3.3–9.3)	4,299 (3,412-5,326)	3.1 (2.1–4.4)	10,029 (8,136–11,933)	16.7 (11.2–23.9)	43,985 (32,384–52,273)	40.5 (23.7–63.2)
ETEC	12,051 (9,947–14,183)	1.5 (0.7–2.6)	14,931 (12,203–17,412)	7 (3.2–12.3)	12,500 (10,123–15,019)	3.8 (2.2-6)	5,837 (4,633-7,233)	2.1 (1.4-3)	13,054 (10,595–15,525)	8.7 (5.7–12.6)	58,373 (42,868–69,372)	23.1 (13.2–36.5)
EAEC	24,020 (19,769–28,398)	4.6 (2.2–8.1)	25,008 (20,546–29,058)	21.3 (10.1–36.6)	19,861 (16,075–23,869)	10.1 (5.8–16.2)	8,970 (7,120–11,114)	5.4 (3.7–7.6)	25,926 (20,989–30,965)	29 (19.6–41.6)	103,785 (77,379–123,344)	70.4 (41.4-110.1)
EIEC	7,921 (6,485-9,437)	14.7 (6.8–26.2)	5,474 (4,571-6,288)	70.5 (32.3-123.2)	3,598 (2,905-4,328)	37.3 (21.3–59.6)	1,407 (1,117-1,743)	20.6 (14.1–29.2)	8,486 (6,838–10,157)	87.9 (58-127.7)	26,886 (20,799–31,953)	231 (132.4-365.9)
STEC	13,924 (11,430–16,526)	0.1 (0.1–0.2)	12,081 (9,990–13,974)	0.7 (0.3–1.2)	8,946 (7,234–10,754)	0.5 (0.3–0.7)	3,846 (3,053–4,765)	0.3 (0.2–0.4)	14,982 (12,101–17,895)	0.6 (0.3–0.9)	53,779 (40,755–63,914)	2.2 (1.2–3.4)
*V. cholerae*	212 (173–254)	0.7 (0.3–1.4)	97 (83–109)	4.2 (1.7–7.7)	43 (35–52)	2.9 (1.7–4.7)	10 (8–12)	1.8 (1.2–2.5)	227 (182–272)	3.6 (2.1–5.6)	589 (473–699)	13.2 (7-21.9)
*Salmonella* spp.	1,385 (1,138-1,642)	0.1 (0.1–0.3)	1,280 (1,056–1,483)	0.7 (0.3–1.4)	972 (787-1,169)	0.6 (0.3–0.9)	425 (338–527)	0.3 (0.2–0.5)	1,492 (1,206-1,781)	0.6 (0.3–0.9)	5,554 (4,187-6,602)	2.3 (1.2-4)
*Shigella* spp.	3,156 (2,593-3,739)	2.3 (1.1-4)	2,953 (2,435-3,422)	10.6 (5-18.2)	2,257 (1,826-2,713)	5.1 (2.9–8.2)	993 (788-1,230)	2.8 (1.9–3.9)	3,400 (2,749-4,058)	14.1 (9.5–20.3)	12,759 (9,603–15,162)	34.9 (20.4–54.6)
Other	12,277 (10,150–14,415)	2 (0.9–3.6)	16,517 (13,469–19,290)	9.8 (4.5–17.2)	14,124 (11,441–16,969)	5.2 (3-8.4)	6,679 (5,302-8,276)	2.9 (2-4.1)	13,322 (10,828–15,824)	12.1 (8-17.6)	62,919 (45,888–74,774)	32 (18.4–50.9)
Unknown	27,371 (22,888–31,568)	2.9 (1.3–5.4)	58,031 (46,885–68,202)	14.9 (6.5–26.7)	54,026 (43,802–64,889)	9 (5.1–14.4)	26,798 (21,271–33,204)	5.1 (3.5–7.3)	30,214 (24,795–35,573)	16.3 (10.4–24.3)	196,440 (138,370–233,436)	48.2 (26.8–78.1))
Total	186,844 (154,120–220,131)	49.2 (22.9–87.7)	222,896 (182,360–259,733)	235.7 (108.5-410.7)	184,663 (149,537–221,890)	123 (70.3-196.6)	85,714 (68,036–106,203)	67.6 (46.3–96)	202,284 (164,085–240,707)	297.3 (196.8-431.2)	882,401 (650,102-1,048,664)	772.8 (444.8-1,222.2)

**Table 8:** Number of diarrheal illnesses and cases per 100,000 people for children ≥ 5 years of age and adults in African regions, with 95% uncertainty interval (UI), using the Global Burden of Disease incidence and mortality envelopes, 2019. Country-specific data were extracted from https://vizhub.healthdata.org/gbd-compare/

Pathogen	Northern Africa		Central Africa		Eastern Africa		Southern Africa		Western Africa		Whole African Region (1,1 bil. pop)	
	6 countries (212 mil. pop)		9 countries (144 mil. pop)		18 countries (366 mil. pop)		5 countries (60 mil. pop)		16 countries (327 mil. pop)			
	Illnesses (95% UI)	Deaths (95% UI)	Illnesses (95% UI)	Deaths (95% UI)	Illnesses (95% UI)	Deaths (95% UI)	Illnesses (95% UI)	Deaths (95% UI)	Illnesses (95% UI)	Deaths (95% UI)	Illnesses (95% UI)	Deaths (95% UI)
Rotavirus	3,799 (3,238-4,412)	0.2 (0.1–0.3)	1,948 (1,698-2,216)	3.1 (1.6–5.7))	1,246 (1,083–1,420)	3.6 (1.8–6.2)	1,083 (957-1,220)	3.1 (1.7–5.9)	4,168 (3,661-4,704)	2.8 (1.5–5.2)	12,244 (9,680–13,972)	12.8 (5-23.3)
Norovirus	2,239 (1,908-2,601)	0 (0-0.1)	2,152 (1,881-2,441)	0.5 (0.3–0.9)	1,873 (1,634-2,129)	0.5 (0.3–0.9)	2,014 (1,779-2,269)	0.5 (0.2–0.9)	2,645 (2,319-2,992)	0.6 (0.3–1.1)	10,923 (7,742–12,432)	2.1 (0.9–3.9)
Astrovirus	671 (572–780)	0 (0–0)	527 (461–599)	0 (0–0)	432 (376–491)	0 (0–0)	452 (400–510)	0 (0–0)	772 (677–872)	0.1 (0-0.2)	2,854 (2,086–3,252)	0.1 (0-0.3)
*Campylobacter* spp.	1,115 (950-1,296)	0 (0–0)	1,235 (1,080–1,401)	0.2 (0.1–0.4)	1,112 (980-1,276)	0.3 (0.1–0.5)	1,245 (1,100-1,403)	0.2 (0.1–0.5)	1,347 (1,180-1,524)	0.2 (0.1–0.3)	6,064 (4,190-6,900)	0.9 (0.3–1.7)
*Cryptosporidium* spp.	884 (754-1,027)	0 (0–0)	678 (592–770)	0.4 (0.2–0.7)	540 (470–614)	0.4 (0.2–0.7))	557 (492–627)	0.4 (0.2–0.7)	1,013 (889-1,144)	0.5 (0.2–0.8)	3,672 (2,705-4,182)	1.7 (0.6–2.9)
*Entamoeba histolytica*	2,800 (2,386-3,252)	0 (0-0.1)	1,819 (1,587-2,066)	0.5 (0.3–0.9)	1,355 (1,180-1,543)	0.5 (0.3–0.9)	1,332 (1,177-1,501)	0.4 (0.2–0.8)	3,145 (2,761-3,552)	0.6 (0.3-1)	10,451 (7,914–11,914)	2 (0.9–3.7)
*Giardia lamblia*	4,939 (4,209-5,738)	0.1 (0-0.1)	4,606 (4,025–5,226)	1.1 (0.5-2)	3,970 (3,464-4,514)	1.1 (0.6-2)	4,308 (3,807-4,855)	0.9 (0.5–1.8)	5,811 (5,096–6,571)	1.2 (0.6–2.2)	23,634 (16,794–26,904)	4.4 (1.7–8.1)
EHEC	573 (488–666)	0.1 (0-0.1)	665 (582–755)	0.7 (0.4–1.2)	612 (534–696)	0.4 (0.2–0.8)	684 (604–770)	0.2 (0.1–0.5)	699 (613–791)	1.4 (0.8–2.6)	3,233 (2,217-3,678)	2.8 (1.4–5.2)
EPEC	1,363 (1,162)	0.1 (0-0.2)	1,498 (1,310-1,699)	1.7 (0.9–3.1)	1,357 (1,184-1,542)	2.1 (1-3.7)	1,499 (1,325-1,689)	1.9 (1-3.6)	1,649 (1,445-1,866)	1.2 (0.6–2.2)	7,366 (5,101-8,380)	7 (2.5–12.8)
ETEC	3,193 (2,721-3,710)	0.1 (0-0.1)	3,394 (2,968-3,850)	1.2 (0.6–2.1)	3,044 (2,656-3,459)	1.1 (0.6-2)	3,387 (1,2994-3,817)	0.9 (0.5–1.8)	3,839 (3,365-4,344)	1.5 (0.8–2.7)	16,857 (11,710–19,180)	4.8 (2-8.7)
EAEC	2,337 (1,991-2,715)	0.2 (0.1–0.3)	2,247 (1,964-2,549)	2.8 (1.4–5.1)	1,952 (1,703-2,219)	3.1 (1.5–5.4)	2,116 (1,870-2,385)	2.7 (1.5–5.1)	2,759 (2,420-3,121)	2.8 (1.4-5)	11,411 (8,078–12,989)	11.6 (4.4–20.9)
EIEC	308 (263–358)	0.1 (0-0.2)	326 (285–369)	1.6 (0.8–2.8)	293 (255–333)	1.4 (0.7–2.4)	323 (285–364)	1.1 (0.6-2)	370 (342–418)	2.3 (1.2–4.3)	1,620 (1,127-1,842)	6.5 (2.7–11.7)
STEC	3,282 (2,796-3,816)	0 (0–0)	5,058 (4,426-5,733)	0.1 (0-0.1)	4,915 (4,293-5,583)	0.1 (0-0.2)	5,655 (4,998-6,373)	0.1 (0-0.2)	4,236 (3,707-4,801)	0.1 (0-0.1)	23,146 (15,222–26,306)	0.4 (0-0.6)
*V. cholerae*	474 (404–552)	0 (0–0)	1,229 (1,076–1,392)	0.1 (0.1–0.2)	1,280 (1,119-1,454)	0.1 (0-0.2)	1,516 (1,340-1,708)	0 (0-0.1)	707 (617–804)	0.3 (0.2–0.5)	5,206 (3,216-5,910)	0.5 (0.3-1)
*Salmonella* spp.	20,052 (17,085–23,301)	0 (0–0)	22,520 (19,692–25,540)	0.1 (0-0.1)	20,545 (17,934–23,349)	0.1 (0-0.2)	22,846 (20,190–25,746)	0.1 (0-0.2)	24,323 (21,316–27,527)	0.1 (0.1–0.2)	110,286 (76,027–125,463)	0.4 (0.1–0.8)
*Shigella* spp.	3,041 (2,590-3,537)	0.1 (0-0.2)	5,893 (5,159-6,677)	1.8 (0.9–3.3)	5,944 (5,194-6,750)	2 (1-3.5)	6,937 (6,130-7,818)	1.8 (1-3.3)	4,158 (3,635-4,719)	1.8 (0.9–3.2)	25,973 (16,578–29,501)	7.5 (2.8–13.5)
Other	1,880 (1,602-2,185)	0 (0-0.1)	2,134 (1,866-2,421)	0.8 (0.4–1.4)	1,954 (1,706-2,221)	0.8 (0.4–1.5)	2,184 (1,930-2,462)	0.7 (0.4–1.3)	2,283 (2,001–2,584)	0.9 (0.5–1.6)	10,435 (7,175–11,873)	3,2 (1.3–5.9)
Unknown	9,365 (7,978–10,891)	0.3 (0.1–0.5)	16,834 (14,737–19,075)	4.8 (2.4–8.7)	16,807 (14,684–19,088)	5 (2.5–8.7)	19,522 (17,252–21,999)	4.3 (2.3-8)	12,511 (10,941–14,193)	5.3 (2.8–9.7)	75,039 (48,340–85,246)	19.7 (7.8–35.6)
Total	62,315 (53,097–72,421)	1.3 (0.3–2.3)	74,763 (65,389–84,779)	21.5 (10.9–38.9)	69,241 (60,449–78,681)	22.6 (11.2–39.8)	77,660 (68,630–87,516)	19.3 (10.3–36.7)	76,435 (66,967–86,527)	23.7 (12.3–42.9)	260,414 (245,902–409,924)	88.4 (34.7-160.6)

## Discussion

Diarrheal disease continues to pose a significant burden on public health, particularly in low- and middle-income countries. In this study, we presented our findings on the incidence and mortality of diarrheal diseases in Africa. Our results showed that EAEC, *G. lamblia*, EIEC and rotavirus caused the highest number of diarrheal illnesses and deaths in the under-five age-group. For children above five and adults, we estimated *Salmonella* spp. and rotavirus to cause the largest number of diarrheal illnesses and deaths.

Our estimates also showed that rotavirus continues to continue to cause a high diarrheal mortality in children below five, even after the introduction of vaccination. According to the WHO, only about 52% of the African population has been vaccinated against rotavirus since the introduction of the rotavirus vaccination program [28]. This could explain why the diarrheal mortality of rotavirus is still high in Africa, which other studies have also found [29, 30]. We found Western Africa to have the highest diarrheal mortality, and Eastern Africa to have the highest diarrheal incidence, for children < five and the population above five. We also found Southern Africa to have the lowest number of diarrheal incidence and mortality across all age-groups.

Our estimates of the etiology proportions are aligned with previous studies, showing that rotavirus and *E. coli*, particularly EAEC and EHEC, are two of the most common causes of diarrhea in low-income countries [1, 8]. Incidence of diarrhea in children below five caused by other pathogens like *G. lamblia* and norovirus was also estimated to be high, which supports the findings of Pires et al. (2015) and the MAL-ED by Platts-Mills et al. (2015) [7, 8]. We found *Salmonella* spp. to have the largest etiology proportion in all ages; while the data were scarce for this age group, Marks et al. found the incidence of *Salmonella* to be very high across sub-Saharan Africa [31]. Water bacteriology reports from various African countries confirmed that *E. coli* and *Salmonella* spp. were abundant in African water sources, however no reports presented prevalence at pathotype level [32–34].

Some of the findings of our study did not align with earlier studies. EIEC was found to be the largest contributor of diarrhea in children below five, which suggests that most of the diarrhoeagenic *E. coli* infections were attributed to this pathotype. While other pathotypes of *E. coli* have previously been associated with endemic diarrhea in Africa and other LMIC, EIEC has been reported sporadically with low frequency in Africa, and the MAL-ED study reported that less than 1% of the diarrheal cases for children < 2 could be attributed to EIEC [35, 36]. This discrepancy may be caused by the limited number of studies contributing to the pooled estimates for EIEC and emphasizes the need for caution when interpreting these results. Findings like these highlight the need for more comprehensive surveillance. Increasing focus on different types of diarrheagenic *E. coli* and other pathogens will enable more accurate estimates.

We also found that the parasites *E. histolytica* and *Cryptosporidium spp.* caused a smaller attributable proportion of diarrhea relative to *E. coli* and rotavirus, in particular in children below five, while the MAL-ED study reported *Cryptosporidium* to have a high burden of childhood diarrhea. As the data were scarce, it is possible that our estimates were biased towards studies only testing for viruses and bacteria. However, we included viral and bacterial pathogens that previous studies did not include. Specifically, Pires et al. did not include rotavirus and EAEC/EHEC/EIEC/STEC, and MAL-ED did not include EIEC/STEC/EAEC/EHEC. The higher number of pathogens included in our study might explain the relatively lower etiology proportions for parasites.

To estimate the contribution of different etiologies to deaths caused by diarrhea, we assumed that the patients admitted to the hospital have more severe disease and a higher risk of death, and that the etiology distributions as collected from these studies could be used as a proxy for the etiology distribution of diarrheal deaths. We recognize that this could lead to an overestimation of the true number of deaths in some cases, such as EIEC and rotavirus.

In some study-settings, there were no studies reporting prevalence of certain pathogens, which meant that we had to extrapolate the pooled proportions for some of the pathogens that did not have an estimate. This was especially evident for the above five category. For instance, we assumed that the proportion of rotavirus infections was similar in children below five and the population above five, and applied the pooled proportion estimates from children below five in the out-patient/community group to estimate rotavirus incidence in the population above five [7]. This is a limitation. Although previous studies estimated similar prevalence of rotavirus in children under five and the general population (i.e. all ages) in Africa, other studies noted that the prevalence is lower in adults [37, 38]. Furthermore, some pathotypes of *E. coli* were well-covered, especially in the below five-category, despite the low number of data points, but very few studies reported data for EIEC and STEC. This was reflected quantitatively in the uncertainty estimates, which were broad for the pathogens with a low number of studies to support the estimates. Despite the low number of data points, we have achieved comprehensive coverage by collecting data from all five regions across 17 countries. This ensures that our analysis reflects a wide spectrum of conditions in Africa. The majority of data pertained to the under-five age group, with data gaps for those over five. Our findings are robust for rotavirus, norovirus, and common DEC-pathotypes, supported by extensive research. However, there was a shortage of studies on astrovirus, *Vibrio cholerae*, *Shigella*, and *Salmonella*, indicating a need for further research and surveillance to better understand their epidemiology in the region.

The low number of studies available to estimate etiology proportions also meant that many countries were not represented in the estimates. Therefore, to estimate regional incidence and mortality for different pathogens, we assumed that the distribution of pathogens represented the proportion of pathogens in all regions and pooled studies across all countries and regions to estimate etiology proportions. This approach has limitations, as the epidemiology of pathogens can vary across countries. To address this, we only included studies that were broadly representative of general population, and employed a pooled proportion methodology within our meta-analytic framework.

We also recognize the large proportion of diarrheal illnesses attributed to the “unknown” etiology, which accounted for close to 1/3 of the illnesses in the older age-group (children above five and adults). Diarrhea cases with unknown etiology could be due to the use of insensitive methods to identify them, to the use of antibiotics prior to obtaining the stool sample, to other yet undiscovered infections, or to non-infectious causes of diarrhea [20]. It is also possible that there are pathogens that are more frequent in > 5 that we did not include in the review, because there were fewer data for this age group [20, 39].

Prior work by Pires et al. (2015) [7] presented the first global and regional estimates of incidence and mortality caused by nine foodborne pathogens. They estimated a total of 699,444,445 (95% UI: 315,105,085 − 1,545,303,636) million illnesses and 662,862 (95% UI: 561,804 − 778,865) deaths caused by diarrhea in the African region in 2010. Taking the region’s population increase into account, which has increased from 850 million to more than 1,1 billion people in the last decade [40], our results show a slight increase in the total number of illnesses. We estimated 90,051 (95% UI: 39,969 − 127,731) cases of diarrheal per 100,000 people, whereas Pires et al. estimated 81,436 cases per 100,000 people (95% UI: 36,687 − 179,918). With the marked population growth in the African region in last years, increased strain on healthcare systems and potential shifts in environmental conditions could lead to more cases of diarrhea in the general population [41].

When comparing our overall diarrhea incidence and mortality results with the 2019 GBD estimates of 1,170,750,773 diarrheal cases (95% UI: 952,463,919-1,351,848,685) and 569,072 (313,861–933,633) diarrheal deaths in 2019 in the African region, our results show fewer diarrheal cases and deaths. The differences in diarrhea incidence may be due to the COVID-19 pandemic, which led to significant changes in human interaction and societal behavior [42, 43]. For example, a behavioral study from South Africa showed enhanced behavioral responsiveness to COVID-19 [43], and one cross-sectional study targeting five developing countries (Cameroon, Ethiopia, Ghana, Kenya, and Nigeria) found that awareness of the pandemic led to a reduction in street foods consumption, a rise in the preference for cooked foods, and a greater awareness of hygiene during food preparation [42]. The diarrhea incidence estimates used in our study were generated through a population-based survey conducted in four African countries between 2020 and 2021, i.e., after surveyed populations had lived through a pandemic and the many lifestyle and hygienic behavior changes observed globally in that period. As governments instituted lockdowns, travel restrictions and increased attention to hygiene to limit the number of infected people, this could have led to many people becoming more aware about the importance of hand washing and sanitation, which in turn could have decreased the exposure to pathogens related to food-borne illnesses [27].

Using the diarrhea incidence estimates by Desta et al., we found Eastern Africa to have the incidence of diarrhea across all age groups. Other studies have also found Eastern Africa to have the highest incidence of diarrheal diseases among children when compared to the rest of the world [44, 45]. The large proportion of the population in Eastern Africa residing in rural areas with limited access to water and sanitation could explain the high incidence of diarrhea.

In this study, we have provided an analysis of the etiologies of diarrheal diseases within an “African” setting. We acknowledge that the term “African” encompasses a vast array of diverse countries and populations. The data available were fragmented, and there was a scarcity of studies focusing on specific countries or regions within the continent. This scarcity of data resulted in estimates that might not accurately represent the nuances of each unique setting. Despite these constraints, we believe that that our findings represent the best possible estimates given the current scope of data. This approach is supported by our comprehensive uncertainty assessment.

Despite limitations, our results provide new information on the incidence and mortality of 16 pathogens that cause diarrhea. Even though widespread efforts to combat rotavirus has been introduced to Africa, namely by the implementation of vaccine programs, rotavirus is still one of the largest attributors of diarrhea [46]. Similarly, with diarrhea-inducing *E. coli*, the large burden of these pathogens suggests that targeted interventions may be necessary to reduce the mortality and burden of illness of diarrhea substantially. To estimate the true burden of disease, our results will need to be translated into disability-adjusted life years (DALYs). Furthermore, several of these pathogens can infect humans through various transmission routes sources (i.e., contaminated foods, person-to-person contact, animal-to-human contact or environmental). Thus, estimates of the contribution of different transmission routes sources of infection are important for policymakers and other stakeholder to define and regulate control strategies to reduce the number of diarrheal infections. We also found substantial variation in the ranking of different pathogens causing diarrhea cases and deaths, even after stratifying for age and geographical area, which suggests that both local and regional strategies will have an important role in reducing the burden of diarrheal disease.

### Electronic supplementary material

Below is the link to the electronic supplementary material.

Supplementary Material 1

## Data Availability

All results and data generated is provided within the manuscript or supplementary information files.

## Declarations

Shannon Majowicz is an expert on the Joint FAO/WHO Expert Meetings on Microbiological Risk Assessment (JEMRA) Roster of Experts.

## References

[1] World Health Organization. Diarrhoeal disease [Internet]. 2017 [cited 2022 Dec 2]. https://www.who.int/news-room/fact-sheets/detail/diarrhoeal-disease.

[2] Grace D (2015). Food Safety in Low and Middle Income Countries. Int J Environ Res Public Health.

[3] World Health Organization. Food safety [Internet]. 2022 [cited 2023 Nov 23]. https://www.who.int/news-room/fact-sheets/detail/food-safety.

[4] Murray CJ, Lopez AD (1997). Global mortality, disability, and the contribution of risk factors: global burden of Disease Study. Lancet.

[5] Null C, Stewart CP, Pickering AJ, Dentz HN, Arnold BF, Arnold CD (2018). Effects of water quality, sanitation, handwashing, and nutritional interventions on diarrhoea and child growth in rural Kenya: a cluster-randomised controlled trial. Lancet Glob Health.

[6] Donders ART, van der Heijden GJMG, Stijnen T, Moons KGM (2006). Review: a gentle introduction to imputation of missing values. J Clin Epidemiol.

[7] Pires SM, Fischer-Walker CL, Lanata CF, Devleesschauwer B, Hall AJ, Kirk MD (2015). Aetiology-specific estimates of the Global and Regional Incidence and Mortality of Diarrhoeal diseases commonly transmitted through Food. PLoS ONE.

[8] Platts-Mills JA, Babji S, Bodhidatta L, Gratz J, Haque R, Havt A (2015). Pathogen-specific burdens of community diarrhoea in developing countries: a multisite birth cohort study (MAL-ED). Lancet Glob Health.

[9] Levine MM, Kotloff KL, Nataro JP, Muhsen K (2012). The global enteric Multicenter Study (GEMS): impetus, Rationale, and Genesis. Clin Infect Dis.

[10] GBD 2019 Child and Adolescent Communicable Disease Collaborators (2023). The unfinished agenda of communicable diseases among children and adolescents before the COVID-19 pandemic, 1990–2019: a systematic analysis of the global burden of Disease Study 2019. Lancet.

[11] Institute for Health Metrics and Evaluation (IHME). Global Burden of Disease Collaborative Network. Global Burden of Disease Study 2019 (GBD 2019) Results [Internet]. 2020 [cited 2023 Sep 1]. https://vizhub.healthdata.org/gbd-results.

[12] Hasso-Agopsowicz M, Lopman BA, Lanata CF, Rogawski McQuade ET, Kang G, Prudden HJ, World Health Organization Expert Working Group, et al. Recommendations for assessing morbidity associated with enteric pathogens. Vaccine. Elsevier Ltd; 2021. pp. 7521–5. 10.1016/j.vaccine.2021.11.033 34838322

[13] Prudden HJ, Hasso-Agopsowicz M, Black RE, Troeger C, Reiner RC, Breiman RF et al. November. Meeting Report: WHO Workshop on modelling global mortality and aetiology estimates of enteric pathogens in children under five. Cape Town, 28–29th 2018. In: Vaccine. Elsevier Ltd; 2020. pp. 4792–800. 10.1016/j.vaccine.2020.01.054 PMC7306158 32253097

[14] Butkeviciute E, Prudden HJ, Jit M, Smith PG, Kang G, Riddle MS (2021). Global diarrhoea-associated mortality estimates and models in children: recommendations for dataset and study selection.

[15] World Health Organization. Global Health Estimates 2019: Deaths by Cause, Age, Sex, by Country and by Region, 2000–2019 [Internet]. 2020 [cited 2022 Dec 21]. https://www.who.int/data/gho/data/themes/mortality-and-global-health-estimates.

[16] Liu J, Platts-Mills JA, Juma J, Kabir F, Nkeze J, Okoi C (2016). Use of quantitative molecular diagnostic methods to identify causes of diarrhoea in children: a reanalysis of the GEMS case-control study. Lancet.

[17] Foodborne Disease Burden Epidemiology Reference Group (FERG). Monitoring Nutritional Status & Food Safety Events (MNF), Nutrition and Food Safety (NFS). WHO estimates of the global burden of foodborne diseases: foodborne diseases burden epidemiology reference group 2007–2015. 2015.

[18] Stevens GA, Alkema L, Black RE, Boerma JT, Collins GS, Ezzati M (2016). Guidelines for Accurate and Transparent Health estimates reporting: the GATHER statement. Lancet.

[19] Sharrow D, Hug L, You D, Alkema L, Black R, Cousens S (2022). Global, regional, and national trends in under-5 mortality between 1990 and 2019 with scenario-based projections until 2030: a systematic analysis by the UN Inter-agency Group for Child Mortality Estimation. Lancet Glob Health.

[20] Lanata CF, Fischer-Walker CL, Olascoaga AC, Torres CX, Aryee MJ, Black RE. Global Causes of Diarrheal Disease Mortality in Children <5 Years of Age: A Systematic Review. PLoS One. 2013 Sep 4;8(9):e72788 10.1371/journal.pone.0072788 PMC3762858 24023773

[21] Kirk MD, Pires SM, Black RE, Caipo M, Crump JA, Devleesschauwer B et al. World Health Organization Estimates of the Global and Regional Disease Burden of 22 foodborne bacterial, Protozoal, and viral diseases, 2010: A Data Synthesis. PLoS Med. 2015;12(12). 10.1371/journal.pmed.1001921 PMC4668831 26633831

[22] Knapp G, Hartung J (2003). Improved tests for a random effects meta-regression with a single covariate. Stat Med.

[23] Lipsey MW, Wilson DB. Practical Meta-analysis. Volume 49. SAGE Publications Inc.; 2000.

[24] Higgins JP, Thomas J, Chandler J, Cumpston M, Li T, Page MJ et al. Cochrane. 2022 [cited 2022 Sep 16]. Cochrane Handbook for Systematic Reviews of interventions. http://www.training.cochrane.org/handbook.

[25] Viechtbauer W, Cheung MWL (2010). Outlier and influence diagnostics for meta-analysis. Res Synth Methods.

[26] Egger M, Smith GD, Schneider M, Minder C (1997). Bias in meta-analysis detected by a simple, graphical test. BMJ.

[27] Desta B. The Epidemiology of Acute Gastrointestinal Illness in Ethiopia, Mozambique, Nigeria, and Tanzania [PhD Thesis]. [Waterloo]: University of Waterloo; 2022. 10.1017/S095026882500038X PMC12086602 40256804

[28] World Health Organization. Introduction of rotavirus vaccine [Internet]. [cited 2023 Aug 2]. https://immunizationdata.who.int/pages/vaccine-intro-by-antigen/rotavirus.html?ISO_3_CODE=&YEAR=

[29] Henschke N, Bergman H, Hungerford D, Cunliffe NA, Grais RF, Kang G (2022). The efficacy and safety of rotavirus vaccines in countries in Africa and Asia with high child mortality. Vaccine.

[30] Steele AD, Armah GE, Mwenda JM, Kirkwood CD (2023). The full impact of Rotavirus vaccines in Africa has yet to be realized. Clin Infect Dis.

[31] Marks F, von Kalckreuth V, Aaby P, Adu-Sarkodie Y, El Tayeb MA, Ali M (2017). Incidence of invasive salmonella disease in sub-saharan Africa: a multicentre population-based surveillance study. Lancet Glob Health.

[32] Idowu A, Oluremi B, Odubawo K. Bacteriological analysis of well water samples in Sagamu. Afr J Clin Experimental Microbiol. 2011;12(2).

[33] Gebrewahd A, Adhanom G, Gebremichail G, Kahsay T, Berhe B, Asfaw Z (2020). Bacteriological quality and associated risk factors of drinking water in Eastern Zone, Tigrai, Ethiopia, 2019. Trop Dis Travel Med Vaccines.

[34] Sila ON (2019). Physico-chemical and bacteriological quality of water sources in rural settings, a case study of Kenya, Africa. Sci Afr.

[35] Rappelli P, Folgosa E, Solinas ML, DaCosta JL, Pisanu C, Sidat M (2005). Pathogenic enteric Escherichia coli in children with and without diarrhea in Maputo, Mozambique. FEMS Immunol Med Microbiol.

[36] Ochieng JB, Powell H, Sugerman CE, Omore R, Ogwel B, Juma J (2023). Epidemiology of Enteroaggregative, Enteropathogenic, and Shiga Toxin–Producing Escherichia coli among children aged < 5 years in 3 countries in Africa, 2015–2018: vaccine impact on Diarrhea in Africa (VIDA) Study. Clin Infect Dis.

[37] Raini S, Nyangao J, Kombich J, Sang C, Gikonyo J, Ongus J (2015). Human rotavirus group a serotypes causing gastroenteritis in children less than 5 years and HIV-infected adults in Viwandani Slum, Nairobi. Ethiop J Health Sci.

[38] Njifon HLM, Kenmoe S, Ahmed SM, Roussel Takuissu G, Ebogo-Belobo JT, Njile DK (2024). Epidemiology of Rotavirus in humans, animals, and the Environment in Africa: a systematic review and Meta-analysis. J Infect Dis.

[39] Reisinger EC, Fritzsche C, Krause R, Krejs GJ (2005). Diarrhea caused by primarily non-gastrointestinal infections. Nat Clin Pract Gastroenterol Hepatol.

[40] WHO Regional Office for Africa. Atlas of African Health Statistics. 2022: Health situation analysis of the WHO African Region [Internet]. 2022 [cited 2023 Sep 16]. https://www.afro.who.int/publications/atlas-african-health-statistics-2022-health-situation-analysis-who-african-region-0.

[41] Wang P, Asare E, Pitzer VE, Dubrow R, Chen K (2022). Associations between long-term drought and diarrhea among children under five in low- and middle-income countries. Nat Commun.

[42] Tchuenchieu Kamgain AD, Kesa H, Onyenweaku EO (2022). Food safety behavioural changes among the population in Sub-saharan Africa during the COVID-19 first wave. Heliyon.

[43] Kollamparambil U, Oyenubi A (2021). Behavioural response to the Covid-19 pandemic in South Africa. PLoS ONE.

[44] O Connell BJ, Quinn MA, Scheuerman P. Risk factors of diarrheal disease among children in the east African countries of Burundi, Rwanda and Tanzania. Global J Med Public Health. 2017;6(1).

[45] Reiner RC, Graetz N, Casey DC, Troeger C, Garcia GM, Mosser JF (2018). Variation in Childhood Diarrheal Morbidity and Mortality in Africa, 2000–2015. N Engl J Med.

[46] Crawford SE, Ramani S, Tate JE, Parashar UD, Svensson L, Hagbom M (2017). Rotavirus infection. Nat Rev Dis Primers.

