# Supplementary material for: Etiology-specific incidence and mortality of diarrheal diseases in the African region: a systematic review and meta-analysis
Source: BMC Public Health. 2024 Jul 12;24:1864. doi: 10.1186/s12889-024-19334-8 (PMC11241906; doi:10.1186/s12889-024-19334-8)
Supplement: Supplementary file 1 — Supplementary Material 1 [file 12889_2024_19334_MOESM1_ESM.docx]

**Supplementary Material**

Etiology-specific incidence and mortality of diarrheal diseases in the African region: a systematic review and meta-analysis

Thystrup et al.

Contents

[**Supplementary Search String 1**: Search String for PubMed 2](#_Toc158116079)

[**Supplementary Search String 2**: Search String for SCOPUS 8](#_Toc158116080)

[**Supplementary Table S1:** Search String for Web of Science 9](#_Toc158116081)

[**Supplementary Table S2:** Characteristics of the studies included in the review (n = 38). 10](#_Toc158116082)

[**Supplementary Table S3:** Detailed description of the studies reporting incidence of diarrhea meeting inclusion criteria, stratified by age group and/or study setting 16](#_Toc158116083)

[**Supplementary Table S4:** Pooled proportion estimates of the studies conducted in children <5 years of age, in an in-patient setting. 36](#_Toc158116084)

[**Supplementary Table S5:** Pooled proportion estimates of the studies conducted in children ≥5 years of age and adults, in an in-patient setting. 39](#_Toc158116085)

[**Supplementary Table S6:** Pooled proportion estimates of the studies conducted in children <5 years of age, in an out-patient and community settings. 41](#_Toc158116086)

[**Supplementary Table S7:** Pooled proportion estimates of the studies conducted in children ≥5 years of age and adults, in an out-patient and community settings. 45](#_Toc158116087)

[**Supplementary Table S8:** Pooled proportion estimates of norovirus, EPEC and ETEC before and after adjusting for potential carriage of pathogens unrelated to diarrhea. 47](#_Toc158116088)

[**Supplementary Table S9:** P-value obtained from the Egger’s test testing for asymmetry, with number of studies (n). InS = Insufficient number of trials (n<10) 47](#_Toc158116089)

[**Supplementary Table S10:** Incidence and population estimates from Desta et al.^14^ and WHO Global Health Observatory 48](#_Toc158116090)

[**Supplementary Table S11:** Incidence and mortality envelope obtained from Global Burden of Disease incidence and mortality envelopes, 2019. Country-specific data were extracted from https://vizhub.healthdata.org/gbd-compare/. 49](#_Toc158116091)

# **Supplementary Search String 1**: Search String for PubMed

“2021/04/30”[PDat]) AND (Humans[Mesh]) AND (Clinical Trial[ptyp] ORMeta-Analysis[ptyp] OR Randomized Controlled Trial[ptyp] OR Review[ptyp]OR Clinical Trial, Phase I[ptyp] OR Clinical Trial, Phase II[ptyp] OR Clini-cal Trial, Phase III[ptyp] OR Clinical Trial, Phase IV[ptyp] OR ComparativeStudy[ptyp] OR Controlled Clinical Trial[ptyp] OR Corrected and Repub-lished Article[ptyp] OR English Abstract[ptyp] OR Evaluation Studies[ptyp]OR Journal Article[ptyp] OR Multicenter Study[ptyp] OR Research Support,N I H, Extramural[ptyp] OR Research Support, N I H, Intramural[ptyp] ORResearch Support, Non U S Gov’t[ptyp] OR Research Support, U S Gov’t,Non P H S[ptyp] OR Research Support, U S Gov’t, P H S[ptyp] OR TwinStudy[ptyp] OR Validation Studies[ptyp]) AND (child[MeSH:noexp] OR ado-lescent[MeSH] OR adult[MeSH:noexp] OR middle age[MeSH] OR (middleage[MeSH] OR aged[MeSH]) OR aged[MeSH] OR aged, 80 and over[MeSH]))))NOT (((diarrhea AND morbidity AND ((“2014/01/01”[PDat] : “2021/04/30”[PDat])AND (Humans[Mesh]) AND (Clinical Trial[ptyp] OR Meta-Analysis[ptyp]OR Randomized Controlled Trial[ptyp] OR Review[ptyp] OR Clinical Trial,Phase I[ptyp] OR Clinical Trial, Phase II[ptyp] OR Clinical Trial, Phase III[ptyp]OR Clinical Trial, Phase IV[ptyp] OR Comparative Study[ptyp] OR Con-trolled Clinical Trial[ptyp] OR Corrected and Republished Article[ptyp] OREnglish Abstract[ptyp] OR Evaluation Studies[ptyp] OR Journal Article[ptyp]OR Multicenter Study[ptyp] OR Research Support, N I H, Extramural[ptyp]OR Research Support, N I H, Intramural[ptyp] OR Research Support, NonU S Gov’t[ptyp] OR Research Support, U S Gov’t, Non P H S[ptyp] ORResearch Support, U S Gov’t, P H S[ptyp] OR Twin Study[ptyp] OR Val-idation Studies[ptyp]) AND (child[MeSH:noexp] OR adolescent[MeSH] ORadult[MeSH:noexp] OR middle age[MeSH] OR (middle age[MeSH] OR aged[MeSH])OR aged[MeSH] OR aged, 80 and over[MeSH]))) OR (diarrhea AND mortal-ity AND ((“2014/01/01”[PDat] : “2021/04/30”[PDat]) AND (Humans[Mesh])AND (Clinical Trial[ptyp] OR Meta-Analysis[ptyp] OR Randomized Con-trolled Trial[ptyp] OR Review[ptyp] OR Clinical Trial, Phase I[ptyp] OR Clin-ical Trial, Phase II[ptyp] OR Clinical Trial, Phase III[ptyp] OR Clinical Trial,Phase IV[ptyp] OR Comparative Study[ptyp] OR Controlled Clinical Trial[ptyp]OR Corrected and Republished Article[ptyp] OR English Abstract[ptyp] OREvaluation Studies[ptyp] OR Journal Article[ptyp] OR Multicenter Study[ptyp]OR Research Support, N I H, Extramural[ptyp] OR Research Support, NI H, Intramural[ptyp] OR Research Support, Non U S Gov’t[ptyp] OR Re-search Support, U S Gov’t, Non P H S[ptyp] OR Research Support, U SGov’t, P H S[ptyp] OR Twin Study[ptyp] OR Validation Studies[ptyp]) AND (child[MeSH:noexp] OR adolescent[MeSH] OR adult[MeSH:noexp] OR mid-dle age[MeSH] OR (middle age[MeSH] OR aged[MeSH]) OR aged[MeSH]OR aged, 80 and over[MeSH]))) OR (diarrhea AND etiology AND ((“2014/01/01”[PDat]: “2021/04/30”[PDat]) AND (Humans[Mesh]) AND (Clinical Trial[ptyp] ORMeta-Analysis[ptyp] OR Randomized Controlled Trial[ptyp] OR Review[ptyp]OR Clinical Trial, Phase I[ptyp] OR Clinical Trial, Phase II[ptyp] OR Clini-cal Trial, Phase III[ptyp] OR Clinical Trial, Phase IV[ptyp] OR ComparativeStudy[ptyp] OR Controlled Clinical Trial[ptyp] OR Corrected and Repub-lished Article[ptyp] OR English Abstract[ptyp] OR Evaluation Studies[ptyp]OR Journal Article[ptyp] OR Multicenter Study[ptyp] OR Research Support,N I H, Extramural[ptyp] OR Research Support, N I H, Intramural[ptyp] ORResearch Support, Non U S Gov’t[ptyp] OR Research Support, U S Gov’t,Non P H S[ptyp] OR Research Support, U S Gov’t, P H S[ptyp] OR TwinStudy[ptyp] OR Validation Studies[ptyp]) AND (child[MeSH:noexp] OR ado-lescent[MeSH] OR adult[MeSH:noexp] OR middle age[MeSH] OR (middleage[MeSH] OR aged[MeSH]) OR aged[MeSH] OR aged, 80 and over[MeSH]))))AND cancer)) NOT ((((diarrhea AND morbidity AND ((“2014/01/01”[PDat]: “2021/04/30”[PDat]) AND (Humans[Mesh]) AND (Clinical Trial[ptyp] ORMeta-Analysis[ptyp] OR Randomized Controlled Trial[ptyp] OR Review[ptyp]OR Clinical Trial, Phase I[ptyp] OR Clinical Trial, Phase II[ptyp] OR Clini-cal Trial, Phase III[ptyp] OR Clinical Trial, Phase IV[ptyp] OR ComparativeStudy[ptyp] OR Controlled Clinical Trial[ptyp] OR Corrected and Repub-lished Article[ptyp] OR English Abstract[ptyp] OR Evaluation Studies[ptyp]OR Journal Article[ptyp] OR Multicenter Study[ptyp] OR Research Support,N I H, Extramural[ptyp] OR Research Support, N I H, Intramural[ptyp] ORResearch Support, Non U S Gov’t[ptyp] OR Research Support, U S Gov’t,Non P H S[ptyp] OR Research Support, U S Gov’t, P H S[ptyp] OR TwinStudy[ptyp] OR Validation Studies[ptyp]) AND (child[MeSH:noexp] OR ado-lescent[MeSH] OR adult[MeSH:noexp] OR middle age[MeSH] OR (middleage[MeSH] OR aged[MeSH]) OR aged[MeSH] OR aged, 80 and over[MeSH])))OR (diarrhea AND mortality AND ((“2014/01/01”[PDat] : “2021/04/30”[PDat])AND (Humans[Mesh]) AND (Clinical Trial[ptyp] OR Meta-Analysis[ptyp]OR Randomized Controlled Trial[ptyp] OR Review[ptyp] OR Clinical Trial,Phase I[ptyp] OR Clinical Trial, Phase II[ptyp] OR Clinical Trial, Phase III[ptyp]OR Clinical Trial, Phase IV[ptyp] OR Comparative Study[ptyp] OR Con-trolled Clinical Trial[ptyp] OR Corrected and Republished Article[ptyp] OREnglish Abstract[ptyp] OR Evaluation Studies[ptyp] OR Journal Article[ptyp]OR Multicenter Study[ptyp] OR Research Support, N I H, Extramural[ptyp]

Appendix A. Appendix A: Search strings65OR Research Support, N I H, Intramural[ptyp] OR Research Support, NonU S Gov’t[ptyp] OR Research Support, U S Gov’t, Non P H S[ptyp] ORResearch Support, U S Gov’t, P H S[ptyp] OR Twin Study[ptyp] OR Val-idation Studies[ptyp]) AND (child[MeSH:noexp] OR adolescent[MeSH] ORadult[MeSH:noexp] OR middle age[MeSH] OR (middle age[MeSH] OR aged[MeSH])OR aged[MeSH] OR aged, 80 and over[MeSH]))) OR (diarrhea AND etiologyAND ((“2014/01/01”[PDat] : “2021/04/30”[PDat]) AND (Humans[Mesh])AND (Clinical Trial[ptyp] OR Meta-Analysis[ptyp] OR Randomized Con-trolled Trial[ptyp] OR Review[ptyp] OR Clinical Trial, Phase I[ptyp] OR Clin-ical Trial, Phase II[ptyp] OR Clinical Trial, Phase III[ptyp] OR Clinical Trial,Phase IV[ptyp] OR Comparative Study[ptyp] OR Controlled Clinical Trial[ptyp]OR Corrected and Republished Article[ptyp] OR English Abstract[ptyp] OREvaluation Studies[ptyp] OR Journal Article[ptyp] OR Multicenter Study[ptyp]OR Research Support, N I H, Extramural[ptyp] OR Research Support, NI H, Intramural[ptyp] OR Research Support, Non U S Gov’t[ptyp] OR Re-search Support, U S Gov’t, Non P H S[ptyp] OR Research Support, U SGov’t, P H S[ptyp] OR Twin Study[ptyp] OR Validation Studies[ptyp]) AND(child[MeSH:noexp] OR adolescent[MeSH] OR adult[MeSH:noexp] OR mid-dle age[MeSH] OR (middle age[MeSH] OR aged[MeSH]) OR aged[MeSH]OR aged, 80 and over[MeSH])))) NOT (((diarrhea AND morbidity AND ((“2014/01/01”[PDat]: “2021/04/30”[PDat]) AND (Humans[Mesh]) AND (Clinical Trial[ptyp] ORMeta-Analysis[ptyp] OR Randomized Controlled Trial[ptyp] OR Review[ptyp]OR Clinical Trial, Phase I[ptyp] OR Clinical Trial, Phase II[ptyp] OR Clini-cal Trial, Phase III[ptyp] OR Clinical Trial, Phase IV[ptyp] OR ComparativeStudy[ptyp] OR Controlled Clinical Trial[ptyp] OR Corrected and Repub-lished Article[ptyp] OR English Abstract[ptyp] OR Evaluation Studies[ptyp]OR Journal Article[ptyp] OR Multicenter Study[ptyp] OR Research Support,N I H, Extramural[ptyp] OR Research Support, N I H, Intramural[ptyp] ORResearch Support, Non U S Gov’t[ptyp] OR Research Support, U S Gov’t,Non P H S[ptyp] OR Research Support, U S Gov’t, P H S[ptyp] OR TwinStudy[ptyp] OR Validation Studies[ptyp]) AND (child[MeSH:noexp] OR ado-lescent[MeSH] OR adult[MeSH:noexp] OR middle age[MeSH] OR (middleage[MeSH] OR aged[MeSH]) OR aged[MeSH] OR aged, 80 and over[MeSH])))OR (diarrhea AND mortality AND ((“2014/01/01”[PDat] : “2021/04/30”[PDat])AND (Humans[Mesh]) AND (Clinical Trial[ptyp] OR Meta-Analysis[ptyp]OR Randomized Controlled Trial[ptyp] OR Review[ptyp] OR Clinical Trial,Phase I[ptyp] OR Clinical Trial, Phase II[ptyp] OR Clinical Trial, Phase III[ptyp] OR Clinical Trial, Phase IV[ptyp] OR Comparative Study[ptyp] OR Con-trolled Clinical Trial[ptyp] OR Corrected and Republished Article[ptyp] OREnglish Abstract[ptyp] OR Evaluation Studies[ptyp] OR Journal Article[ptyp]OR Multicenter Study[ptyp] OR Research Support, N I H, Extramural[ptyp]OR Research Support, N I H, Intramural[ptyp] OR Research Support, NonU S Gov’t[ptyp] OR Research Support, U S Gov’t, Non P H S[ptyp] ORResearch Support, U S Gov’t, P H S[ptyp] OR Twin Study[ptyp] OR Val-idation Studies[ptyp]) AND (child[MeSH:noexp] OR adolescent[MeSH] ORadult[MeSH:noexp] OR middle age[MeSH] OR (middle age[MeSH] OR aged[MeSH])OR aged[MeSH] OR aged, 80 and over[MeSH]))) OR (diarrhea AND etiologyAND ((“2014/01/01”[PDat] : “2021/04/30”[PDat]) AND (Humans[Mesh])AND (Clinical Trial[ptyp] OR Meta-Analysis[ptyp] OR Randomized Con-trolled Trial[ptyp] OR Review[ptyp] OR Clinical Trial, Phase I[ptyp] OR Clin-ical Trial, Phase II[ptyp] OR Clinical Trial, Phase III[ptyp] OR Clinical Trial,Phase IV[ptyp] OR Comparative Study[ptyp] OR Controlled Clinical Trial[ptyp]OR Corrected and Republished Article[ptyp] OR English Abstract[ptyp] OREvaluation Studies[ptyp] OR Journal Article[ptyp] OR Multicenter Study[ptyp]OR Research Support, N I H, Extramural[ptyp] OR Research Support, NI H, Intramural[ptyp] OR Research Support, Non U S Gov’t[ptyp] OR Re-search Support, U S Gov’t, Non P H S[ptyp] OR Research Support, U SGov’t, P H S[ptyp] OR Twin Study[ptyp] OR Validation Studies[ptyp]) AND(child[MeSH:noexp] OR adolescent[MeSH] OR adult[MeSH:noexp] OR mid-dle age[MeSH] OR (middle age[MeSH] OR aged[MeSH]) OR aged[MeSH]OR aged, 80 and over[MeSH])))) AND cancer)) AND Traveler’s Diarrhea))NOT (((((diarrhea AND morbidity AND ((“2014/01/01”[PDat] : “2021/04/30”[PDat])AND (Humans[Mesh]) AND (Clinical Trial[ptyp] OR Meta- Analysis[ptyp]OR Randomized Controlled Trial[ptyp] OR Review[ptyp] OR Clinical Trial,Phase I[ptyp] OR Clinical Trial, Phase II[ptyp] OR Clinical Trial, Phase III[ptyp]OR Clinical Trial, Phase IV[ptyp] OR Comparative Study[ptyp] OR Con-trolled Clinical Trial[ptyp] OR Corrected and Republished Article[ptyp] OREnglish Abstract[ptyp] OR Evaluation Studies[ptyp] OR Journal Article[ptyp]OR Multicenter Study[ptyp] OR Research Support, N I H, Extramural[ptyp]OR Research Support, N I H, Intramural[ptyp] OR Research Support, NonU S Gov’t[ptyp] OR Research Support, U S Gov’t, Non P H S[ptyp] ORResearch Support, U S Gov’t, P H S[ptyp] OR Twin Study[ptyp] OR Val-idation Studies[ptyp]) AND (child[MeSH:noexp] OR adolescent[MeSH] ORadult[MeSH:noexp] OR middle age[MeSH] OR (middle age[MeSH] OR aged[MeSH]) OR aged[MeSH] OR aged, 80 and over[MeSH]))) OR (diarrhea AND mortal-ity AND ((“2014/01/01”[PDat] : “2021/04/30”[PDat]) AND (Humans[Mesh])AND (Clinical Trial[ptyp] OR Meta-Analysis[ptyp] OR Randomized Con-trolled Trial[ptyp] OR Review[ptyp] OR Clinical Trial, Phase I[ptyp] OR Clin-ical Trial, Phase II[ptyp] OR Clinical Trial, Phase III[ptyp] OR Clinical Trial,Phase IV[ptyp] OR Comparative Study[ptyp] OR Controlled Clinical Trial[ptyp]OR Corrected and Republished Article[ptyp] OR English Abstract[ptyp] OREvaluation Studies[ptyp] OR Journal Article[ptyp] OR Multicenter Study[ptyp]OR Research Support, N I H, Extramural[ptyp] OR Research Support, NI H, Intramural[ptyp] OR Research Support, Non U S Gov’t[ptyp] OR Re-search Support, U S Gov’t, Non P H S[ptyp] OR Research Support, U SGov’t, P H S[ptyp] OR Twin Study[ptyp] OR Validation Studies[ptyp]) AND(child[MeSH:noexp] OR adolescent[MeSH] OR adult[MeSH:noexp] OR mid-dle age[MeSH] OR (middle age[MeSH] OR aged[MeSH]) OR aged[MeSH]OR aged, 80 and over[MeSH]))) OR (diarrhea AND etiology AND ((“2014/01/01”[PDat]: “2021/04/30”[PDat]) AND (Humans[Mesh]) AND (Clinical Trial[ptyp] ORMeta-Analysis[ptyp] OR Randomized Controlled Trial[ptyp] OR Review[ptyp]OR Clinical Trial, Phase I[ptyp] OR Clinical Trial, Phase II[ptyp] OR Clini-cal Trial, Phase III[ptyp] OR Clinical Trial, Phase IV[ptyp] OR ComparativeStudy[ptyp] OR Controlled Clinical Trial[ptyp] OR Corrected and Repub-lished Article[ptyp] OR English Abstract[ptyp] OR Evaluation Studies[ptyp]OR Journal Article[ptyp] OR Multicenter Study[ptyp] OR Research Support,N I H, Extramural[ptyp] OR Research Support, N I H, Intramural[ptyp] ORResearch Support, Non U S Gov’t[ptyp] OR Research Support, U S Gov’t,Non P H S[ptyp] OR Research Support, U S Gov’t, P H S[ptyp] OR TwinStudy[ptyp] OR Validation Studies[ptyp]) AND (child[MeSH:noexp] OR ado-lescent[MeSH] OR adult[MeSH:noexp] OR middle age[MeSH] OR (middleage[MeSH] OR aged[MeSH]) OR aged[MeSH] OR aged, 80 and over[MeSH]))))NOT (((diarrhea AND morbidity AND ((“2014/01/01”[PDat] : “2021/04/30”[PDat])AND (Humans[Mesh]) AND (Clinical Trial[ptyp] OR Meta-Analysis[ptyp]OR Randomized Controlled Trial[ptyp] OR Review[ptyp] OR Clinical Trial,Phase I[ptyp] OR Clinical Trial, Phase II[ptyp] OR Clinical Trial, Phase III[ptyp]OR Clinical Trial, Phase IV[ptyp] OR Comparative Study[ptyp] OR Con-trolled Clinical Trial[ptyp] OR Corrected and Republished Article[ptyp] OREnglish Abstract[ptyp] OR Evaluation Studies[ptyp] OR Journal Article[ptyp]OR Multicenter Study[ptyp] OR Research Support, N I H, Extramural[ptyp]OR Research Support, N I H, Intramural[ptyp] OR Research Support, NonU S Gov’t[ptyp] OR Research Support, U S Gov’t, Non P H S[ptyp] OR Research Support, U S Gov’t, P H S[ptyp] OR Twin Study[ptyp] OR Val-idation Studies[ptyp]) AND (child[MeSH:noexp] OR adolescent[MeSH] ORadult[MeSH:noexp] OR middle age[MeSH] OR (middle age[MeSH] OR aged[MeSH])OR aged[MeSH] OR aged, 80 and over[MeSH]))) OR (diarrhea AND mortal-ity AND ((“2014/01/01”[PDat] : “2021/04/30”[PDat]) AND (Humans[Mesh])AND (Clinical Trial[ptyp] OR Meta-Analysis[ptyp] OR Randomized Con-trolled Trial[ptyp] OR Review[ptyp] OR Clinical Trial, Phase I[ptyp] OR Clin-ical Trial, Phase II[ptyp] OR Clinical Trial, Phase III[ptyp] OR Clinical Trial,Phase IV[ptyp] OR Comparative Study[ptyp] OR Controlled Clinical Trial[ptyp]OR Corrected and Republished Article[ptyp] OR English Abstract[ptyp] OREvaluation Studies[ptyp] OR Journal Article[ptyp] OR Multicenter Study[ptyp]OR Research Support, N I H, Extramural[ptyp] OR Research Support, NI H, Intramural[ptyp] OR Research Support, Non U S Gov’t[ptyp] OR Re-search Support, U S Gov’t, Non P H S[ptyp] OR Research Support, U SGov’t, P H S[ptyp] OR Twin Study[ptyp] OR Validation Studies[ptyp]) AND(child[MeSH:noexp] OR adolescent[MeSH] OR adult[MeSH:noexp] OR mid-dle age[MeSH] OR (middle age[MeSH] OR aged[MeSH]) OR aged[MeSH]OR aged, 80 and over[MeSH]))) OR (diarrhea AND etiology AND ((“2014/01/01”[PDat]: “2021/04/30”[PDat]) AND (Humans[Mesh]) AND (Clinical Trial[ptyp] ORMeta-Analysis[ptyp] OR Randomized Controlled Trial[ptyp] OR Review[ptyp]OR Clinical Trial, Phase I[ptyp] OR Clinical Trial, Phase II[ptyp] OR Clini-cal Trial, Phase III[ptyp] OR Clinical Trial, Phase IV[ptyp] OR ComparativeStudy[ptyp] OR Controlled Clinical Trial[ptyp] OR Corrected and Repub-lished Article[ptyp] OR English Abstract[ptyp] OR Evaluation Studies[ptyp]OR Journal Article[ptyp] OR Multicenter Study[ptyp] OR Research Support,N I H, Extramural[ptyp] OR Research Support, N I H, Intramural[ptyp] ORResearch Support, Non U S Gov’t[ptyp] OR Research Support, U S Gov’t,Non P H S[ptyp] OR Research Support, U S Gov’t, P H S[ptyp] OR TwinStudy[ptyp] OR Validation Studies[ptyp]) AND (child[MeSH:noexp] OR ado-lescent[MeSH] OR adult[MeSH:noexp] OR middle age[MeSH] OR (middleage[MeSH] OR aged[MeSH]) OR aged[MeSH] OR aged, 80 and over[MeSH]))))AND cancer)) NOT ((((diarrhea AND morbidity AND ((“2014/01/01”[PDat]: “2021/04/30”[PDat]) AND (Humans[Mesh]) AND (Clinical Trial[ptyp] ORMeta-Analysis[ptyp] OR Randomized Controlled Trial[ptyp] OR Review[ptyp]OR Clinical Trial, Phase I[ptyp] OR Clinical Trial, Phase II[ptyp] OR Clini-cal Trial, Phase III[ptyp] OR Clinical Trial, Phase IV[ptyp] OR ComparativeStudy[ptyp] OR Controlled Clinical Trial[ptyp] OR Corrected and Repub-lished Article[ptyp] OR English Abstract[ptyp] OR Evaluation Studies[ptyp]

Appendix A. Appendix A: Search strings69OR Journal Article[ptyp] OR Multicenter Study[ptyp] OR Research Support,N I H, Extramural[ptyp] OR Research Support, N I H, Intramural[ptyp] ORResearch Support, Non U S Gov’t[ptyp] OR Research Support, U S Gov’t,Non P H S[ptyp] OR Research Support, U S Gov’t, P H S[ptyp] OR TwinStudy[ptyp] OR Validation Studies[ptyp]) AND (child[MeSH:noexp] OR ado-lescent[MeSH] OR adult[MeSH:noexp] OR middle age[MeSH] OR (middleage[MeSH] OR aged[MeSH]) OR aged[MeSH] OR aged, 80 and over[MeSH])))OR (diarrhea AND mortality AND ((“2014/01/01”[PDat] : “2021/04/30”[PDat])AND (Humans[Mesh]) AND (Clinical Trial[ptyp] OR Meta-Analysis[ptyp]OR Randomized Controlled Trial[ptyp] OR Review[ptyp] OR Clinical Trial,Phase I[ptyp] OR Clinical Trial, Phase II[ptyp] OR Clinical Trial, Phase III[ptyp]OR Clinical Trial, Phase IV[ptyp] OR Comparative Study[ptyp] OR Con-trolled Clinical Trial[ptyp] OR Corrected and Republished Article[ptyp] OREnglish Abstract[ptyp] OR Evaluation Studies[ptyp] OR Journal Article[ptyp]OR Multicenter Study[ptyp] OR Research Support, N I H, Extramural[ptyp]OR Research Support, N I H, Intramural[ptyp] OR Research Support, NonU S Gov’t[ptyp] OR Research Support, U S Gov’t, Non P H S[ptyp] ORResearch Support, U S Gov’t, P H S[ptyp] OR Twin Study[ptyp] OR Val-idation Studies[ptyp]) AND (child[MeSH:noexp] OR adolescent[MeSH] ORadult[MeSH:noexp] OR middle age[MeSH] OR (middle age[MeSH] OR aged[MeSH])OR aged[MeSH] OR aged, 80 and over[MeSH]))) OR (diarrhea AND etiologyAND ((“2014/01/01”[PDat] : “2021/04/30”[PDat]) AND (Humans[Mesh])AND (Clinical Trial[ptyp] OR Meta-Analysis[ptyp] OR Randomized Con-trolled Trial[ptyp] OR Review[ptyp] OR Clinical Trial, Phase I[ptyp] OR Clin-ical Trial, Phase II[ptyp] OR Clinical Trial, Phase III[ptyp] OR Clinical Trial,Phase IV[ptyp] OR Comparative Study[ptyp] OR Controlled Clinical Trial[ptyp]OR Corrected and Republished Article[ptyp] OR English Abstract[ptyp] OREvaluation Studies[ptyp] OR Journal Article[ptyp] OR Multicenter Study[ptyp]OR Research Support, N I H, Extramural[ptyp] OR Research Support, NI H, Intramural[ptyp] OR Research Support, Non U S Gov’t[ptyp] OR Re-search Support, U S Gov’t, Non P H S[ptyp] OR Research Support, U SGov’t, P H S[ptyp] OR Twin Study[ptyp] OR Validation Studies[ptyp]) AND(child[MeSH:noexp] OR adolescent[MeSH] OR adult[MeSH:noexp] OR mid-dle age[MeSH] OR (middle age[MeSH] OR aged[MeSH]) OR aged[MeSH]OR aged, 80 and over[MeSH])))) NOT (((diarrhea AND morbidity AND ((“2014/01/01”[PDat]: “2021/04/30”[PDat]) AND (Humans[Mesh]) AND (Clinical Trial[ptyp] ORMeta-Analysis[ptyp] OR Randomized Controlled Trial[ptyp] OR Review[ptyp]

Appendix A. Appendix A: Search strings70OR Clinical Trial, Phase I[ptyp] OR Clinical Trial, Phase II[ptyp] OR Clini-cal Trial, Phase III[ptyp] OR Clinical Trial, Phase IV[ptyp] OR ComparativeStudy[ptyp] OR Controlled Clinical Trial[ptyp] OR Corrected and Repub-lished Article[ptyp] OR English Abstract[ptyp] OR Evaluation Studies[ptyp]OR Journal Article[ptyp] OR Multicenter Study[ptyp] OR Research Support,N I H, Extramural[ptyp] OR Research Support, N I H, Intramural[ptyp] ORResearch Support, Non U S Gov’t[ptyp] OR Research Support, U S Gov’t,Non P H S[ptyp] OR Research Support, U S Gov’t, P H S[ptyp] OR TwinStudy[ptyp] OR Validation Studies[ptyp]) AND (child[MeSH:noexp] OR ado-lescent[MeSH] OR adult[MeSH:noexp] OR middle age[MeSH] OR (middleage[MeSH] OR aged[MeSH]) OR aged[MeSH] OR aged, 80 and over[MeSH])))OR (diarrhea AND mortality AND ((“2014/01/01”[PDat] : “2021/04/30”[PDat])AND (Humans[Mesh]) AND (Clinical Trial[ptyp] OR Meta-Analysis[ptyp]OR Randomized Controlled Trial[ptyp] OR Review[ptyp] OR Clinical Trial,Phase I[ptyp] OR Clinical Trial, Phase II[ptyp] OR Clinical Trial, Phase III[ptyp]OR Clinical Trial, Phase IV[ptyp] OR Comparative Study[ptyp] OR Con-trolled Clinical Trial[ptyp] OR Corrected and Republished Article[ptyp] OREnglish Abstract[ptyp] OR Evaluation Studies[ptyp] OR Journal Article[ptyp]OR Multicenter Study[ptyp] OR Research Support, N I H, Extramural[ptyp]OR Research Support, N I H, Intramural[ptyp] OR Research Support, NonU S Gov’t[ptyp] OR Research Support, U S Gov’t, Non P H S[ptyp] ORResearch Support, U S Gov’t, P H S[ptyp] OR Twin Study[ptyp] OR Val-idation Studies[ptyp]) AND (child[MeSH:noexp] OR adolescent[MeSH] ORadult[MeSH:noexp] OR middle age[MeSH] OR (middle age[MeSH] OR aged[MeSH])OR aged[MeSH] OR aged, 80 and over[MeSH]))) OR (diarrhea AND etiologyAND ((“2014/01/01”[PDat] : “2021/04/30”[PDat]) AND (Humans[Mesh])AND (Clinical Trial[ptyp] OR Meta-Analysis[ptyp] OR Randomized Con-trolled Trial[ptyp] OR Review[ptyp] OR Clinical Trial, Phase I[ptyp] OR Clin-ical Trial, Phase II[ptyp] OR Clinical Trial, Phase III[ptyp] OR Clinical Trial,Phase IV[ptyp] OR Comparative Study[ptyp] OR Controlled Clinical Trial[ptyp]OR Corrected and Republished Article[ptyp] OR English Abstract[ptyp] OREvaluation Studies[ptyp] OR Journal Article[ptyp] OR Multicenter Study[ptyp]OR Research Support, N I H, Extramural[ptyp] OR Research Support, NI H, Intramural[ptyp] OR Research Support, Non U S Gov’t[ptyp] OR Re-search Support, U S Gov’t, Non P H S[ptyp] OR Research Support, U SGov’t, P H S[ptyp] OR Twin Study[ptyp] OR Validation Studies[ptyp]) AND(child[MeSH:noexp] OR adolescent[MeSH] OR adult[MeSH:noexp] OR mid-dle age[MeSH] OR (middle age[MeSH] OR aged[MeSH]) OR aged[MeSH] OR aged, 80 and over[MeSH])))) AND cancer)) AND Traveler’s Diarrhea))AND Case report)

**Supplementary Search String 2**: Search String for SCOPUS

TITLE-ABS-KEY ( "diarrhea" ) OR TITLE-ABS-KEY("diarrhoea") AND ( TITLE-ABS-KEY ( "gastroenteritis" ) OR TITLE-ABS-KEY("mortality")) OR (TITLE-ABS-KEY ( "etiology" ) OR TITLE-ABS-KEY ( "pathogen" ) OR TITLE-ABS-KEY ( "incidence" ) OR TITLE-ABS-KEY ( "morbidity" ) OR TITLE-ABS-KEY( "cause of death" ) OR TITLE-ABS-KEY("diarrhea")) AND PUBYEAR > 2013AND NOT DOCTYPE ( cp ) AND NOT DOCTYPE ( ed ) AND NOT DOC-TYPE ( le ) AND NOT DOCTYPE ( no ) AND KEY ("human") AND NOTPUBYEAR = 2022 AND NOT TITLE-ABS-KEY ( "Case report" ) AND NOT TITLE-ABS-KEY ( "hospitalized acquired diarrhea" ) AND NOT TITLE-ABS-KEY ( "antibiotic associated diarrhea" ) AND NOT TITLE-ABS-KEY ( "cancerpatient" ) AND NOT TITLE-ABS-KEY ( "traveler" ) AND NOT TITLE-ABS-KEY ("refugees") AND NOT TITLE-ABS-KEY ("migrants") OR TITLE-ABS-KEY ("prospective study") AND TITLE-ABS-KEY ( diarrhea AND sample*) AND TITLE-ABS-KEY ( diarrhea AND agent ) AND NOT ( ABS ( "chil-dren under 5" ) OR ABS ( "children below 5") OR ABS ("children < 5" )OR ABS ("children under five") OR ABS ("children below five") ) OR ABS( daycare* AND outbreak* ) OR ABS ( adult AND care* AND outbreak*) AND NOT ( ABS ( recall AND period AND below 4 week* ) OR ABS( recall AND period AND under 4 week* ) OR ABS ( recall AND periodAND below AND four AND week* ) OR ABS ( recall AND period ANDunder AND four AND week* ) ) AND NOT TITLE-ABS-KEY("cross sec-tional study") AND NOT TITLE-ABS-KEY("animal") AND NOT TITLE-ABS-KEY("neonatal") AND NOT TITLE-ABS-KEY("COVID-19") AND NOT TITLE-ABS-KEY("infant") AND NOT TITLE-ABS-KEY("SARS-CoV-2") AND NOTTITLE-ABS-KEY("cancer") AND NOT TITLE-ABS-KEY("HIV mortality") ANDNOT TITLE-ABS-KEY("diabetes") AND NOT TITLE-ABS-KEY("dysentery")AND NOT TITLE-ABS-KEY("under-five") AND NOT TITLE-ABS-KEY("childbirth")AND NOT TITLE-ABS-KEY("animal") AND ( LIMIT-TO ( AFFILCOUNTRY,"SouthAfrica" ) OR LIMIT-TO ( AFFILCOUNTRY,"Egypt" ) OR LIMIT-TO ( AF-FILCOUNTRY,"Nigeria" ) OR LIMIT-TO ( AFFILCOUNTRY,"Ethiopia" ) ORLIMIT-TO ( AFFILCOUNTRY,"Kenya" ) OR LIMIT-TO ( AFFILCOUNTRY,"Uganda") OR LIMIT-TO ( AFFILCOUNTRY,"Ghana" ) OR LIMIT-TO ( AFFILCOUN-TRY,"Tunisia" ) OR LIMIT-TO ( AFFILCOUNTRY,"Morocco" ) OR LIMIT-TO ( AFFILCOUNTRY,"Cameroon" ) OR LIMIT-TO ( AFFILCOUNTRY,"Malawi") OR LIMIT-TO ( AFFILCOUNTRY,"Algeria" ) OR LIMIT-TO ( AFFILCOUN-TRY,"Zambia" ) OR LIMIT-TO ( AFFILCOUNTRY,"Zimbabwe" ) OR LIMIT-TO ( AFFILCOUNTRY,"Burkina Faso" ) OR LIMIT-TO ( AFFILCOUNTRY,"Sudan") OR LIMIT-TO ( AFFILCOUNTRY,"Senegal" ) OR LIMIT-TO ( AFFILCOUN-TRY,"Mozambique" ) OR LIMIT-TO ( AFFILCOUNTRY,"Congo" ) OR LIMIT-TO ( AFFILCOUNTRY,"Rwanda" ) OR LIMIT-TO ( AFFILCOUNTRY,"Coted’Ivoire" ) OR LIMIT-TO ( AFFILCOUNTRY,"Benin" ) OR LIMIT-TO ( AFFIL-COUNTRY,"Botswana" ) OR LIMIT-TO ( AFFILCOUNTRY,"Gambia" ) ORLIMIT-TO ( AFFILCOUNTRY,"Mali" ) OR LIMIT-TO ( AFFILCOUNTRY,"Madagascar") OR LIMIT-TO ( AFFILCOUNTRY,"Democratic Republic Congo" ) OR LIMIT-TO ( AFFILCOUNTRY,"Sierra Leone" ) OR LIMIT-TO ( AFFILCOUNTRY,"Namibia") OR LIMIT-TO ( AFFILCOUNTRY,"Libyan Arab Jamahiriya" ) OR LIMIT-TO ( AFFILCOUNTRY,"Gabon" ) OR LIMIT-TO ( AFFILCOUNTRY,"Togo" )OR LIMIT-TO ( AFFILCOUNTRY,"Guinea-Bissau" ) OR LIMIT-TO ( AFFIL-COUNTRY,"Niger" ) OR LIMIT-TO ( AFFILCOUNTRY,"Liberia" ) OR LIMIT-TO ( AFFILCOUNTRY,"Angola" ) )

# **Supplementary Table S1:** Search String for Web of Science

| Search terms and combinations (≠1 OR ≠2 OR ≠3 OR ≠4) | | | |
| --- | --- | --- | --- |
| ≠1 | Diarrhea* OR diarrhoea* | AND | incid* OR occur* |
| ≠2 | Diarrhea* OR diarrhoea* | AND | mortal* OR gastroent* |
| ≠3 | Diarrhea* OR diarrhoea* | AND | etiol* OR aetiol* OR pathog* OR cause of death |
| ≠4 | Diarrhea* OR diarrhoea* | AND | child* OR young* OR adolescent* OR infant* |

# **Supplementary Table S2:** Characteristics of the studies included in the review (n = 38).

|  | **Year(s) of study** | **Country** | **African Region** | **Area Classification** | **Type of study** | **Study design** | **Age Group** | **Diagnostic method(s)** | **Total number of pathogens investigated** | **Pathogens investigated (that are relevant for this study)** | **Rotavirus vaccine introduced (as of study introduction)** |
| --- | --- | --- | --- | --- | --- | --- | --- | --- | --- | --- | --- |
| Ouédraogo et al (2016) | 2011-2012 | Burkina Faso | Western Africa | Urban | Out-patient | Prospective case-control | 0-5 years | PCR (end-point or RT) | 5 | Astrovirus, rotavirus, norovirus | No |
| Japhet et al (2019) | 2012-2013 | Nigeria | Western Africa | Mixed | Out-patient | Case-control | 0-5 years | PCR | 5 | Rotavirus, norovirus, astrovirus | No |
| Arowolo et al (2019) | 2015-2017 | Nigeria | Western Africa | Urban | In-patient | Cross-sectional | 0-5 years | PCR | 4 | Rotavirus, norovirus, astrovirus | No |
| Gasparinho et al (2016) | 2012-2013 | Angola | Central Africa | Urban | In-patient and out-patient | Cross-sectional | 0-5 years | Antigen Rapid Test, ELISA, PCR, Microscopy, Culture | 15 | *Cryptosporidium* spp., rotavirus, *G. lamblia*, EAEC, ETEC, astrovirus, *E. histolytica* | No |
| Hungerford et al (2020) | 2012-2015 | Malawi | Southern Africa | Unknown | Community setting | Case-control | 0-5 years | RT-PCR | 29 | *Campylobacter* spp., *Cryptosporidium* spp., *G. lamblia*, rotavirus, astrovirus, norovirus | Yes |
| Acacio et al (2019) | 2007-2012 | Mozambique | Eastern Africa | Urban | Out-patient, in-patient | Case-control | 0-5 years | Serological screening, PCR | 26 | ETEC, EAEC, EPEC, *Shigella* spp., rotavirus, norovirus, *V*. *cholerae*, *Salmonella* spp., *Cryptosporidium* spp., *G*. *lamblia*, *E. histolytica*, *Campylobacter* spp., astrovirus | No |
| Nhampossa et al (2015) | 2007-2011 | Mozambique | Eastern Africa | Rural | In-patient, out-patient, and community study | Prospective case-control | 0-5 years | Multiplex PCR, RT-PCR, ELISA, Immunoassays | 21 | *G. lamblia*, *Cryptosporidium* spp., *E. histolytica*, rotavirus, norovirus, astrovirus, ETEC, EAEC, EPEC, *Shigella* spp., *Salmonella* spp., *V. cholerae*, *Campylobacter* spp. | No |
| Iturriza-Gómara et al (2019) | 2013-2016 | Malawi | Southern Africa | Urban | Community setting, in-patient | Case-control | 0-5 years | RT-PCR | 31 | EAEC, rotavirus, *Cryptosporidium* spp., ETEC, EPEC, *Campylobacter* spp., EIEC, norovirus, *G. lamblia*, *Salmonella* spp., astrovirus, *E. histolytica*, *V. cholerae*, STEC | Yes |
| Platts-Mills et al (2015) | 2009-2012 | South Africa | Southern Africa | Unknown | Community-setting | Cohort study | 0-2 years | Multiplex PCR | 25 | *Campylobacter* spp., *G. lamblia*, EAEC, norovirus, ETEC, astrovirus, rotavirus, EPEC, EPEC, EIEC, STEC, *V. cholerae* | Yes |
| Zimmermann et al (2019) | 2007-2011 | Mali, Mozambique, Kenya, The Gambia | Western Africa, Eastern Africa | Unknown | In-patient and out-patient | Retrospective cohort study | 0-5 years | Serological screening, PCR | 7 | *Shigella* spp., *Campylobacter* spp., *Cryptosporidium* spp., norovirus, rotavirus, ETEC, *V. cholerae* | No |
| Mayindou et al (2019) | 2012-2013 | Democratic Republic of Congo | Central Africa | Urban | In-patient | Cross-sectional | 0-5 years | ELISA, RT-PCR | 2 | Rotavirus, norovirus | No |
| Chuckwu et al (2020) | 2016-2017 | South Africa | Southern Africa | Mixed | Community setting | Cross-sectional | 0-5 years | RT-PCR | 5 | *Campylobacter*, rotavirus, norovirus | Yes |
| El Qazoui et al (2014) | 2011 | Morocco | Northern Africa | Urban | In-patient | Cross-sectional | 0-5 years | RT-multiplex PCR, RT-PCR | 2 | Rotavirus, norovirus | Yes |
| Ashie et al (2017) | 2012-2014 | Ghana | Western Africa | Mixed | Out-patient, in-patient | Case-control | 0-5 years | Microscopy, immunoassays | 8 | Rotavirus, *Shigella* spp., *Salmonella* spp., *G. lamblia* | Yes |
| Msolo et al (2020) | 2017-2018 | South Africa | Southern Africa | Rural | In-patient | Cross-sectional | 0-40 years | Immunoassays | 2 | Rotavirus, *Cryptosporidium* spp. | Yes |
| El-Shabrawi et al (2015) | 2007-2009 | Egypt | Northern Africa | Urban | Out-patient | Case-control | 0-5 years | ELISA | 6 | ETEC, *Campylobacter* spp., *Shigella* spp., *Salmonella* spp., *Cryptosporidium* spp., rotavirus | No |
| Mansour et al (2014) | 2004-2007 | Egypt | Northern Africa | Rural | Community setting | Cohort study | 0-2 years | ELISA | 4 | ETEC, *Campylobacter* spp., *Shigella* spp., rotavirus | No |
| Gosselin et al (2018) | 2009-2011 | Tanzania | Eastern Africa | Urban | Community setting | Prospective randomized trial | 0-2 years | PCR | 15 | *Cryptosporidium* spp., rotavirus, *Shigella* spp., EIEC, *Campylobacter* spp., EPEC, ETEC | No |
| Imade et al (2015) | 2011-2012 | Nigeria | Western Africa | Mixed | Out-patient | Cross-sectional | 0-3 years | Immunoassays | 3 | Rotavirus, norovirus | No |
| Lompo et al (2021) | 2012-2014 | Burkina Faso | Western Africa | Rural | In-patient | Cross-sectional | 0-5 years | Immunoassay | 6 | EPEC, *G. lamblia*, *Shigella* spp., rotavirus | Yes |
| Onanuga et al (2014) | 2008-2009 | Nigeria | Western Africa | Urban | Out-patient | Prospective cohort | 0-5 years | PCR | 1^*^ | EPEC, ETEC, EHEC, EAEC, EIEC, STEC | - |
| Odetoyin et al (2016) | 2008-2011 | Nigeria | Western Africa | Unknown | Community setting | Cross-sectional | 0-46 years | Multiplex PCR | 1^*^ | EPEC, EAEC, ETEC, STEC, EHEC | - |
| Lijima et al (2017) | 2007-2009 | Kenya | Eastern Africa | Urban | Out-patient | Cross-sectional | 0-5 years | RT-PCR | 1^*^ | EPEC, EAEC, ETEC, STEC, EHEC, EIEC | - |
| Hassan et al (2014) | 2004-2007 | Egypt | Northern Africa | Rural | Community setting | Prospective cohort | 0-2 years | ELISA | 4 | *Shigella* spp., *Campylobacter* spp., ETEC | - |
| Njuguna et al (2016) | 2012 | Kenya | Eastern Africa | Mixed | In-patient and out-patient | Matched case-control | All ages | Microscopy, PCR | 9 | *Shigella* spp., *Salmonella* spp., *G. lamblia*, *E. histolytica* | - |
| Terfassa, Jida (2018) | 2015-2016 | Ethiopia | Eastern Africa | Rural | Out-patient | Cross-sectional | All ages | Immunoassay | 2 | *Salmonella* spp., *Shigella* spp. | - |
| Ferreira et al (2020) | 2012-2013 | Mozambique | Eastern Africa | Urban | In-patient | Cross-sectional | 0-60 years | Microscopy | 10 | *E*. *histolytica*, *Cryptosporidium* spp. | - |
| Bauhofer et al (2020) | 2014-2018 | Mozambique | Eastern Africa | Urban | In-patient and out-patient | Cross-sectional | 0-14 years | Immunoassay | 3 | *Cryptosporidium* spp., *G. lamblia*, *E. histolytica* | - |
| von Huth et al (2019) | 2015-2017 | Guinea-Bissau | Western Africa | Urban | Community setting | Cohort | 2-7 years | Microscopy | 14 | *G. lamblia*, *E. histolytica* | - |
| Garzón et al (2017) | 2013-2015 | Sáo Tomé and Príncipe | Western Africa | Mixed | Community setting | Cross-sectional | 0-5 years | Microscopy, ELISA, PCR | 8 | *G. lamblia*, *Cryptosporidium* spp., *E. histolytica* | - |
| Muadica et al (2021) | 2017-2019 | Mozambique | Eastern Africa | Mixed | Community setting | Prospective cross-sectional | 3-14 years | PCR | 3 | *Cryptosporidium* spp. | - |
| Ramos et al (2014) | 2007-2012 | Ethiopia | Eastern Africa | Rural | In-patient | Retrospective observational study | All ages | Microscopy | 9 | *G*. *lamblia*, *E*. *histolytica* | - |
| Tellevik et al (2015) | 2010-2011 | Tanzania | Eastern Africa | Urban | Out-patient | Case-control | 0-5 years | PCR | 2 | *G*. *lamblia*, *E*. *histolytica* | - |
| Trainor et al (2016) | 1997-2007 | Malawi | Southern Africa | Urban | In-patient | Cohort | 0-5 years | PCR | 4 | ETEC | - |
| Prah et al (2021) | 2016-2018 | Ghana | Western Africa | Unknown | Out-patient | Prospective case-control | 0-5 years | PCR | 1^*^ | EIEC, EHEC, EPEC, STEC, ETEC, EAEC | - |
| Omolajaiye et al (2020) | 2015-2017 | South Africa | Southern Africa | Unknown | In-patient | Prospective cross-sectional | 0-12 years | PCR | 1^*^ | EHEC, EIEC, EPEC | - |
| Naguib et al (2018) | 2015-2016 | Egypt | Northern Africa | Mixed | Community setting | Cross-sectional | 0-8 years | PCR-RFLP | 2 | *Cryptosporidium* spp. | - |
| Bitilinyu-Bangoh et al (2019) | 2014-2015 | Malawi, Kenya | Southern Africa, Eastern Africa | Unknown | In-patient | Randomised controlled trial | 0-5 years | Multiplex PCR | 3 | *E*. *histolytica*, *Cryptosporidium* spp. | - |

**^*^**The study passed the inclusion criteria if it presented more than one type of diarrheagenic *E. coli*.

# **Supplementary Table S3:** Detailed description of the studies reporting incidence of diarrhea meeting inclusion criteria, stratified by age group and/or study setting

|  | **Pathogens etiologies reported** | **Sub-group specification** | **Total population (t)** | **Rotavirus (n)** | **Norovirus (n)** | **Astrovirus (n)** | ***Cryptosporidium* spp. (n)** | ***E. histolytica* (n)** | ***G. lamblia* (n)** | ***Campylobacter* spp.** | **EAEC (n)** | **EPEC (n)** | **EHEC (n)** | **ETEC (n)** | **EIEC (n)** | **STEC (n)** | ***Salmonella* spp. (n)** | ***Shigella* spp. (n)** | ***V*. *cholerae* (n)** | ***Other (n)*** | ***Unknown (n)*** |
| --- | --- | --- | --- | --- | --- | --- | --- | --- | --- | --- | --- | --- | --- | --- | --- | --- | --- | --- | --- | --- | --- |
| Ouédraogo et al (2016) | Astrovirus, rotavirus, norovirus | - | 263 | 167 | 48 | 13 | - | - | - | - | - | - | - | - | - | - | - | - | - | 111 | - |
| Japhet et al (2019) | Astrovirus, rotavirus, norovirus | 0-5 months | 21 | 20 | 1 | 0 | - | - | - | - | - | - | - | - | - | - | - | - | - | - | - |
|  |  | 6-11 months | 21 | 14 | 6 | 1 | - | - | - | - | - | - | - | - | - | - | - | - | - | - | - |
|  |  | 12-23 months | 9 | 6 | 0 | 3 | - | - | - | - | - | - | - | - | - | - | - | - | - | - | - |
|  |  | 24-35 months | 1 | 1 | 0 | 0 | - | - | - | - | - | - | - | - | - | - | - | - | - | - | - |
|  |  | All ages (0-35 month) | 103 | - | - | - | - | - | - | - | - | - | - | - | - | - | - | - | - | 1 | - |
| Zimmermann et al (2019 | *Shigella* spp., *Campylobacter* spp., *Cryptosporidium* spp., norovirus, rotavirus, ETEC, *V*. *cholerae* | The Gambia | 1933 | 318 (16.5%) | 189 (9.8%) | - | 233 (12.1%) | - | - | 39 (2%) | - | - | - | 220 (11.4%) | - | - | - | 162 (8.4%) | 0 (0%) | - | - |
|  |  | Kenya | 1778 | 252 (14.2%) | 89 (5%) | - | 196 (11%) | - | - | 171 (9.6%) | - | - | - | 176 (9.9%) | - | - | - | 130 (7.3%) | 7 (0.4%) | - | - |
|  |  | Mali | 3404 | 417 (12.3%) | 77 (2.3%) | - | 337 (9.9%) | - | - | 52 (1.5%) | - | - | - | 176 (5.2%) | - | - | - | 51 (1.5%) | 0 (0%) | - | - |
|  |  | Mozambique | 1217 | 342 (28.1%) | 29 (2.4%) | - | 193 (15.9%) | - | - | 22 (1.8%) | - | - | - | 119 (9.8%) | - | - | - | 57 (4.7%) | 12 (1%) | - | - |
| Arowolo et al (2019) | Rotavirus, astrovirus, norovirus | - | 175 | 29 | 9 | 34 | - | - | - | - | - | - | - | - | - | - | - | - | - | 9 | - |
| Gasparinho et al (2016) | *Cryptosporidium* spp., rotavirus, *G. lamblia*, EAEC, ETEC, astrovirus, *E. histolytica* | - | 342 (rotavirus), 337 (*Cryptosporidium* spp.), 338 (*G. lamblia*), 274 (astrovirus), 341 (*E. histolytica*), 301 (EAEC, ETEC), 344 (unknown) | 86 | - | 7 | 101 | 1 | 73 | - | 12 | - | - | 7 | - | - | - | - | - | 82 | 115 |
| Hungerford et al. (2014) | *Campylobacter* spp., *Cryptosporidium* spp., *G. lamblia*, rotavirus, astrovirus, norovirus | Out-patient | 527 | 0 (0%) | - | 58 (11%) | 11 (2%) | - | 105 (20%) | 190 (36%) | - | - | - | - | - | - | - | - | - | - | - |
|  |  | In-patient | 684 | 130 (19%) | - | 0 (0%) | 185 (27%) | - | 48 (7%) | 150 (22%) | - | - | - | - | - | - | - | - | - | - | - |
|  |  | In-patient (0-5 months) | 72 | - | 11 | - | - | - | - | - | - | - | - | - | - | - | - | - | - | - | - |
|  |  | Out-patient (0-5 months) | 30 | - | 3 | - | - | - | - | - | - | - | - | - | - | - | - | - | - | - | - |
|  |  | In-patient (6-11 months) | 331 | - | 44 | - | - | - | - | - | - | - | - | - | - | - | - | - | - | - | - |
|  |  | Out-patient (6-11 months) | 339 | - | 30 | - | - | - | - | - | - | - | - | - | - | - | - | - | - | - | - |
|  |  | In-patient (12-23 months) | 219 | - | 24 | - | - | - | - | - | - | - | - | - | - | - | - | - | - | - | - |
|  |  | Out-patient (12-23 months) | 192 | - | 8 | - | - | - | - | - | - | - | - | - | - | - | - | - | - | - | - |
|  |  | In-patient (24-60 months) | 61 | - | 4 | - | - | - | - | - | - | - | - | - | - | - | - | - | - | - | - |
|  |  | Out-patient (24-60 months) | 70 | - | 5 | - | - | - | - | - | - | - | - | - | - | - | - | - | - | - | - |
| Acacio et al (2019) | ETEC, EAEC, *Shigella* spp., rotavirus, norovirus, *V. cholerae*, *Salmonella* spp., *Cryptosporidium* spp., *G. lamblia*, *E*. *histolytica*, *Campylobacter* spp., astrovirus | In-patient | 14 (ETEC), 69 (EAEC, *V. cholerae*, *Shigella* spp., rotavirus, norovirus, astrovirus, *Cryptosporidium* spp., *E. histolytica*, *Campylobacter* spp., *G. lamblia*, *Salmonella* spp., Other) | 15 | 1 | 3 | 26 | 9 | 10 | 2 | 15 | - | - | 5 | - | - | 0 | 3 | 1 | 1 | - |
|  |  | Out-patient | 172 (ETEC), 752 (EAEC, *Shigella* spp., *Campylobacter* spp., *V.* *cholerae*, *Salmonella* spp.), 751 (rotavirus, norovirus, astrovirus, *Cryptosporidium* spp.,*G. lamblia*, Other), 750 (*E. histolytica*) | 251 | 30 | 10 | 121 | 75 | 133 | 35 | 210 | - | - | 46 | - | - | 7 | 43 | 12 | 92 | - |
| Nhampossa et al (2015) | *G. lamblia*, *Cryptosporidium* spp., *E*. *histolytica*, rotavirus, norovirus, astrovirus, ETEC, EAEC, EPEC, *Shigella* spp., *Salmonella* spp., *V. cholerae*, *Campylobacter* spp. | 0-11 months, in-patient | 431 | 182 | 19 | 7 | 84 | 39 | 41 | 24 | 150 | 43 | - | 20 | - | - | 6 | 6 | 4 | - | - |
|  |  | 0-11 months, out-patient | 861 | 139 | 38 | 11 | 86 | 79 | 152 | 0 | 273 | 67 | - | 19 | - | - | 6 | 1 | 1 | - | - |
|  |  | 12-23 months, in-patient | 233 | 52 | 10 | 6 | 44 | 26 | 64 | 9 | 60 | 17 | - | 29 | - | - | 2 | 18 | 9 | - | - |
|  |  | 12-23 months, out-patient | 502 | 91 | 25 | 10 | 46 | 52 | 228 | 14 | 92 | 35 | - | 16 | - | - | 0 | 2 | 0 | - | - |
|  |  | 24-60 months, in-patient | 120 | 12 | 4 | 1 | 11 | 15 | 42 | 0 | 21 | 5 | - | 8 | - | - | - | 20 | - | - | - |
|  |  | 24-60 months, out-patient | 232 | 27 | 12 | 4 | 18 | 28 | 115 | 2 | 35 | 13 | - | 12 | - | - | - | 0 | - | - | - |
|  |  | In-patient (All age-groups) | 431 | - | - | - | - | - | - | - | - | - | - | - | - | - | - | - | - | 25 | - |
|  |  | Out-patient (All age-groups) | 861 | - | - | - | - | - | - | - | - | - | - | - | - | - | - | - | - | 54 | - |
| Iturriza-Gómara et al (2019) | Rotavirus, *Cryptosporidium* spp., ETEC, EPEC, *Campylobacter* spp., norovirus, *G. lamblia*, *Salmonella* spp., astrovirus, *E. histolytica*, *V. cholerae*, STEC | In-patient | 684 | 237 | 83 | 12 | 190 | 10 | 50 | 113 | - | 123 | - | 145 | - | 5 | 30 | 108 | 9 | 368 | 43 |
|  |  | Out-patient | 527 | 8 | 45 | 13 | 43 | 1 | 73 | 102 | - | 45 | - | 45 | - | 1 | 5 | 30 | 0 | 121 | - |
| Platts-Mills et al (2015) | *Campylobacter* spp., *G. lamblia*, EAEC, norovirus, ETEC, astrovirus, rotavirus, EPEC, EPEC, EIEC, STEC, *V. cholerae* | 0-11 months | 84 | 2 (2.4%) | 44 (9.5%) | 2 (2.4%) | - | - | - | 24 (28.6%) | 22 (26.2%) | 3 (3.6%) | - | 5 (4.8%) | - | - | - | - | - | 3 (3.6%) | - |
|  |  | 12-24 months | 73 | 2 (2.7%) | 29 (39.7%) | 3 (4.1%) | - | - | 15 (20.5%) | 13 (17.8%) | 19 (26.0%) | 2 (2.7%) | - | - | - | 3 (4.1%) | - | - | - | 5 (6.8%) | - |
| Mayindou et al (2019) | Rotavirus | - | 655 | 226 | - | - | - | - | - | - | - | - | - | - | - | - | - | - | - | - | - |
| Chuckwu et al (2016) | *Campylobacter* spp., rotavirus, norovirus | - | 505 | 118 | 101 | - | - | - | - | 254 | - | - | - | - | - | - | - | - | - | 80 | - |
| El Qazoui et al (2014) | Rotavirus, norovirus | - | 335 | 89 | 42 | - | - | - | - | - | - | - | - | - | - | - | - | - | - | - | - |
| Ashie et al (2017) | Rotavirus, *Shigella* spp., *Salmonella* spp., *G. lamblia* | 0-12 months | 155 | 16 | - | - | - | - | - | - | - | - | - | - | - | - | 7 | 5 | - | - | - |
|  |  | 13-24 months | 132 | 42 | - | - | - | - | - | - | - | - | - | - | - | - | 2 | 8 | - | - | - |
|  |  | 25-60 months | 60 | 14 | - | - | - | - | - | - | - | - | - | - | - | - | 11 | 3 | - | - | - |
|  |  | 0-60 months (group 1) | 107 | - | - | - | - | - | 5 | - | - | - | - | - | - | - | - | - | - | 18 | - |
|  |  | 0-60 months (group 2) | 240 | - | - | - | - | - | 7 | - | - | - | - | - | - | - | - | - | - | 32 | - |
| Msolo et al (2020) | Rotavirus, *Cryptosporidium* spp. | - | 53 | 19 | - | - | 3 | - | - | - | - | - | - | - | - | - | - | - | - | - | - |
| El-Shabrawi et al (2015) | ETEC, *Campylobacter* spp., *Shigella* spp., *Salmonella* spp., *Cryptosporidium* spp., rotavirus | 0-6 months | 160 | 19 | - | - | 6 | - | - | 7 | - | - | - | 11 | - | - | 3 | 3 | - | - | - |
|  |  | 7-12 months | 105 | 13 | - | - | 6 | - | - | 2 | - | - | - | 5 | - | - | 1 | 3 | - | - | - |
|  |  | 13-24 months | 66 | 6 | - | - | 2 | - | - | 4 | - | - | - | 8 | - | - | 1 | 3 | - | - | - |
|  |  | 25-60 months | 25 | 0 | - | - | 0 | - | - | 0 | - | - | - | 1 | - | - | 0 | 1 | - | - | - |
| Mansour et al (2014) | ETEC, *Campylobacter* spp., *Shigella* spp., rotavirus | - | 4001 | 151 | - | - | - | - | - | 238 | - | - | - | 422 | - | - | - | 40 | - | - | - |
| Gosselin et al (2018) | *Cryptosporidium* spp., rotavirus, *Shigella* spp., EIEC, *Campylobacter* spp., EPEC, ETEC | - | 123 | 11 | - | - | 9 | - | - | 3 | - | 2 | - | 3 | - | - | - | 7 | - | 35 | 52 |
| Imade et al (2015) | Rotavirus, norovirus | 0-6 months | 63 | 20 | 32 | - | - | - | - | - | - | - | - | - | - | - | - | - | - | - | - |
|  |  | 7-12 months | 82 | 31 | 48 | - | - | - | - | - | - | - | - | - | - | - | - | - | - | - | - |
|  |  | 13-18 months | 35 | 7 | 8 | - | - | - | - | - | - | - | - | - | - | - | - | - | - | - | - |
|  |  | 19-24 months | 26 | 4 | 5 | - | - | - | - | - | - | - | - | - | - | - | - | - | - | - | - |
|  |  | 25-30 months | 11 | 1 | 2 | - | - | - | - | - | - | - | - | - | - | - | - | - | - | - | - |
|  |  | 31-36 months | 6 | 0 | 0 | - | - | - | - | - | - | - | - | - | - | - | - | - | - | - | - |
|  |  | 0-36 months | 223 | 63 | 8 | - | - | - | - | - | - | - | - | - | - | - | - | - | - | - | - |
| Lompo et al (2021) | EPEC, *G. lamblia*, *Shigella* spp., rotavirus | - | 191 | 64 (33⋅3%) | - | - | - | - | 87 (45⋅65%) | - | - | 11 (6%) | - | - | - | - | - | 15 (8%) | - | - | - |
| Onanuga et al (2014) | EPEC, ETEC, EHEC, EAEC, EIEC, STEC | - | 201 | - | - | - | - | - | - | - | 21 | 9 | - | 11 | 1 | 19 | - | - | - | - | - |
| Odetoyin et al (2016) | EPEC, EAEC, ETEC, STEC, EHEC | 0-6 months | 25 | - | - | - | - | - | - | - | 0 | 2 | 0 | 1 | - | 5 | - | - | - | - | - |
|  |  | 7-12 months | 57 | - | - | - | - | - | - | - | 4 | 4 | 3 | 11 | - | 10 | - | - | - | - | - |
|  |  | 13-24 months | 33 | - | - | - | - | - | - | - | 5 | 1 | 1 | 8 | - | 8 | - | - | - | - | - |
|  |  | 25-60 months | 11 | - | - | - | - | - | - | - | 1 | 0 | 0 | 0 | - | 1 | - | - | - | - | - |
|  |  | 15-46 years | 126 | - | - | - | - | - | - | - | 11 | 7 | - | 20 | 2 | 18 | - | - | - | - | - |
| Lijima et al (2017) | EPEC, EAEC, ETEC, STEC, EHEC, EIEC | - | 306 | - | - | - | - | - | - | - | 138 | 51 | - | 50 | 25 | 0 | - | - | - | - | - |
| Hassan et al (2014) | *Shigella* spp., *Campylobacter* spp., ETEC | - | 4001 | - | - | - | - | - | - | 240 | - | - | - | 632 | - | - | - | 40 | - | - | - |
| Njuguna et al (2016) | *Shigella* spp., *Salmonella* spp., *G. lamblia*, *E. histolytica* | In-patient | 284 | - | - | - | - | 31 | 6 | - | - | - | - | - | - | - | 3 | 67 | - | 3 | 169 |
|  |  | Out-patient | 114 | - | - | - | - | 1 | 1 | - | - | - | - | - | - | - | 0 | 1 | - | - | - |
| Terfassa, Jida (2018) | *Salmonella* spp., *Shigella* spp. | - | 422 | - | - | - | - | - | - | - | - | - | - | - | - | - | 30 | 9 | - | - | - |
| Ferreira et al (2020) | *Cryptosporidium* spp., *E. histolytica* | - | 831 | - | - | - | 28 | 3 | - | - | - | - | - | - | - | - | - | - | - | 233 | - |
| Bauhofer et al (2020) | *Cryptosporidium* spp., *G. lamblia*, *E. histolytica* | 0-11 months | 489 (*E. histolytica*), 478 (*Cryptosporidium*  spp.), 477 (*G. lamblia*) | - | - | - | 64 | 9 | 31 | - | - | - | - | - | - | - | - | - | - | - | - |
|  |  | 12-23 months | 337 (*E. histolytica*), 331 (*Cryptosporidium*  spp.), 330 (*G. lamblia*) | - | - | - | 42 | 6 | 40 | - | - | - | - | - | - | - | - | - | - | - | - |
|  |  | 24-59 months | 123 (*E. histolytica*), 121 *Cryptosporidium*  spp., *G. lamblia*) | - | - | - | 9 | 4 | 14 | - | - | - | - | - | - | - | - | - | - | - | - |
|  |  | 1-14 years | 55 | - | - | - | 3 | 1 | 10 | - | - | - | - | - | - | - | - | - | - | - | - |
| von Huth et al (2019) | *G. lamblia, E. histolytica* | 2-7 years (group 1) | 471 | - | - | - | - | 71 | 150 | - | - | - | - | - | - | - | - | - | - | - | - |
|  |  | 2-7 years (group 2) | 377 | - | - | - | - | 58 | 84 | - | - | - | - | - | - | - | - | - | - | - | - |
|  |  | 8-15 years (group 1) | 237 | - | - | - | - | 51 | 38 | - | - | - | - | - | - | - | - | - | - | - | - |
|  |  | 8-15 years (group 2) | 189 | - | - | - | - | 40 | 32 | - | - | - | - | - | - | - | - | - | - | - | - |
| Garzón et al (2017) | *G. lamblia, Cryptosporidium spp., E. histolytica* | 0-3 months | 74 | - | - | - | 0 | 0 | 0 | - | - | - | - | - | - | - | - | - | - | 0 | - |
|  |  | 4-6 months | 76 | - | - | - | 1 | 0 | 5 | - | - | - | - | - | - | - | - | - | - | 5 | - |
|  |  | 7-9 months | 52 | - | - | - | 3 | 0 | 5 | - | - | - | - | - | - | - | - | - | - | 5 | - |
|  |  | 10-12 months | 78 | - | - | - | 3 | 0 | 9 | - | - | - | - | - | - | - | - | - | - | 14 | - |
|  |  | 13-16 months | 71 | - | - | - | 2 | 0 | 16 | - | - | - | - | - | - | - | - | - | - | 27 | - |
|  |  | 17-18 months | 70 | - | - | - | 1 | 0 | 16 | - | - | - | - | - | - | - | - | - | - | 28 | - |
|  |  | 19-24 months | 80 | - | - | - | 3 | 0 | 21 | - | - | - | - | - | - | - | - | - | - | 31 | - |
| Muadica et al (2021) | *Cryptosporidium spp* | - | 286 | - | - | - | 2 | - | - | - | - | - | - | - | - | - | - | - | - | 126 | - |
| Ramos et al (2014) | *G. lamblia, E. histolytica* | <5 years | 6776 | - | - | - | - | 242 | 1132 | - | - | - | - | - | - | - | - | - | - | 574 | - |
|  |  | 5-9 years | 4601 | - | - | - | - | 262 | 810 | - | - | - | - | - | - | - | - | - | - | 623 | - |
|  |  | 10-14 years | 2438 | - | - | - | - | 140 | 376 | - | - | - | - | - | - | - | - | - | - | 326 | - |
|  |  | 15-19 years | 2438 | - | - | - | - | 163 | 448 | - | - | - | - | - | - | - | - | - | - | 289 | - |
|  |  | 20-25 years | 2592 | - | - | - | - | 158 | 372 | - | - | - | - | - | - | - | - | - | - | 204 | - |
|  |  | 25-30 years | 3403 | - | - | - | - | 216 | 471 | - | - | - | - | - | - | - | - | - | - | 282 | - |
|  |  | 30-35 years | 2118 | - | - | - | - | 142 | 285 | - | - | - | - | - | - | - | - | - | - | 186 | - |
|  |  | 35-39 years | 1836 | - | - | - | - | 111 | 230 | - | - | - | - | - | - | - | - | - | - | 140 | - |
|  |  | 40-49 years | 2272 | - | - | - | - | 121 | 293 | - | - | - | - | - | - | - | - | - | - | 187 | - |
|  |  | 50-59 years | 1193 | - | - | - | - | 67 | 159 | - | - | - | - | - | - | - | - | - | - | 89 | - |
|  |  | >59 years | 2234 | - | - | - | - | 111 | 266 | - | - | - | - | - | - | - | - | - | - | 286 | - |
| Tellevik et al (2015) | *G. lamblia, E. histolytica* | Out-patient | 701 | - | - | - | - | 0 | 24 | - | - | - | - | - | - | - | - | - | - | 140 | - |
|  |  | In-patient | 558 | - | - | - | - | 0 | 34 | - | - | - | - | - | - | - | - | - | - | 17 | - |
| Trainor et al (2016) | ETEC | - | 1941 | - | - | - | - | - | - | - | - | - | - | 201 | - | - | - | - | - | - | - |
| Prah et al (2021) | EIEC, EHEC, EPEC, STEC, ETEC, EAEC | - | 57 | - | - | - | - | - | - | - | 6 | 3 | 2 | 25 | 1 | - | - | - | - | - | - |
| Omolajaiye et al (2020) | EHEC, EIEC, EPEC | 0-11 months | 15 | - | - | - | - | - | - | - | - | 8 | 0 | - | 0 | - | - | - | - | - | - |
|  |  | 1-3 years | 31 | - | - | - | - | - | - | - | - | 6 | 2 | - | 0 | - | - | - | - | - | - |
|  |  | 3-5 years | 19 | - | - | - | - | - | - | - | - | 3 | 5 | - | 8 | - | - | - | - | - | - |
|  |  | 5-10 years | 5 | - | - | - | - | - | - | - | - | 1 | 1 | - | 2 | - | - | - | - | - | - |
|  |  | >10 years | 11 | - | - | - | - | - | - | - | - | 0 | 0 | - | 0 | - | - | - | - | - | - |
| Naguib et al (2018) | *Cryptosporidium spp., G. lamblia* | <3 years | 74 | - | - | - | 2 | - | - | - | - | - | - | - | - | - | - | - | - | 7 | - |
|  |  | 3-4 years | 141 | - | - | - | 3 | - | - | - | - | - | - | - | - | - | - | - | - | 20 | - |
|  |  | 4-5 years | 190 | - | - | - | 1 | - | - | - | - | - | - | - | - | - | - | - | - | 26 | - |
|  |  | 5-6 years | 136 | - | - | - | 2 | - | - | - | - | - | - | - | - | - | - | - | - | 10 | - |
|  |  | 6-7 years | 27 | - | - | - | 0 | - | - | - | - | - | - | - | - | - | - | - | - | 3 | - |
|  |  | 7-8 years | 17 | - | - | - | 0 | - | - | - | - | - | - | - | - | - | - | - | - | 0 | - |
| Bitilinyu-Bangoh et al (2019) | *Cryptosporidium spp., E. histolytica* | Malawi | 175 | - | - | - | 37 | 0 | - | - | - | - | - | - | - | - | - | - | - | - | - |
|  |  | Kenya | 120 | - | - | - | 7 | 0 | - | - | - | - | - | - | - | - | - | - | - | - | - |

# **Supplementary Table S4:** Pooled proportion estimates of the studies conducted in children <5 years of age, in an in-patient setting.

|  | **Studies with pop. <5 years of age in in-patient facilities** | | | | | |
| --- | --- | --- | --- | --- | --- | --- |
|  | Number of studies | Estimated pooled proportion | 95% CI | p-value (Wald-type, LR) | Heterogeneity ($\tau^{2}$) | Variability (I^2^) |
| Rotavirus (with rotavirus vaccine) | 4 | 0.278 | [0.185;0.396] | <0.0001, <0.0001 | 0.098 | 93.3% [86.1%; 96.8%] |
| Rotavirus (without rotavirus vaccine) | 6 | 0.234 | [0.137;0.37] | <0.0001, <0.0001 | 0.318 | 94.4% [90.7%; 96.6%] |
| Rotavirus without rotavirus vaccine (Infl. cases removed^*^) | 5 | 0.204 | [0.118;0.329] | <0.0001, <0.0001 | 0.225 | 91.4% [82.9%; 95.7%] |
| Norovirus | 11 | 0.076 | [0.049;0.116] | <0.0001, <0.0001 | 0.363 | 82.8% [70.5%; 89.9%] |
| Norovirus (excluding G1) | 11 | 0.074 | [0.049;0.111] | <0.0001, <0.0001 | 0.301 | 79.4% [63.8%; 88.3%] |
| Astrovirus | 7 | 0.017 | [0.004;0.079] | <0.0001, <0.0001 | 2.509 | 93.2% [88.5%; 96.0%] |
| Astrovirus (Infl. Cases removed^†^) | 6 | 0.012 | [0.003;0.04] | Wald: 0.6187; LRT: 0.0001 | 0.961 | 0.0% [0.0%; 74.6%] |
| *Campylobacter* spp. | 6 | 0.053 | [0.013;0.194] | <0.0001, <0.0001 | 1.609 | 93.5% [88.4%; 96.3%] |
| *Cryptosporidium* | 8 | 0.193 | [0.124;0.29] | <0.0001, <0.0001 | 0.357 | 87.3% [77.1%; 92.9%] |
| *Cryptosporidium* (Infl. cases removed^‡^) | 7 | 0.222 | [0.159;0.3] | <0.0001, <0.0001 | 0.165 | 83.8% [68.2%; 91.8%] |
| *E. histolytica* | 9 | 0.018 | [0.003;0.108] | <0.0001, <0.0001 | 4.596 | 91.6% [86.3%; 94.8%] |
| EHEC | 2 | 0.147 | [0;1] | Wald: 0.0004, LRT: 0.0002 | 2.679 | 91.9% [72.0%; 97.7%] |
| EPEC | 8 | 0.126 | [0.065;0.23] | <0.0001, <0.0001 | 0.605 | 86.5% [75.5%; 92.6%] |
| EPEC (excluding aPEC) | 9 | 0.181 | [0.06;0.43] | <0.0001, <0.0001 | 2.351 | 89.6% [82.5%; 93.8%] |
| EPEC (Infl. cases removed^§^) | 8 | 0.119 | [0.058;0.229] | <0.0001, <0.0001 | 0.849 | 87.9% [78.5%; 93.2%] |
| ETEC | 6 | 0.118 | [0.075;0.18] | <0.0001, <0.0001 | 0.158 | 84.3% [67.5%; 92.4%] |
| ETEC (excluding LT-ETEC) | 6 | 0.116 | [0.058;0.217] | <0.0001, <0.0001 | 0.428 | 94.1% [89.8%; 96.6%] |
| EAEC | 4 | 0.254 | [0.159;0.38] | Wald: 0.0006, LRT: 0.0004 | 0.094 | 82.7% [55.6%; 93.2%] |
| EIEC^**^ | 1 | 0.8 | [0.459;0.95] | - | - | - |
| STEC^**^ | 1 | 0.007 | [0.002;0.017] | - | - | - |
| *V. cholerae* | 4 | 0.023 | [0.006;0.082] | <0.0001, <0.0001 | 0.458 | 80.7% [49.5%; 92.7%] |
| *Salmonella* spp. | 4 | 0.016 | [0.003;0.074] | <0.0001, <0.0001 | 0.508 | 72.3% [21.7%; 90.2%] |
| *Shigella* spp. | 6 | 0.071 | [0.029;0.166] | <0.0001, <0.0001 | 0.724 | 90.5% [82.0%; 95.0%] |
| *G. lamblia* | 9 | 0.154 | [0.084;0.264] | <0.0001, <0.0001 | 0.753 | 97.2% [96.0%; 98.0%] |
| *G. lamblia* (Infl. cases removed^¶^) | 6 | 0.123 | [0.07;0.206] | <0.0001, <0.0001 | 0.315 | 95.5% [92.5%; 97.3%] |
| Other | 29 | 0.122 | [0.082;0.177] | <0.0001, <0.0001 | 0.234 | 97.8% [97.4%; 98.2%] |
| Other (Infl. cases removed^\|\|^) | 18 | 0.093 | [0.067;0.126] | <0.0001, <0.0001 | 0.349 | 88.7% [83.7%; 92.2%] |
| Unknown | 3 | 0.225 | [0.021;0.798] | <0.0001, <0.0001 | 1.081 | 98.5% [97.4%; 99.2%] |
| ^*^Removed as outliers: Nhampossa (2015), 24-59mo  ^†^Removed as outliers: Arowolo (2019)  ^‡^Removed as outliers: Bitilinyu-Bangoh (2019)  ^§^Removed as outliers: Acaio (2019)  ^¶^Removed as outliers: Nhampossa (2015), 24-59mo; Lompo (2021); Tellevik (2015)  ^\|\|^Removed as outliers: Quédraogo (2016); Japhet (2019); Gasparinho (2016); Nhampossa, group 1(2015); Iturizza-Gómara, group 1 (2019); Iturizza-Gómara, group 2 (2019); Gosselin (2018); Garzón, 13-16mo (2017); Garzón, 17-18mo (2017); Garzón, 19-24mo (2017); Tellevik (2015)  ^**^Point prevalence (Only 1 study available), CI is approximated | | | | | | |

# **Supplementary Table S5:** Pooled proportion estimates of the studies conducted in children ≥5 years of age and adults, in an in-patient setting.

|  | **Studies with pop.** ≥**5 years of age in in-patient facilities** | | | | | |
| --- | --- | --- | --- | --- | --- | --- |
|  | Number of studies | Estimated pooled proportion | 95% CI | p-value (Wald-type, LR) | Heterogeneity ($\tau^{2}$) | Variability (I^2^) |
| Rotavirus^*^  (with rotavirus vaccine) | 1 | 0.359 | [0.242;0.495] | - | - | - |
| Rotavirus (without rotavirus vaccine) | 0 | - | - | - | - | - |
| Norovirus | 0 | - | - | - | - | - |
| Norovirus (excluding G1) | 0 | - | - | - | - | - |
| Astrovirus | 0 | - | - | - | - | - |
| *Campylobacter* spp. | 0 | - | - | - | - | - |
| *Cryptosporidium* spp. ^*^ | 1 | 0.057 | [0.012;0.157] | - | - | - |
| *E. histolytica* | 11 | 0.06 | [0.056;0.064] | <0.0001, <0.0001 | 0.003 | 57.9% [17.8%; 78.5%] |
| *E. histolytica* (Infl. cases removed^†^) | 10 | 0.059 | [0.056;0.063] | <0.0001, <0.0001 | 0.001 | 24.8% [0.0%; 63.6%] |
| EHEC* | 1 | 0.143 | [0.02;0.581] | - | - | - |
| EPEC | 1 | 0.143 | [0.02;0.581] | - | - | - |
| EPEC (excluding aEPEC)* | 1 | 0.143 | [0.02;0.581] | - | - | - |
| ETEC | 0 | - | - | - | - | - |
| ETEC (excluding LT-ETEC) | 0 | - | - | - | - | - |
| EAEC | 0 | - | - | - | - | - |
| EIEC* | 1 | 0.286 | [0.072;0.673] | - | - | - |
| STEC | 0 | - | - | - | - | - |
| *V. cholerae* | 0 | - | - | - | - | - |
| *Salmonella* spp. | 0 | - | - | - | - | - |
| *Shigella* spp. | 0 | - | - | - | - | - |
| *G. lamblia* | 11 | 0.129 | [0.1;0.164] | <0.0001, <0.0001 | 0.162 | 91.4% [86.6%; 94.5% |
| *G. lamblia* (Infl. Cases removed^‡^) | 8 | 0.135 | [0.126;0.144] | Wald: 0.0202, LRT: 0.0202 | 0.004 | 57.8% [7.6%; 80.8%] |
| Other | 16 | 0.101 | [0.066;0.15] | <0.0001, <0.0001 | 0.662 | 97.6% [97.0%; 98.1%] |
| Other (Infl. cases removed) | 13 | 0.096 | [0.082;0.112] | <0.0001, <0.0001 | 0.065 | 92.8% [89.5%; 95.1%] |
| Unknown* | 1 | 0.595 | [0.536;0.653] | - | - | - |
| *Point prevalence (Only 1 study available), CI is approximated  ^†^Removed as outliers: Njaguna (2016), All ages  ^‡^Removed as outliers: Njaguna (2016), All ages; Ramos (2014), 5-9y; Ramos, (2014), 15-19y | | | | | | |

# **Supplementary Table S6:** Pooled proportion estimates of the studies conducted in children <5 years of age, in an out-patient and community settings.

|  | **Studies with pop. <5 years of age in out-patient facilities/communities** | | | | | |
| --- | --- | --- | --- | --- | --- | --- |
|  | Number of studies | Estimated pooled proportion | 95% CI | p-value (Wald-type, LR) | Heterogeneity ($\tau^{2}$) | Variability (I^2^) |
| Rotavirus (with rotavirus vaccine) | 8 | 0.048 | [0.009;0.219] | <0.0001, <0.0001 | 3.782 | 93.3% [89.1%; 95.9%] |
| Rotavirus (Infl. cases removed^*^) | 7 | 0.035 | [0.021;0.058] | <0.0001, <0.0001 | 3.57 | 93.3% [88.7%; 96.1%] |
| Rotavirus (without rotavirus vaccine) | 27 | 0.214 | [0.141;0.312] | <0.0001, <0.0001 | 1.451 | 97.7% [97.2%; 98.1%] |
| Rotavirus (Infl. cases removed^†^) | 21 | 0.179 | [0.138;0.228] | <0.0001, <0.0001 | 0.319 | 92.6% [90.1%; 94.5%] |
| Norovirus | 28 | 0.103 | [0.065;0.159] | <0.0001, <0.0001 | 1.412 | 96.9% [96.2%; 97.4%] |
| Norovirus (excluding G1) | 28 | 0.103 | [0.065;0.159] | <0.0001, <0.0001 | 1.402 | 96.8% [96.1%; 97.4%] |
| Norovirus (Infl. cases removed^‡^) | 18 | 0.087 | [0.061;0.121] | <0.0001, <0.0001 | 0.339 | 76.6% [63.3%; 85.1%] |
| Astrovirus | 14 | 0.03 | [0.017;0.051] | <0.0001, <0.0001 | 0.585 | 88.4% [82.2%; 92.4%] |
| Astrovirus (Infl. cases removed^§^) | 12 | 0.021 | [0.015;0.031] | Wald: 0,1088, LRT: 0,1187 | 0.103 | 35.2% [0.0%; 67.3%] |
| *Campylobacter* spp. | 16 | 0.052 | [0.02;0.129] | <0.0001, <0.0001 | 3.036 | 98.7% [98.4%; 98.9%] |
| *Campylobacter* spp. (Infl. cases removed^¶^) | 11 | 0.041 | [0.024;0.068] | <0.0001, <0.0001 | 0,044 | 75.2% [55.2%; 86.3%] |
| *Cryptosporidium* | 29 | 0.058 | [0.04;0.083] | <0.0001, <0.0001 | 0.793 | 89.5% [86.0%; 92.1%] |
| *Cryptosporidium* (Infl. cases removed^\|\|^) | 19 | 0.047 | [0.032;0.067] | Wald: 0.0155, LRT: <0.0001 | 0.266 | 45.9% [7.4%; 68.4%] |
| *E. histolytica* | 17 | 0.006 | [0.001;0.028] | <0.0001, <0.0001 | 4.804 | 79.5% [67.9%; 86.9%] |
| *E. histolytica* (Infl. cases removed^**^) | 13 | 0.003 | [0.001;0.014] | Wald: 0.6369, LRT: 0.0002 | 2.249 | 0.0% [0.0%; 56.6%] |
| EHEC | 5 | 0.033 | [0.011;0.097] | Wald: 0.9872, LRT: 0.5550 | 0 | 0.0% [0.0%; 79.2%] |
| EPEC | 14 | 0.067 | [0.047;0.096] | 0.0001, <0.0001 | 0.224 | 68.1% [44.4%; 81.7%] |
| EPEC (excluding aEPEC) | 15 | 0.066 | [0.035;0.12] | <0.0001, <0.0001 | 1.127 | 92.2% [88.8%; 94.6%] |
| EPEC (Infl. cases removed^††^) | 13 | 0.059 | [0.043;0.081] | Wald: 0.3138; LRT: 0.0729 | 0.052 | 13.0% [0.0%; 52.2%] |
| ETEC | 21 | 0.095 | [0.068;0.131] | <0.0001, <0.0001 | 0.487 | 89.2% [84.9%; 92.3%]; |
| ETEC (excluding LT-ETEC) | 25 | 0.091 | [0.06;0.135] | 0 | 1.005 | 99.1% [99.0%; 99.2%] |
| ETEC (Infl. cases removed^‡‡^) | 18 | 0.087 | [0.067;0.112] | <0.0001, <0.0001 | 0.191 | 69.1% [49.9%; 81.0%] |
| EAEC | 14 | 0.155 | [0.097;0.24] | <0.0001, <0.0001 | 0.712 | 93.3% [90.3%; 95.3%] |
| EAEC (Infl. cases removed^§§^) | 10 | 0.142 | [0.096;0.204] | Wald: 0.0086; LRT: 0.0002 | 0.202 | 59.3% [18.2%; 79.7%] |
| EIEC | 3 | 0.021 | [0.001;0.429] | Wald: 0.0068; LRT: <0.0001 | 1.369 | 80.0% [36.7%; 93.7%] |
| STEC | 8 | 0.041 | [0.007;0.204] | <0.0001, <0.0001 | 3.951 | 77.3% [55.1%; 88.6%] |
| *V. cholerae* | 4 | 0.002 | [0;0.089] | Wald: 0.2573, LRT: 0.0002 | 2.848 | 25.7% [0.0%; 71.6%] |
| *Salmonella* spp. | 11 | 0.012 | [0.005;0.033] | <0.0001, <0.0001 | 1.586 | 85.4% [75.6%; 91.3%] |
| *Salmonella* spp. (Infl. cases removed^¶¶^) | 10 | 0.01 | [0.005;0.02] | Wald: 0,0906; LRT: 0,0054 | 0.477 | 40.1% [0.0%; 71.4%] |
| *Shigella* spp. | 15 | 0.019 | [0.01;0.037] | <0.0001, <0.0001 | 1.158 | 90.7% [86.4%; 93.7%] |
| *Shigella* spp. (Infl. cases removed^\|\|\|^) | 12 | 0.019 | [0.01;0.035] | <0.0001, <0.0001 | 0.696 | 81.2% [68.2%; 88.9%] |
| *G. lamblia* | 21 | 0.13 | [0.086;0.193] | <0.0001, <0.0001 | 0.959 | 96.1% [95.1%; 97.0%] |
| *G. lamblia* (Infl. cases removed^***^) | 17 | 0.132 | [0.097;0.177] | <0.0001, <0.0001 | 0.358 | 80.2% [69.1%; 87.3%] |
| ^*^Removed as outliers: Ashie (2017), 13-24mo  ^†^Removed as outliers: Quédraogo (2016); Japhet (2019), 0-5mo; Japhet (2019), 6-11mo; Japhet (2019), 12-23mo; Zimmermann (2019); Mansour (2014)  ^‡^Removed as outliers: Zimmermann, Kenya (2019); Zimmermann, Nigeria (2019); Zimmermann, Mali (2019); Acacio (2019); Nhampossa, 0-11mo (2015); Platts-Mills, 0-11mo (2015); Platts-Mills, 12-24mo (2015); Chuckwu (2020); Imade, 0-6mo (2015); Imade, 7-12mo (2015)  ^§^Removed as outliers: Japhet, 12-23mo(2019); Hungerford (2014)  ^¶^Removed as outliers: Hungerford (2014); Nhampossa, 0-11mo(2015); Platts-Mills, 0-11mo (2015); Chuckwu (2020)  ^\|\|^Removed as outliers: Zimmermann, Kenya (2019); Gasparinho (2016); Hungerford (2014); Acacio (2019); Bauhofer, 0-11mo(2020); Bauhofer, 12-23mo(2020); Naguib, 4-5y (2018)  ^**^Removed as outliers: Acacio (2019); Nhampossa (2015), 0-11mo; Nhampossa (2015), 12-23mo; Nhampossa (2015), 24-59mo  ^††^Removed as outliers: Lijima (2017)  ^‡‡^Removed as outliers: Gasparinho (2016); Acacio (2019); Nhampossa, 0-11mo (2015); Nhampossa, 12-23mo (2015); Hassan (2014); Prah (2021); Zimmermann, Kenya (2019)  ^§§^Removed as outliers: Gasparinho (2016); Acacio (2019); Nhampossa, 0-11mo (2015); Lijima (2017)  ^¶¶^Removed as outliers: Ashie, 25-60mo (2017)  ^\|\|\|^Removed as outliers: Acacio, (2019); Nhampossa, 0-11mo (2015); Iturriza-Gómara (2019)  ^***^Removed as outliers: Nhampossa, 12-23mo (2015); Nhampossa, 24-59mo (2015); Ashie, group 2 (2017); Tellevik (2015) | | | | | | |

# **Supplementary Table S7:** Pooled proportion estimates of the studies conducted in children ≥5 years of age and adults, in an out-patient and community settings.

|  | **Studies with pop.** ≥**5 years of age in out-patient facilities/communities** | | | | | |
| --- | --- | --- | --- | --- | --- | --- |
|  | Number of subgroups | Estimated pooled proportion | 95% CI | p-value (Wald-type, LR) | Heterogeneity ($\tau^{2}$) | Variability (I^2^) |
| Rotavirus (with rotavirus vaccine) | 0 | - | - | - | - | - |
| Rotavirus (without rotavirus vaccine) | 0 | - | - | - | - | - |
| Norovirus | 0 | - | - | - | - | - |
| Norovirus (excluding G1) | 0 | - | - | - | - | - |
| Astrovirus | 0 | - | - | - | - | - |
| *Campylobacter* spp. | 0 | - | - | - | - | - |
| *Cryptosporidium* | 6 | 0.019 | [0.007;0.052] | Wald: 0.2302; LRT: 0.035 | 0.29 | 27.3% [0.0%; 69.9%] |
| *E. histolytica* | 7 | 0.054 | [0.011;0.226] | <0.0001, <0.0001 | 2.798 | 91.6% [85.2%; 95.2%] |
| EHEC | 0 | - | - | - | - | - |
| EPEC | 1 | 0.028 | [0.011;0.056] | - | - | - |
| EPEC (excluding aPEC)* | 1 | 0.056 | [0.023;0.111] | - | - | - |
| ETEC | 1 | 0.794 | [0.052;0.12] | - | - | - |
| ETEC (excluding LT-ETEC)* | 1 | 0.159 | [0.1;0.234] | - | - | - |
| EAEC* | 1 | 0.087 | [0.044;0.151] | - | - | - |
| EIEC* | 1 | 0.016 | [0.002;0.056] | - | - | - |
| STEC* | 1 | 0.143 | [0.087;0.216] | - | - | - |
| *V. cholerae* | 0 | - | - | - | - | - |
| *Salmonella* spp. | 2 | 0.011 | [0;1] | Wald: 0.9996: LRT: <0.0001 | 4.591 | 0% |
| *Shigella* spp. | 2 | 0.019 | [0;0.523] | Wald: 0.395; LRT: 0.3393 | 0 | 0% |
| *G. lamblia* | 6 | 0.147 | [0.056;0.333] | <0.0001, <0.0001 | 0.912 | 88.4% [77.3%; 94.1%] |
| *Point prevalence (Only 1 study available), CI is approximated | | | | | | |

# **Supplementary Table S8:** Pooled proportion estimates of norovirus, EPEC and ETEC before and after adjusting for potential carriage of pathogens unrelated to diarrhea.

|  | In-patient setting | | Out-patient and community setting | |
| --- | --- | --- | --- | --- |
|  | <5 | ≥5 | <5 | ≥5 |
| Norovirus | 0.076 | - | 0.103 | - |
| Norovirus (excluding G1) | 0.074 | - | 0.087 | - |
| ETEC | 0.118 | - | 0.095 | 0.794 |
| ETEC (excluding LT-ETEC) | 0.116 | - | 0.091 | 0.159 |
| EPEC | 0.126 | 0.143 | 0.067 | 0.028 |
| EPEC (excluding aEPEC) | 0.181 | 0.143 | 0.066 | 0.056 |

# **Supplementary Table S9:** P-value obtained from the Egger’s test testing for asymmetry, with number of studies (n). InS = Insufficient number of trials (n<10)

|  | In-patient studies | | Out-patient and community studies | |
| --- | --- | --- | --- | --- |
| Pathogen | Population <5 years | Population ≥5 years | Population <5 years | Population ≥5 years |
| Rotavirus (vaccination) | InS (n = 4) | InS (n = 1) | InS (n = 8) | - |
| Rotavirus (no vaccination) | InS (n = 6) | - | p-value: 0.4078 (n = 27) | - |
| Norovirus | p-value: 0.0199 (n = 11) | - | p-value: 0.4269 (n = 28) | - |
| Astrovirus | InS (n = 7) | - | p-value: 0.2352 (n = 14) | - |
| *Campylobacter* spp. | InS (n = 6) | - | p-value: 0.6471 (n = 16) | - |
| *Cryptosporidium* spp. | InS (n = 8) | InS (n = 1) | p-value: 0.0032 (n = 29) | InS (n = 6) |
| *Entamoeba histolytica* | InS (n = 9) | p-value: 0.2683 (n = 11) | p-value: <0.0001 (n = 17) | InS (n = 7) |
| *Giardia lamblia* | InS (n = 9) | InS (n = 1) | p-value: 0.0413 (n = 21) | InS (n = 6) |
| EHEC | InS (n = 2) | InS (n = 1) | InS (n = 5) | - |
| EPEC | InS (n = 9) | InS (n = 1) | p-value: 0.4401 (n = 15) | InS (n = 1) |
| ETEC | InS (n = 6) | - | p-value: 0.0336 (n = 25) | InS (n = 1) |
| EAEC | InS (n = 4) | - | p-value: 0.0243 (n = 14) | InS (n = 1) |
| EIEC | InS (n = 1) | InS (n = 1) | InS (n = 3) | InS (n = 1) |
| STEC | InS (n = 1) | - | InS (n = 8) | InS (n = 1) |
| *Vibrio cholerae* | InS (n = 4) | - | InS (n = 4) | - |
| *Salmonella* spp. | InS (n = 4) | InS (n = 1) | p-value: 0.2398 (n = 11) | InS (n = 2) |
| *Shigella* spp. | InS (n = 6) | InS (n = 1) | p-value: 0.9785 (n = 15) | InS (n = 2) |

# **Supplementary Table S10:** Incidence and population estimates from Desta et al.^14^ and WHO Global Health Observatory

| Incidence (per person-year) | | | | | | |
| --- | --- | --- | --- | --- | --- | --- |
|  |  | Overall | Ethiopia | Mozambique | Nigeria | Tanzania |
| Annual rate  Age-standardized^*^ | | 0.8 (0.51- 1.07) | 1.25 (0.22- 1.92) | 1.85 (0.63- 2.8) | 0.33 (0.22- 0.43) | 0.42 (0.22-0.6) |
| Population estimates (2019) (people-per-year) | | | | | | |
|  |  | Northern Africa^†^ | Central Africa^‡^ | Eastern Africa^§^ | Southern Africa^¶^ | Western Africa^\|\|^ |
| Children <5 | | 29,097,112 | 30,190,286 | 66,871,697 | 6,788,604 | 64,211,594 |
| Children ≥ 5 and adults | | 212,101,195 | 144,118,141 | 365,878,161 | 59,841,290 | 327,222,492 |
| Total population | | 241,198,307 | 174,308,427 | 432,749,858 | 66,629,894 | 391,434,086 |

^*^Nigerian census population age proportion was used to standardize the annual incidence rates, given its larger population than the other study countries

^†^Northern Africa (6): Algeria, Egypt, Libya, Morocco, Sudan, Tunisia

^‡^Central Africa (9): Angola, Cameroon, Central African Republic, Chad, Congo Republic - Brazzaville, Democratic Republic of Congo, Equatorial Guinea, Gabon, Sao Tome & Principe

^§^Eastern Africa (18): Burundi, Comoros, Djibouti, Ethiopia, Eritrea, Kenya, Madagascar, Malawi, Mauritius, Mozambique, Rwanda, Seychelles, Somalia, South Sudan, United Republic of Tanzania, Uganda, Zambia, Zimbabwe

^¶^Southern Africa (5): Botswana, Lesotho, Namibia, South Africa, Eswatini

^||^Western Africa (16): Benin, Burkina Faso, Cote D'Ivoire, Gambia, Ghana, Guinea, Guinea-Bissau, Liberia, Mali, Mauritania, Niger, Nigeria, Senegal, Sierra Leone, Togo

# **Supplementary Table S11:** Incidence and mortality envelope obtained from Global Burden of Disease incidence and mortality envelopes, 2019. Country-specific data were extracted from <https://vizhub.healthdata.org/gbd-compare/>.

| Incidence and mortality envelopes (2019) (people-per-year) | | | | | | | | | | |
| --- | --- | --- | --- | --- | --- | --- | --- | --- | --- | --- |
| Incidence envelope | | Northern Africa^†^ | | Central Africa^‡^ | | Eastern Africa^§^ | Southern Africa^¶^ | | | Western Africa^\|\|^ |
| Children <5 | | 54,118,754 (44,642,278-63,755,481) | | 67,208,529 (54,981,547-78,320,809) | | 127,161,099 (102,961,981-152,716,741) | 5,817,908 (4,618,025-7,208,674) | | | 139,842,581 (113,593,845-166,199,702) |
| Children ≥ 5 and adults | | 132,015,596 (112,481,665-153,420,589) | | 107,681,116 (94,177,091-122,101,397) | | 260,481,016 (227,412,444-296,049,024) | 46,444,006 (41,043,360-52,339,235) | | | 273,022,819 (239,146,168-309,198,041) |
| Mortality envelope | | Northern Africa^†^ | | Central Africa^‡^ | | Eastern Africa^§^ | Southern Africa^¶^ | | | Western Africa^\|\|^ |
| Children <5 | | 14,302 (6,630-25,444) | | 71,015 (32,594-123,823) | | 85,596 (48,761-137,343) | 4,588 (3,141-6,515) | | | 194,960 (127,626-286,186) |
| Children ≥ 5 and adults | | 2,643 (1,141-5,113) | | 31,042 (15,756-56,442) | | 86,711 (42,782-150,149) | 11,589 (6,330-21,846) | | | 80,779 (41,962-147,753) |
| Population estimates (2019) (people-per-year) | | | | | | | | | | |
|  | Northern Africa^†^ | | Central Africa^‡^ | | Eastern Africa^§^ | | | Southern Africa^¶^ | Western Africa^\|\|^ | |
| Children <5 | 24,959,398 | | 28,263,473 | | 66,268,542 | | | 5,986,798 | 65,167,747 | |
| Children ≥ 5 and adults | 211,025,201 | | 148,987,192 | | 361,569,179 | | | 57,557,168 | 345,432,446 | |
| Total population | 235,984,599 | | 177,250,665 | | 427,837,721 | | | 63,543,966 | 410,600,193 | |

^†^Northern Africa (6): Algeria, Egypt, Libya, Morocco, Sudan, Tunisia

^‡^Central Africa (9): Angola, Cameroon, Central African Republic, Chad, Congo Republic - Brazzaville, Democratic Republic of Congo, Equatorial Guinea, Gabon, Sao Tome & Principe

^§^Eastern Africa (18): Burundi, Comoros, Djibouti, Ethiopia, Eritrea, Kenya, Madagascar, Malawi, Mauritius, Mozambique, Rwanda, Seychelles, Somalia, South Sudan, United Republic of Tanzania, Uganda, Zambia, Zimbabwe

^¶^Southern Africa (5): Botswana, Lesotho, Namibia, South Africa, Eswatini

^||^Western Africa (16): Benin, Burkina Faso, Cote D'Ivoire, Gambia, Ghana, Guinea, Guinea-Bissau, Liberia, Mali, Mauritania, Niger, Nigeria, Senegal, Sierra Leone, Togo
